# Mechanochemical Amidation Mediated by 1,1’‐Oxalyldiimidazole for the Synthesis of Agrochemicals

**DOI:** 10.1002/chem.71190

**Published:** 2026-05-29

**Authors:** Andrea Casagrande, Rubén Solorzano‐Rodriguez, Maria Carta, Francesco Delogu, Joris Vezzani, Allan Niidu, Evelina Colacino

**Affiliations:** ^1^ ICGM, CNRS, ENSCM Univ. Montpellier Montpellier France; ^2^ Virumaa College Tallinn University of Technology Tallinn Estonia; ^3^ Department of Mechanical Chemical and Materials Engineering University of Cagliari Cagliari Italy

**Keywords:** 1,1’‐carbonyl diimidazole (CDI), 1,1’‐oxalyl diimidazole (ODI), agrochemicals, amide bond, ball‐milling, energy, green metrics

## Abstract

The synthesis of amide moieties, essential components in both pharmaceuticals and agrochemicals, traditionally involves environmentally hazardous and polluting processes. This study presents a novel mechanochemical method to form amide bond using 1,1’‐oxalyl diimidazole (ODI) as activating agent, and evaluates its applicability in synthesizing agrochemicals such as Mepronil, Flutolanil, and Fluxapyroxad at gram‐scale. The mechanochemical kinetics underlying the synthesis of the three mentioned compounds was also investigated. The new method was compared with the similar 1,1’‐carbonyl diimidazole (CDI)‐mediated mechanochemical amidation previously reported. Results demonstrate that ODI‐mediated synthesis outperforms in the case of deactivated acids. Energetic considerations and technical process parameters are also discussed to provide quantitative technology assessment. A comprehensive analysis by green chemistry metrics with CHEM21 and DOZN^TM^ 3.0 toolkits, underscores the potential benefits of the mechanochemical approach, compared with the solvent‐based protocols for the preparation of marketed agrochemicals.

## Graphical abstract

Mechanochemical amidation using 1,1'‐oxalyldiimidazole (ODI) overcomes limitations of 1,1’‐carbonyl diimidazole (CDI) as coupling agent by enabling the efficient mechanochemical synthesis of three marketed agrochemicals from deactivated carboxylic acids. Kinetic investigations were performed and the mechanochemical methods were assessed by against traditional solution‐based methods by green chemistry metrics (using CHEM21 and DOZN 3.0 tools), demonstrating better eco‐footprint.

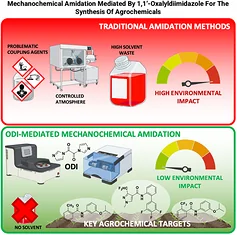

## 1 Introduction

The synthesis of amide bonds is one of the most used transformations in industry [1], with applications encompassing organic synthesis and material functionalization [2], and widely used also in drug discovery [3] and in the preparation of active pharmaceutical ingredients [4] and crop protecting agrochemicals [5]. The efforts undertaken to discover active compounds for crop protection have recently intensified with the objectives of enhancing the selective control of insects, weeds, and fungal pathogens as well as reducing toxicological and environmental concerns [6]. This is part of the strategies aimed at improving crop yields to account for the increase in global population and the changes in regulation [7, 8]. Developing efficient and sustainable synthetic routes to access strategic agrochemicals cannot be postponed any longer in the light of the upward jump in global agricultural pesticide production rose from 1.80 million metric tons in 1990 to 3.73 million metric tons of active ingredients in 2023 [9].

In this regard, the superior green performance [10], reduced energy demand [11], minimized waste production, and avoidance of hazardous solvents [12, 13] exhibited by mechanochemical methods show great promise. They meet not only the criteria of Safe and Sustainable by Design (SSbD), but are consistent with the recently launched *IUPAC Guiding Principles of Responsible Chemistry* [14].

**SCHEME 1:**
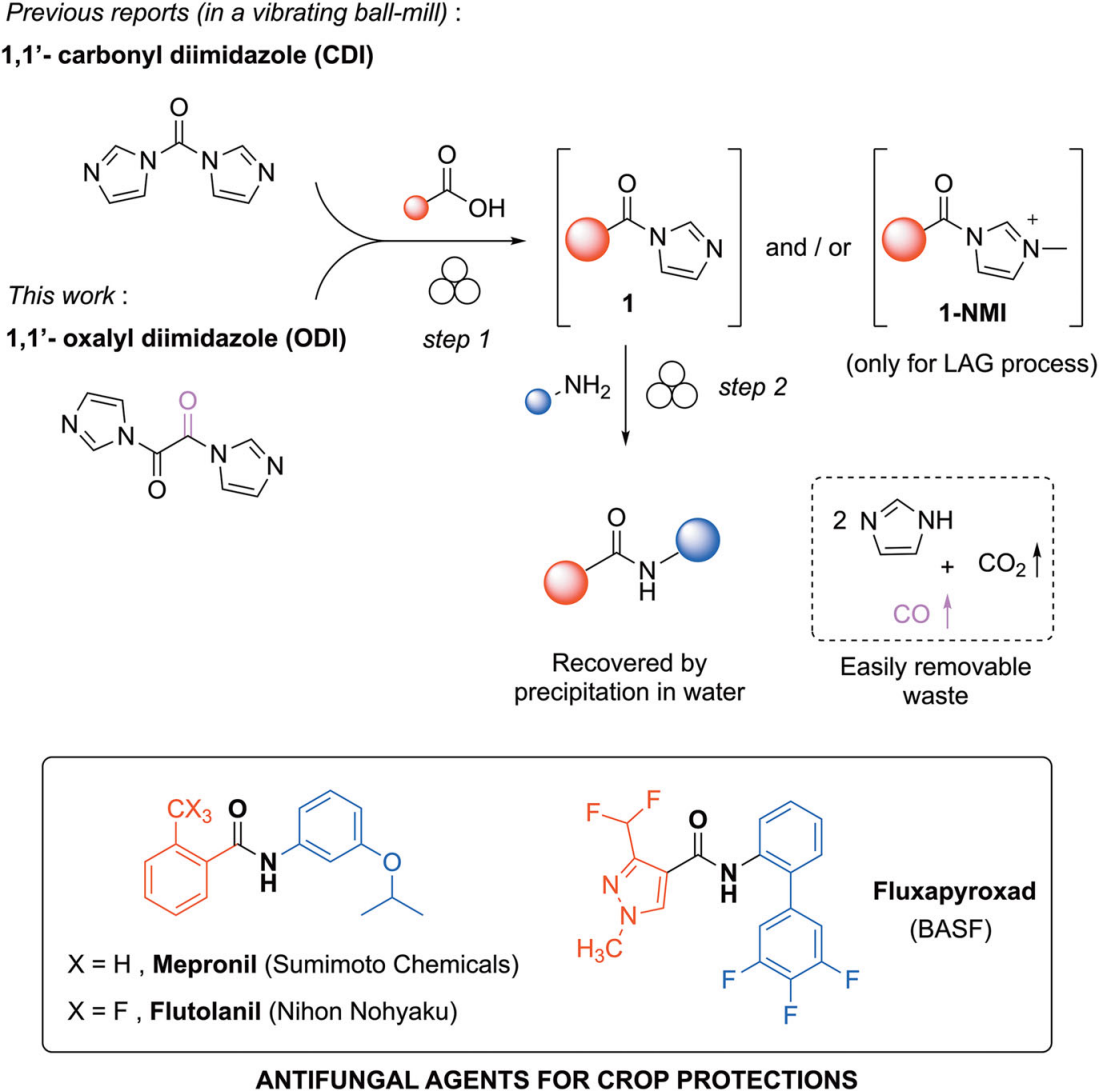
Activating agents CDI and ODI and preparation of marketed agrochemicals by amidation reaction.

In this work, we propose exactly a mechanochemical synthesis of three antifungal agents marketed for crop protection, Mepronil [15], Flutolanil [16, 17], and Fluxapyroxad [18, 19], with a high global market size. As evident from Scheme 1, the key step is an amidation reaction. Generally, amide bond formation remains challenging because carboxylic acid activation often depends on coupling/activating reagents that present either safety concerns or poor sustainability [2]. Chlorinating agents such as SOCl_2_, PCl_5_, and POCl_3_ are air‐ and moisture‐sensitive and may release toxic, corrosive byproducts, including SO_2_ and HCl, during the reaction, while widely used new generation uronium and phosphonium coupling reagents, such as hexafluorophosphate azabenzotriazole tetramethyl uronium (HATU), hexafluorophosphate benzotriazole tetramethyl uronium (HBTU), (benzotriazol‐1‐yloxytripyrrolidinophosphonium hexafluorophosphate (PyBOP), and bromo(tripyrrolidin‐1‐yl)phosphanium hexafluorophosphate (PyBrOP), despite their efficiency typically produce significant stoichiometric waste, resulting in low atom economy and difficult purification. Compared with their current industrial syntheses, which require hazardous and/or polluting compounds, controlled atmosphere, heavy purification procedures, mechanochemical processes can be expected to provide an improved eco‐footprint [10, 20, 21, 22, 23]. The advantages offered by mechanochemical processing go well beyond solvent avoidance. For instance, the experimental set‐up is simple, new reactivities can be explored [24], solubility, or steric hindrance [25] of the reactants are no longer an issue, chemical space can be expanded [26], and reaction rates can be higher [27, 28, 29, 30, 31, 32].

Additional advantage come from the choice of reagents for amidation reactions under mechanochemical processing conditions [33]. A safer alternative to the hazardous isocyanates, (tri)phosgene, or chloroformates typically used in the industry, is 1,1’‐carbonyl diimidazole (CDI), which is a highly efficient and user‐friendly acyl transfer agent, affordable and readily available in bulk quantities, making it ideal for large‐scale applications. It is also a valid alternative to the most commonly used coupling/activating agents that, despite their efficiency, display poor atom economy.

CDI has been effectively employed in the solution synthesis of carbonates, ureas, amides, urethanes, and esters [34, 35, 36], as well as in peptide coupling reactions [37, 38, 39, 40, 41]. In pharmaceutics, CDI is particularly valued for its ability to produce APIs in a clean environment [42, 43], since its byproducts—imidazole and carbon dioxide—are relatively benign and easily removed. Moreover, compared with other coupling agents currently used in amide synthesis, associated waste is less harmful, and the use of resources is improved (e.g., better atom economy). CDI was also successfully used in the mechanochemical preparation of amides [44], ureas [45, 46, 47, 48], (including the Lossen rearrangement to hydantoins) [49] and carbamates [50].

Since CDI becomes less efficient with poorly nucleophilic, deactivated acids, the use of 1,1’‐oxalyldiimidazole [51] (ODI) was explored. ODI is a more reactive analogue of CDI, and particularly suitable to react with carboxylic acid having unfavorable electronic properties, which fail to deliver the activated *N*‐acyl imidazole intermediate **1** (*first step*, Scheme 1) when CDI is used.

In this respect, it is worth noting that very few reports deal with the use of ODI in solution to prepare carboxylic acid anhydrides [52], esters (e.g., 1,4‐dihydroquinoxaline‐2,3‐diones) [53], nitriles, formiates and formamides [52], cyclic hydrazides (e.g., piperazine‐2,3‐diones and their tautomers) [54, 55], or as a ligand in inorganic synthesis [56] and for chemiluminescent detection [57]. Surprisingly, no reports deal either with the use of ODI‐mediated synthesis of amide in solution nor by mechanochemical processing.

**FIGURE 1:**
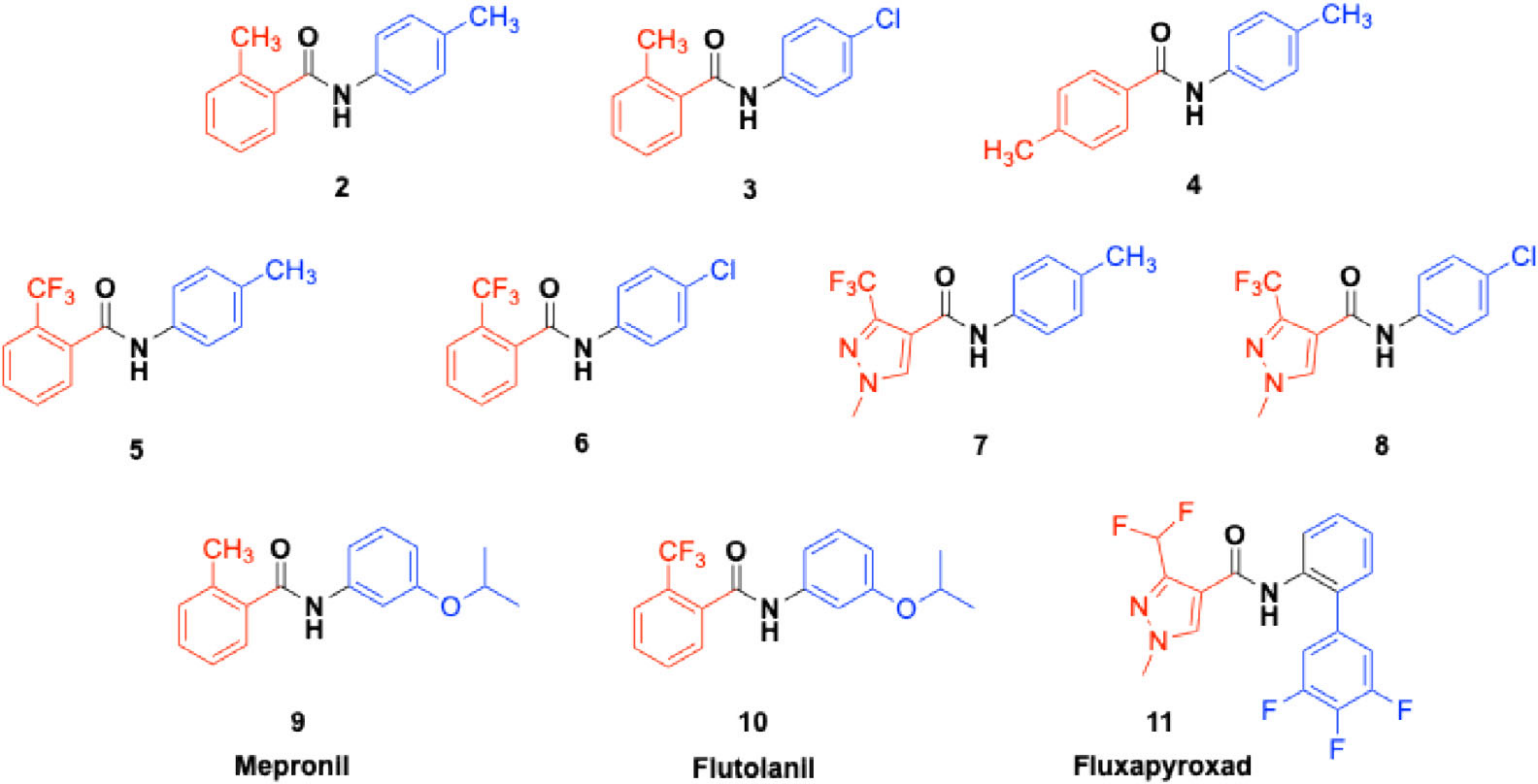
Reaction scope to compare the efficiency of CDI versus ODI‐mediated synthesis of amides by mechanochemical processing.

We used the new mechanochemical method we developed to prepare three marketed agrochemicals (Scheme 1), and extended the reaction scope (Figure 1), to better compare the efficiency of CDI‐ vs ODI‐mediated mechanochemical synthesis of amides (see Table S3, Supporting Information for a comparison of CDI vs ODI in solution‐based synthesis). The results are discussed in terms of technical and processing parameters, and corresponding energy inputs [58].

The ecological footprint of the ODI‐mediated syntheses for three marketed agrochemicals was also evaluated by green chemistry metrics [10], and assessed by CHEM21 [59] and DOZN [12, 60, 61] toolkits. The scores with the solution‐based manufacturing processes, and with the corresponding CDI‐mediated syntheses by mechanochemistry.

## 2 Results and Discussion

1,1′‐Carbonyldiimidazole (CDI)‐mediated activation to amides. The CDI‐mediated preparation of selected targets, including the agrochemicals Mepronil (**9**), Flutolanil (**10**), and Fluxapyroxad (**11**) (Scheme 1 and Figure 1) was explored first, to fully enlighten and disclose similarities and differences with their corresponding ODI‐mediated reactions. Table 1 sums‐up the best mechanochemical reaction conditions identified for each target.

**TABLE 1:** Reaction scope for CDI‐mediated mechanochemical syntheses of amides **2–11** in vibrating ball‐mills, in different processing conditions.^a^

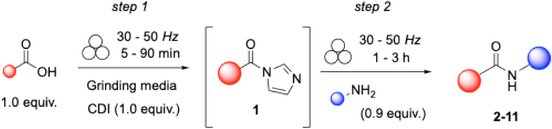				
		Yield (%)		
Entry^b^	Compound	ZrO_2_ ^b^	SS^b^	PTFE^c^
1	**2**	**98**	94	96
2	**3**	84	60	**87**
3	**4**	**90**	n.p.^d^	n.p.^d^
4	**5**	**82**	73	70
5	**6**	0	0	**60**
6	**7**	91	**94**	63
7	**8**	**85**	67	71
8	**9**	66	**70**	61
9	**10**	**50**	7	32
10	**11**	0	0	n.p^d^

^a^ Typical experimental procedure: the acid (1.0 equiv.) and the 1,1’‐carbonyldiimidazole (CDI, 1.0 equiv.) were milled at a given frequency (30 or 50 *Hz*), during 5–90 min. (*step 1*). The amine (0.9 equiv.) was added, and the mixture was milled further for 1–3 h (*step 2*). The reaction scale was 1.35 mmol, except when otherwise specified (*c.f*., the detailed mechanochemical process conditions are reported in the Supporting Information and Table S2).

^b^ Horizontal vibrating ball‐mill at 30 *Hz* was used.

^c^ Vertical vibrating mill at 50 *Hz* was used.

^d^ n.p. = not performed.

In general, for the preparation of compound **2** (Table 1, entry 1), the use of higher amounts of amines (up to 1.2 equivalents) decreased the yield of the reaction, while traces of products were obtained if an inorganic base was added in the second step (e.g., K_2_CO_3_, Cs_2_CO_3_, K_3_PO_4_), independently on the mechanochemical process conditions used at 30 Hz. This trend was also observed for the preparation of compound **4** (Table 1, entry 3), for which the reactants (the acid and the amine) exhibit electronic properties similar to those employed for compound **2**. However, it is also worth to notice that, in the same mechanochemical processing conditions for both steps using ZrO_2_ as milling media, with the same amine (e.g., *p*‐methyl aniline), the methyl *ortho*‐substituted carboxylic acid delivered a slightly better yield (98%) than the corresponding *para*‐substituted substrate (90%), suggesting that, differently than in solution, steric hindrance is not detrimental when mechanochemical conditions are used [25].

The optimization of the milling time in step 1 was also necessary. Indeed, in the preparation of compound **2**, yields dropped to *ca*. 65% (for both SS, stainless steel and ZrO_2_ grinding media) by extending the reaction time in *step 1* from 30 to 60 min, probably due to degradation of the *N*‐acyl derivative **1,** as a consequence of the heating effects at both microscopic and/or macroscopic levels, resulting from friction and impacts, that cannot be ruled out, leading to an increase in bulk temperature. This hypothesis could be confirmed by repeating the reaction in a polytetrafluoroethylene (PTFE) milling jar (Table 1, entry 1 and Supporting Information), delivering a comparable high yield (96%, at 50 Hz), even though the reaction time for step 1 was reduced to 20 min. Cycled milling was also investigated to possibly reduce the heating effects due to continuous milling, however, no benefits were observed.

In addition to mechanochemical processing conditions, the optimization of the reactions needed to account also for the electronic properties of the amine added in the second step of the reaction. For example, in the preparation of compound **3,** involving a poorly nucleophilic amine (*p*‐chloro aniline), the degradation of the *N*‐acyl derivative **1** could be more important due to the slower kinetics of addition of the amine, delivering yields generally lower compared to compound **2** (Table 1, entries 1 vs 2), especially when SS was used as grinding media. These considerations are in coherence also with the reactivity trend observed for the set of compounds **5**, **6**, and **10** (Table 1, entries 4, 5, and 9 respectively), generating the same *N*‐acyl imidazole intermediate **1** from 2‐trifluoromethyl benzoic acid, allowing to isolate the effect of the amines used in the second step.

If steric effects seemed not to be responsible for the outcome of the reactions, electronic effects on both the starting carboxylic acid and the amine were important. This was particularly evident by comparing three sets of compounds: *set 1* (**2**, **4**, **5**, and **7**), *set 2* (**3**, **6**, and **8**), and *set 3* (**9** and **10**). For each set, the amine selected for the second step did not vary (*p*‐methyl aniline, *p*‐chloro aniline, or *m*‐isopropyloxy aniline, respectively), allowing to isolate the effect of the electronic properties of different carboxylic acids. As a general trend, and as expected, the carboxylic acids bearing fluorinated substituents delivered lower yields of the final amides, regardless of the milling conditions used. Indeed, the conversion of the fluorinated carboxylic acids to the corresponding *N*‐acyl derivative **1** was never complete, as indicated by TLC and qualitative GC‐MS analyses of the crude after the first step, showing the presence of the starting (unreacted) carboxylic acid. Worst results were obtained when a poorly nucleophilic amine was used for step 2, as for compound **6** (Table 1, entry 5). At 30 *Hz*, by neat milling or in liquid assisted grinding (LAG) conditions using *N*‐methyl imidazole (NMI), in different processing mechanochemical conditions, the reactions failed. An acceptable 60% yield could be obtained at higher milling frequency (50 *Hz*) in PTFE/ZrO_2_ milling system, extending the reaction time for both steps, to 90 and 180 min respectively. Worth noting is the preparation of compound **5** in comparison with compound **6** (Table 1, entries 4 vs 5, respectively), the only difference being the nucleophilicity of (solid) anilines used.

Even though, for both compounds, the presence of a trifluoromethyl group on the starting carboxylic acid was not optimal for the formation of the corresponding activated *N‐*acyl intermediate **1**, the reaction could occur when the more nucleophilic (and low melting, m.p. 45°C) *p*‐toluidine was used (entry 4) instead of *p*‐chloro aniline (m.p. 72°C). In this case, SS or ZrO_2_ grinding media were also suitable, and the reaction times were shorter for both steps.

However, if the combination of a deactivated carboxylic acid with a deactivated amine was unsuccessful in promoting the reactions in both *step 1* (formation of the *N*‐acyl derivative **1**) and *step 2* (amidation) for compound **6** (entry 5), the same trend was not observed in the preparation of compound **8** (entry 7). In this case, despite the unfavorable electronic properties of the substrates (the acid and the amine) the reaction was really satisfying in different milling conditions (entry 7, and Supporting Information). This result can be explained evoking a cooperative effect due to the presence of the *N‐*methyl pyrazole moiety mitigating, by inductive effects, the strong electron‐withdrawing character of the trifluoro methyl substituent. Along the same line, and by comparing compounds **5** and **7** (entries 4 and 6, respectively), higher yields were obtained when 1‐methyl‐3‐(trifluoromethyl)‐1*H*‐pyrazole‐4‐carboxylic acid was used, even though the differences in yields between the targets are less relevant, being the electron‐rich *p‐*toluidine used in the second step.

The mechanochemical CDI‐mediated amidation reaction in a vibrating ball mill using SS as grinding media, was previously reported [44]. For the reaction scope investigated (Figure 1), the addition of an inorganic base (e.g., K_2_CO_3_, Cs_2_CO_3_, K_3_PO_4_) was tested, but it was detrimental in all the reaction and mechanochemical processing conditions explored, leading to low yields or to the inhibition of the reaction. For all the targets **2–11**, SS was not the best option except for compound **7** (entry 6). This deviation can be attributed to the specific rheology of the reaction, the crude being a viscous paste.

Therefore, the exploration of other mechanochemical processing conditions was a must, with ZrO_2_ grinding media being generally more appropriate for the CDI‐mediated amidation in the ball‐mill, delivering higher average yields, even for the less reactive combination of reagents (in both steps).

The CDI‐mediated amidation reaction in both horizontal and vertical vibrating ball‐mills lacked general applicability for our reaction scope and *ad hoc* reaction conditions should be developed for each target, requiring a continuous remodulation of the mechanochemical parameters, depending on the target to prepare. In particular, the method had limited applicability when deactivated substrates (e.g., fluorinated carboxylic acids and/or poorly nucleophilic amines) were used, delivering modest yields for preparing the marketed agrochemicals Mepronil (**9**), Flutolanil (**10**) and Fluxapyroxad (**11**) (Table 1, entries 8–10). As a matter of fact, in that series, Mepronil (**9**) delivered the highest yield (70%). This is not surprising, not only because of the favorable electronic properties for both the *ortho‐*toluic acid and the 3‐isopropoxy aniline, but also due to the physical state of the amine (a liquid) and the reaction intermediate. Indeed, after the first step, the *N*‐acyl derivative **1** was a viscous liquid, and the mixing with the amine was facilitated being the 3‐isopropoxy aniline also a liquid.

For all the compounds prepared, the formation of the corresponding symmetrical ureas, a typical byproduct in CDI‐mediated reactions, was never observed, as shown by GC‐MS analyses of the crude reaction mixture (just after milling).

With the purpose of preparing the marketed agrochemicals Mepronil (**9**), Flutolanil (**10**), and Fluxapyroxad (**11**) with high productivity and on gram scale, the CDI‐mediated amidation reaction for these selected targets was implemented in a planetary ball‐mill (herein referred to as P7P), using zirconia as grinding media (Table 2).

**TABLE 2:** CDI‐mediated mechanochemical syntheses of marketed agrochemicals **9–11** in a planetary mill (P7P):^a^ energetic considerations for selected experiments, as function of the process parameters for step 1 and yields (after step 2).

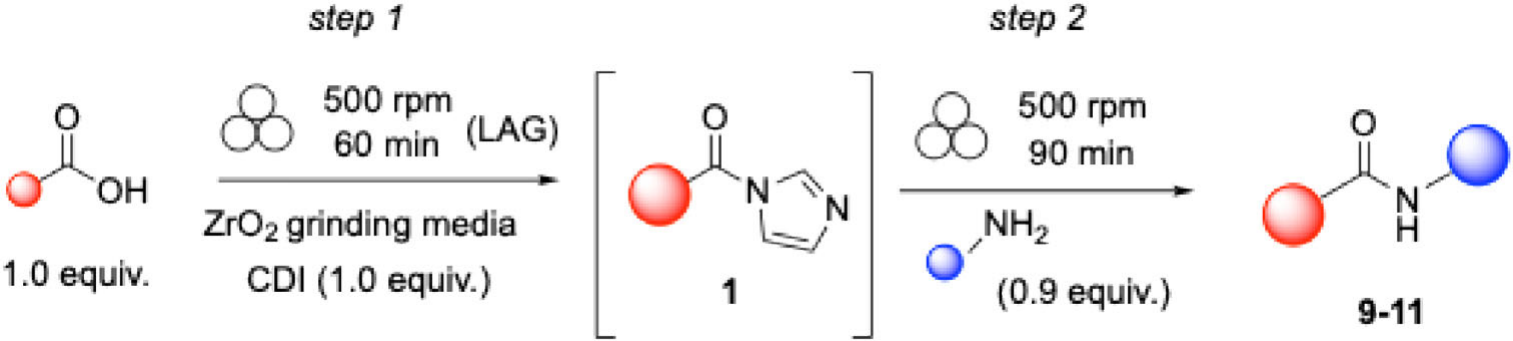					
Entry	Compound	Number of ZrO_2_ balls / mm Ø	E_impact_ (mJ)	E_tot_ (kJ)	Yield (%)
1	9 Mepronil	25 / 5	11.3	31.1	3
2		50 / 5	11.3	62.8	73
3		648^b^ / 3	2.8	149.6	60
4	10 Flutolanil	25 / 5	11.3	31.1	0
5		50 / 5	11.3	62.8	52
6		648^b^ / 3	2.8	149.6	37
7	11 Fluxapyroxad	50 / 5	11.3	62.8	3
8		648^b^ / 3	2.8	149.6	6
9		648^b^ / 3	2.8	149.6	**23** ^c^

^a^ The filling degree was kept constant (*ca*. 60%) in all the experiments. The reaction scale was: 2.7 mmol (entries 1 and 4) in a 20 mL ZrO_2_ jar or 5.4 mmol (entries 2, 3, and 5–9) in a 45 mL ZrO_2_ jar. Typical procedure in planetary ball‐mill at 500 rpm: (*step 1*) acid (1.0 equiv.) and 1,1′‐carbonyldiimidazole (1.0 equiv.) for 60 min; (*step 2*) amine (0.9 equiv.) for 90 min. (*c.f*., the detailed mechanochemical process conditions are reported in the Supporting Information);

^b^ Equivalent to a total mass of 57.1 g of balls.

^c^ *N*‐methylimidazole (NMI) was used in a LAG process (517.6 *µ*L, *η* = 0.16 µL/mg).

For each set of mechanochemical processing parameters investigated (Table 2), a publicly accessible computational tool [58] was employed to quantify the kinetic energy imparted by the milling media to the reactants upon impact. The analysis differentiated between the cumulative kinetic energy transferred to the reaction mixture over the entire milling duration (E_total_, in kJ) and the energy associated with a single ball‐molecule collision event (E_impact_, in mJ). The total mass of the milling balls (total *m*_balls_) was also incorporated into the calculations, given its direct contribution to the magnitude of E_total_ via momentum transfer and impact frequency.

Therefore, for the agrochemical targets **9–11**, the analysis was conducted considering the mechanochemical processing conditions for *step 1* only, accounting for the formation of the activated *N‐*acyl derivative **1** (Table 2). From the extent of success of the first step, it depends essentially the yield of the entire process. The higher the conversion of the starting acid is, the better the yield will be, especially in the case of Mepronil (**9**) and Flutolanil (**10**), having in common 3‐isopropyl aniline added in the second step, providing electron‐donating effect by induction.

In the mechanochemical preparation of Mepronil (**9**), a 73% yield was obtained by milling the reactants at 500 rpm in a ZrO_2_ jar (45 mL, 5.4 mmol scale) with 50×5 mm balls of the same material (Table 2, entry 2, and Supporting Information). However, when the experiment was repeated by replacing the 50×5 mm balls (entry 2) with 25×5 mm balls (entry 1), in a 20 mL ZrO_2_ jar (2.7 mmol scale), the yield dropped to 3%. Even though the energy delivered to the reactants during each single impact (*E*_impact_ = 11.3 mJ) was identical for both experiments (entries 1 and 2), the lower cumulative total energy (E_total_ = 31.1 kJ) could have slowed down the transformation rate into the corresponding *N*‐acyl derivative **1**.

Along the same line, at higher cumulative total energy (E_total_ = 149.6 kJ) and in reaction conditions set to provide higher stress frequency (S_F_) [62, 63] enabling more impacts per unit of time (entry 3), with a 2.4‐fold total cumulative energy (E_total_ = 149.6 kJ vs 62.8 kJ in entry 2), the yield was moderate (60%). Indeed, the energy delivered to the reactants during each single impact (*E*_impact_ = 2.8 mJ vs 11.3 mJ in entry 2) was four times lower, with a consequent less efficient conversion of *ortho*‐toluic acid into the activated intermediate **1**, delivering a lower yield after the second step (60%, entry 3).

This result confirms that greater energy inputs do not consistently yield higher conversion or yields, emphasizing the need for precise optimization of milling parameters rather than reliance on energy intensity alone in the design of efficient mechanochemical protocols. Indeed, the optimization of a mechanochemical reaction is multifactorial and the reactivity of the system needs fine tuning of several variables, accounting also for the rheological behaviors of the mixture and the chemical reactivity of each chemical entity.

This statement finds further confirmation when comparing the outputs for the preparation of Mepronil (**9**) and Flutolanil (**10**), under identical mechanochemical processing conditions (e.g., Table 2, entries 2 vs 5, and 3 vs 6). Even though both values for *E*_impact_ and E_total_ were identical, yields were quite different, accounting for differences in the electronic properties of the starting acids (*o*‐CH_3_‐benzoic acid and *o*‐CF_3_‐benzoic acid, respectively).

This is particularly evident in the case of the mechanochemical preparation of Fluxapyroxad (**11**) (Table 2, entries 7–8), when prepared in identical energetic and processing conditions, already successful for targets **9** and **10** (entries 2 and 5).

The mechanochemical synthesis of Fluxapyroxad (**11**) (entry 7) was in this case not productive. Several different attempts by neat milling were fully unsuccessful (0% yield) with no conversion of the starting materials, due to the highly deactivated nature of both reactants, presenting several electron‐withdrawing fluorine atoms on both the acid (involved in the first step) and the amine (involved in the second one). Moreover, the mechanochemical processing conditions outperforming for Mepronil (**9**) and Flutolanil (**10**) (Table 2, entries 2 and 5) were not effective for the CDI‐mediated synthesis of Fluxapyroxad (**11**) in P7P ball‐mill, delivering only 3% yield (Table 2, entry 7). In the optimal mechanochemical processing conditions (accounting for the simultaneous adjustment of chemical, technical and process parameters), an acceptable yield of 23% (Table 2, entry 9) could be reached only upon the addition of *N‐*methylimidazole (NMI, *η* = 0.16 µL/mg, 1.1 equiv.) in the first step of the mechanochemical synthesis. Indeed, as observed already in solution, carbamoyl imidazole intermediates (**1**, Scheme 1) lack adequate reactivity toward nucleophilic attack, necessitating activation through alkylation of the distal nitrogen of the imidazole nucleus [64].

**SCHEME 2:**
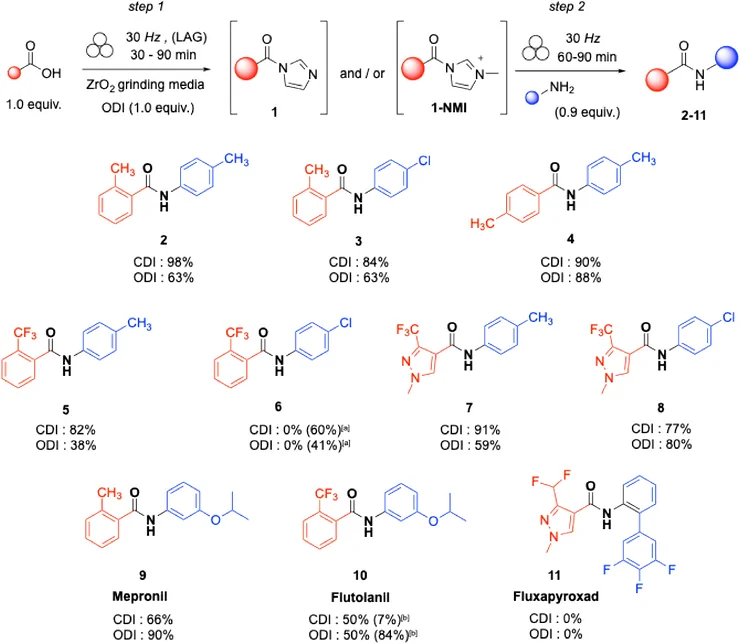
Reaction scope for the ODI‐mediated mechanochemical syntheses of amides **2–11** in a horizontal vibrating ball‐mill (*c.f*., the reaction scale was 1.35 mmol; the detailed mechanochemical process conditions are reported in the Supporting Information and Table S2). The comparative results for the CDI‐mediated amidation reaction in ZrO_2_ grinding media (Table 1) are also indicated. Data in parenthesis refer to the reaction performed at 50 *Hz* for CDI and 30 *Hz* for ODI, with grinding media: (a) PTFE/ZrO_2_ or (b) stainless steel (SS). (*c.f*., the detailed mechanochemical process conditions are reported in the Supporting Information).

The key role played by NMI could be due to several effects and reactions, occurring in parallel or simultaneously. Thus, NMI can act as: i) LAG solvent, ii) base generating, in the first step, a more reactive carboxylate, also due to donor mesomeric effects of the pyrazole ring, and iii) competitor with imidazole, in the formation of a more activated, formally charged, *N‐*acyl methyl imidazolium intermediate **1‐NMI** (Schemes 1 and 2), facilitating the reaction with the severely deactivated amine. Additionally, being NMI a liquid, its contribution from a rheological standpoint to a better mix of the reaction crude cannot be excluded.

However, although the reaction was somehow effective in the presence of NMI, when K_2_CO_3_ was employed instead, the reaction was unsuccessful when fluorinated/deactivated substrates were used, indirectly confirming the pivotal role played by NMI for the success of this specific transformation.

Although the CDI‐mediated amidation leading to the marketed agrochemicals **9–11** was not satisfactory when vibrating ball‐mills were used (Table 1), their gram‐scale preparation was possible in planetary ball‐mills (Table 2), milling in neat (entries 2 and 5, for mepronil **9** and Flutolanil **10**, respectively) or LAG conditions (entry 9, for Fluxapyroxad **11**).

### 2.1 Kinetic Investigations

Kinetic investigations [65] were also conducted to extract and interpret the kinetic curves obtained from real‐time temperature, T, and pressure measurements during the mechanochemical *one‐pot*/two‐step CDI‐mediated amidation leading to Mepronil **9**, Flutolanil **10**, and Fluxapyroxad **11**. Since only the first step shows an appreciable pressure rise associated with CO_2_ formation, the analysis was focused on this stage.

To accurately interpret the pressure profile, it is first necessary to understand how the application of mechanical forces affects gas evolution during milling, where energy is transferred to the material through impulsive collisions. This energy input leads to both mechanical activation and localized heating arising from plastic deformation and friction. Consequently, the observed pressure increase, results from a combination of thermal expansion of pre‐existing gas and the formation of CO_2_ generated by the reaction. Therefore, to obtain reliable kinetic curves describing the amount of CO_2_ produced, these two contributions must be properly disentangled during the data analysis. Therefore, to obtain reliable kinetic curves describing the amount of CO_2_ produced, these two contributions must be properly disentangled during the data analysis, by extracting the kinetic curves from pressure data, and analyzing them by kinetic modelling (*c.f*., Section S3 in the Supporting Information).

Real‐time pressure measurements tracked CO_2_ evolution during CDI‐mediated activation (step 1), serving as a kinetic marker for *N*‐acyl imidazole formation (Figures S9,S10, Supporting Information). Control experiments conducted by ball‐milling CDI alone, confirmed negligible CDI decomposition, attributing pressure increases solely to reaction‐derived CO_2_ (Figure S8, Supporting Information). Kinetic profiles showed sigmoidal shapes with substrate‐dependent rates: Fluxapyroxad (**11**) reacted fastest, followed by Flutolanil (**10**) and Mepronil (**9**) (Figure S9, Supporting Information).

The early‐stage of the reaction appears to be driven by mechanical input, transitioning later to reaction‐driven regimes. This is particularly evident for Flutolanil (**10**), where discontinuous milling confirmed the primary role of initial mechanical activation (Figures S9,S10 of Supporting Information). The statistical kinetic model (Equations 8–12, Supporting Information) allows to rationalize the global kinetics from critical loading conditions (CLCs), extracting key parameters such as interface generation rate (r), reaction probability (α), and powder activation fraction (k). By fitting early‐stage data revealed that the reaction kinetics are substrate (acid)‐dependent, with decreasing kinetics in the order Fluxapyroxad (**11**) > Flutolanil (**10**) > Mepronil (**9**) for both k and α, while r remained almost constant, confirming that chemical reactivity dominates after activated surfaces are formed (Figures S11,S12 of Supporting Information).

This analysis quantitatively benchmarks the limited CDI reactivity upon mechanical stress for deactivated substrates (acids), justifying the use of ODI‐mediated activation for enhanced reactivity. A more comprehensive discussion on the kinetic investigation, including the explanation of the kinetic model is presented in the Supporting Information (*c.f*., Section S3).

### 2.2 1,1′‐Oxalyldiimidazole (ODI)‐Mediated Activation to Amides

Even though CDI was a valid coupling agent for amidation reactions in the vibrating ball‐mill (Table 1), with the best average outputs obtained by using hard grinding media (mainly ZrO_2_), the method resulted in low to moderate reactivity in the presence of deactivated acids (Table 1), even when more energetic mechanochemical processing conditions in a planetary ball‐mill were applied (Table 2, entries 4–9). Therefore, in the quest of a more reactive coupling agent, 1,1’‐oxalyldiimidazole (ODI) was used as an alternative acylating agent to CDI, to prepare the target agrochemicals **9**–**11**, at multigram scale (5.4 mmol). The comparison of ODI with CDI is particularly relevant since both are imidazole‐based coupling agents and work via the formation of the same ***N‐*** acyl imidazole intermediate. However, they differ in reactivity, selectivity, and applications (Table S3, Supporting Information).

However, the use of ODI also requires consideration of carbon monoxide evolution during the activation step. In the present mechanochemical set‐up, however, gas formation takes place inside a sealed milling jar, so the evolved gas remains confined to the reactor and can be managed by appropriate and standard laboratory safety precautions and engineering controls to fully mitigate operator risks. In particular, the use of milling jars, commercially available or in‐house made, equipped with gas‐handling valves provide a practical means to channel the gas in a controlled manner, for venting, for gas‐solid reactions or for subsequent collection or recycling [66, 67, 68]. CO gas itself is industrially employed, demonstrating its viability, also in the green synthesis of Active Pharmaceutical Ingredient [69, 70]. Despite the concerns that can be raised on the evolution of CO gas during the synthesis, solid ODI exhibits lower acute dermal/oral toxicity and lacks inhalation hazards, unlike its liquid counterpart oxalyl chloride (ClCOCOCl), often used in solution for similar purposes, at both laboratory scale [71, 72] and in pharmaceutical manufacturing [73, 74, 75] (e.g., for acid chloride formation) or materials synthesis [76]. By the time of this contribution, to the best of our knowledge, no ECHA REACH (European Chemical Agency Registration, Evaluation, Authorization and Restriction of Chemicals) regulations prohibit both oxalyl dihalides (including oxalyl chloride) or ODI at laboratory, research, or industrial scales. ODI was therefore selected for activating carboxylic acids otherwise not efficiently converted when CDI was used, while preserving the practical advantages of the mechanochemical processing, including solvent‐free processing and simplified workup. In this context, CO evolution should be viewed as a process parameter to be managed by suitable reactor design and operational control, rather than as a factor that negates the synthetic value of the ODI‐mediated approach.

Recommended to be used in dry conditions for best performance (though more tolerant than some others), no special precautions or sophisticated experimental milling set‐up were needed to promote the ODI‐mediated synthesis of amides in a vibrating ball‐mill, and a similar one‐pot/two‐step approach as for the CDI‐mediated amidation described so far (Scheme 2).

In the preparation of targets **2** and **3** sharing *o*‐toluic acid (melting at 102°C–104°C) as starting material and generating the same *N‐*acyl derivative **1** and imidazole (melting at 90°C–91°C) as by‐product, CDI unexpectedly overperformed over ODI coupling agent, and independently on the electronic nature of the amines (m.p. 44°C–45°C for *p*‐toluidine and 71°C for *p*‐chloroaniline, respectively) added in the second step. However, even though the entropic pool is higher for the ODI‐mediated reaction, both the rheology of the reaction mixtures and the melting points of the activating agents (m.p. for CDI 113°C–115°C while ODI decomposes directly at 87°C–92°C [51]), also need to be accounted to explain the reactivity trends observed.

Indeed, in the same milling conditions, the crude mixture after the first step resulted in a dense and compact paste when CDI was used, while a clear liquid was observed for ODI. Even though some decomposition of the more reactive ODI could not be excluded during milling, the transmission of the energy during the impacts could have been more effective when CDI was used, possibly less affected by elastic dispersion than for the reactions making use of ODI.

Along the same line, and differently than CDI‐mediated reactions, in the case of ODI, the addition of liquid NMI as LAG solvent (*η* = 0.16 µL/mg) in the first step proved to be detrimental, switching off the reactions (0% yield for both compounds **2** and **3**). Except for target **6**, for which the use of NMI was beneficial, in all the other cases, and independently on the mechanochemical processing conditions used, yields were halved compared to the values obtained in neat grinding conditions.

Generally speaking, the reactivity trend for the ODI‐mediated amidation reaction in the vibrating ball‐mill was very similar to the corresponding processes involving CDI, with an important drop in yields observed when deactivated acids were used (compounds **2**, **3**, and **9** vs **5**, **6**, and **10**, respectively, Scheme 2).

However, for compound **6** and independently on the activating agent used (CDI or ODI), the output of the reaction strongly depended on the mechanochemical processing parameters used. Indeed, PTFE/ZrO_2_ grinding media outperformed ZrO_2_, even though the CDI‐mediated process required higher milling frequency (50 Hz) and longer reaction time in step 1 (90 min) compared to ODI‐mediated synthesis (at 30 Hz during 60 min for step 1). Along the same line, SS grinding media proved to be more suitable than ZrO_2_ for the preparation of Flutolanil **10** (84% vs 49% yield respectively, Scheme 2), confirming the importance of the fine tuning of the mechanochemical processing parameters, in addition to the chemical reactivity of a specific substrate or reactant.

Despite the several attempts to disclose a general mechanochemical method, the ODI‐mediated amidation reaction in the vibrating ball‐mill only outperformed CDI in the preparation of the fungicides Mepronil (**9**) and Flutolanil (**10**), and the optimization plan remained extremely cumbersome for each of the substrates. The best results are reported in Scheme 2. Furthermore, similarly to the CDI‐mediated amidation reaction in the vibrating ball‐mill, the preparation of Fluxapyroxad (**11**) was unsuccessful under all the (neat or LAG) mechanochemical processing conditions tested.

Our previous findings showed that the CDI‐mediated amidation reactions delivered acceptable (but still moderate) yields of Mepronil (**9**), Flutolanil (**10**) and Fluxapyroxad (**11**) (Table 2, entries 2, 5, and 9, respectively), in a planetary ball‐mill, in neat or LAG conditions, when other (mechano)chemical processing conditions failed. Therefore, the gram‐scale synthesis of these specific targets (**9–11**) was implemented using two different mechanochemical devices, referred to as P6 and P7P and the results were compared for both CDI‐ and ODI‐mediated synthesis, in the same optimized processing conditions (Table 3).

**TABLE 3:** Mechanochemical syntheses of marketed agrochemicals **9–11** in planetary ball mills:^a^ comparative assessment as function of the chemical (CDI vs ODI‐mediated methods) and technical process parameters.

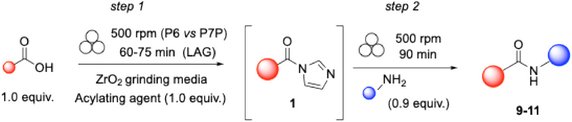					
		Yield (%)			
		CDI^b^		ODI^b^	
Entry	Compound	P6	P7P^c^	P6^d^	P7P
1	**9** **Mepronil**	16	73	**93** ^e^	83
2	**10** **Flutolanil**	21	52	**64** ^f^	55
3	**11** **Fluxapyroxad**	1^g^	23^g^	53^f^ ^g^	**66** ^d^ ^g^

^a^ Reaction scale was 5.4 mmol. Typical procedure in planetary ball‐mill at 500 rpm, using 45 mL ZrO_2_ milling jar, (step 1) acid (1.0 equiv.) and acylating agent (CDI or ODI, 1.0 equiv.); (step 2) amine (0.9 equiv.) for the specified times (*c.f*., the detailed mechanochemical process conditions are reported in the Supporting Information and Table S2);

^b^ 50 ZrO_2_ balls (5 mm ∅, total weight: 19.2 g) were used except when otherwise specified; for step 1: 60 min, for step 2: 90 min;

^c^ Data reported already in Table 2, entries 2, 5 and 9;

^d^ 57.1 g of 3 mm ∅ balls were used;

^e^ The reaction time was: for step 1: 75 min, for step 2: 90 min.;

^f^ The reaction time was 90 min (for both steps);

^g^ *N*‐methylimidazole (NMI, 1.1 equiv.) was used in a LAG process (*η* = 0.16 mL/mg).

Overall, data in Table 3 clearly indicate that, regardless of the planetary milling device used (P6 or P7P), the multigram scale ODI‐mediated amidation reactions outperformed the corresponding CDI method. In particular, for all compounds **9–11**, ODI conditions were more effective particularly with the P6 planetary ball‐mill, with incremental yields in the range of 43%–86% (Table 3, entries 1–3 – CDI vs ODI in P6 experiments), and to a lesser extent in the P7P, with a maximum yield increase of 33% for Fluxapyroxad (**11**) (Table 3, entry 3 – CDI vs ODI in P7P experiments).

For the set of experiments carried out in the P6 planetary mill, Mepronil (**9**) was the compound displaying the highest yield increase (entry 1, 16% vs 93%) under ODI conditions, probably due to the favorable electronic and rheological properties of both the reactants towards both activation/addition steps. In the case of Flutolanil (**10**), this trend was confirmed even though the yield increase observed for ODI conditions was 43% (entry 2, 21% vs 64%), and lower than for Mepronil (**9**). This is not surprising, considering the deactivated nature of the starting *o*‐trifluoromethyl benzoic acid, also requiring longer reaction times for both steps (90 min each). In the case of Fluxapyroxad (**11**) ODI‐mediated amidation outperformed CDI (entry 3, 1% vs 53% CDI vs ODI, respectively) but LAG conditions using NMI were necessary, with an optimized *η* = 0.16 µL/mg value. While LAG was necessary for the preparation of Fluxapyroxad (**11**) in all the mechanochemical conditions tested (Table 3, entry 3), no improvements were observed when it was used in the synthesis of both Mepronil (**9**) and Flutolanil (**10**). Moreover, a complete reaction inhibition was observed for all the targets (**9–11**) when *η* value was increased up to 0.4 µL/mg.

In general, for both CDI and ODI methods, the reaction in the planetary ball‐mill was facilitated by the presence of a liquid reagent (the amine) in the reaction mixture (Table 3, entries 1 and 2), while the addition of *N*‐methylimidazole (NMI, Table 3, entry 3) in LAG conditions (*η* = 0.16 µL/mg) was necessary for both solid and deactivated reagents, delivering better yields compared to the mechanochemical processing condition in dry media.

This trend was particularly evident in the case of Fluxapyroxad (**11**) especially when P7P was used (Table 3, entry 3) and for which the use of NMI was crucial to achieve acceptable yields. The solid fluorinated amine is particularly deactivated towards nucleophilic attack on the activated *N*‐acyl imidazole intermediate **1** (and/or **1‐NMI**) due to the strong electron‐withdrawing effect of three fluorine atoms on the aryl ring. Indeed, the sets of experiments in P7P for both CDI‐ (6% vs 23%, neat vs LAG respectively) and ODI‐ (traces vs 66%, neat vs LAG, respectively) mediated syntheses, showed that the lowest yields were obtained in the absence of NMI, and independently on the mechanochemical processing conditions explored, and again, with a switch‐off of the reaction (for all the targets, **9–11**) at *η* = 0.4 µL/mg.

In comparative terms, for Fluxapyroxad (**11**), the use of ODI as coupling/activating agent was competitive in comparison with CDI, for P6 and particularly P7P, also suggesting that the more reactive ODI is better suited for deactivated substrates/intermediates. For agrochemicals **9–11**, the preparation using CDI as activating agent performed better in the P7P planetary ball‐mill. However, differences were observed when ODI was used with the P6 planetary ball mill, delivering better results for agrochemicals **9** and **10**, while P7P proved more efficient for preparing Fluxapyroxad (**11**). These results can be explained by considering the relative reactivity of CDI vs ODI. They display similar melting points (119°C vs 114°C–116°C, respectively) and they deliver the same activated *N*‐acyl imidazole intermediate **1** (and/or **1‐NMI**), which allows to rule out the possibility of a more favorable rheology for the reactions involving ODI as a consequence of a macroscopic melt.

CDI and ODI display the same stability at room temperature, upon aging, however, their relative stability under identical mechanical stress can be different. Commercial samples of CDI and ODI were equally stable during 24 h at room temperature, as shown by ATR FT‐IR analyses, sampling at t = 0 , 6, and 24 h. However, differences may arise upon mechanochemical processing. Therefore, the better reactivity of ODI was justified by its more favorable entropic pool compared to CDI, with the release of an additional CO molecule, which should help the reaction to evolve favorably towards the activated *N*‐acyl imidazole intermediate **1**, an aspect that becomes important especially in the case of poorly nucleophilic acids.

Clearly, yields were also dependent on a specific technical parameter, namely the ball‐milling device used. While keeping all other technical parameters identical (e.g., milling media, size and number of milling balls, milling jar volume and shape, filling degree and ball to mass ratio), as well as the reaction scale (5.4 mmol), chemical conditions (neat or LAG), and mechanochemical process parameters (milling speed and reaction time), significant differences were observed.

The two different planetary ball‐milling devices used, referred to P6 and P7P, differ in: (i) the distance between the axis of the sun wheel and the axis of the bowl (referred to as *r* in mm), and (ii) the ratio of rotation between the sun wheel and the bowl (referred to as ^i^relativ), providing different main accelerations (G) resulting in a same sun disc speed (i.e., 500 rpm), (Table S4, Supporting Information).

Therefore, the higher acceleration (19.65 G, 192.77.1 m sec^−2^) provided by the P7P planetary ball‐mill was appropriate for the preparation of the target agrochemicals **9–11** via reactions involving CDI (Table 3, entries 1–3), less reactive than ODI, and for which the P7P was the most suitable planetary ball‐mill. Being the reactivity of CDI lower compared to ODI, it is not surprising that, under identical mechanochemical conditions at the same sun disc speed (e.g., 500 rpm), and for the same substrate, a higher acceleration (energy) was needed to promote the reaction (Table S4, Supporting Information), without degradation of the very reactive *N*‐acyl‐imidazole active specie and/or the CDI itself. The shallower energy requirement for a reactive collision was also modulated by using milling balls of smaller diameter (Table 3, 3 mm balls were used for the ODI‐mediated synthesis in P6 planetary ball‐mill), to ensure a high frequency of collision per unit of time, but still delivering lower kinetic energy at each single impact, thereby also preserving ODI and the very reactive *N*‐acyl‐imidazole active species from degradation, which reflects into overall higher yields, also at gram scale, for the experiments conducted in the P6 planetary ball‐mill.

Therefore, in general, if the CDI activating agent resulted more suitable for experiments conducted in the P7P, the use of the P6 seemed to be more appropriate when the more reactive ODI was used (Table 3, entries 1 and 2), for which less energy is required, having a stronger entropic pull in reacting with the acid. However, in the case of Fluxapyroxad **11** (Table 3, entry 3), the unfavorable electronic properties (i.e., a fluorinated acid and a perfluorinated amine) and the physical state of the reagents (solids) required *ad hoc* process conditions. In particular, and synergistically: i) a higher acceleration (energy) to promote the reaction, with the P7P planetary ball‐mill resulting the most appropriate, independently on the activating agent used (CDI vs ODI) and ii) LAG conditions to form a more reactive *N*‐acyl intermediate (e.g., **1‐NMI**) were needed. Indeed, by neat grinding, no reaction was observed in all cases, for both CDI or ODI‐mediated reactions, when either P6 or P7P planetary mills were used.

When it comes to product recovery, and to keep the generally demonstrated environmental‐friendly approach of mechanochemical reactions [10], the eco‐design has to also concern the downstream processes.

Therefore, in general, if the CDI activating agent resulted more suitable for experiments conducted in the P7P, the use of the P6 seemed to be more appropriate when the more reactive ODI was used (Table 3, entries 1 and 2), for which less energy is required, having a stronger entropic pull in reacting with the acid. However, in the case of Fluxapyroxad **11** (Table 3, entry 3), the unfavorable electronic properties (i.e., a fluorinated acid and a perfluorinated amine) and the physical state of the reagents (solids) required *ad hoc* process conditions. In particular, and synergistically: i) a higher acceleration (energy) to promote the reaction, with the P7P planetary ball‐mill resulting the most appropriate, independently on the activating agent used (CDI vs ODI) and ii) LAG conditions to form a more reactive *N*‐acyl intermediate (e.g., **1‐NMI**) were needed. Indeed, by neat grinding, no reaction was observed in all cases, for both CDI or ODI‐mediated reactions, when either P6 or P7P planetary mills were used.

When it comes to product recovery, and to keep the generally demonstrated environmental‐friendly approach of mechanochemical reactions [10], the eco‐design has to also concern the downstream processes.

**FIGURE 2:**
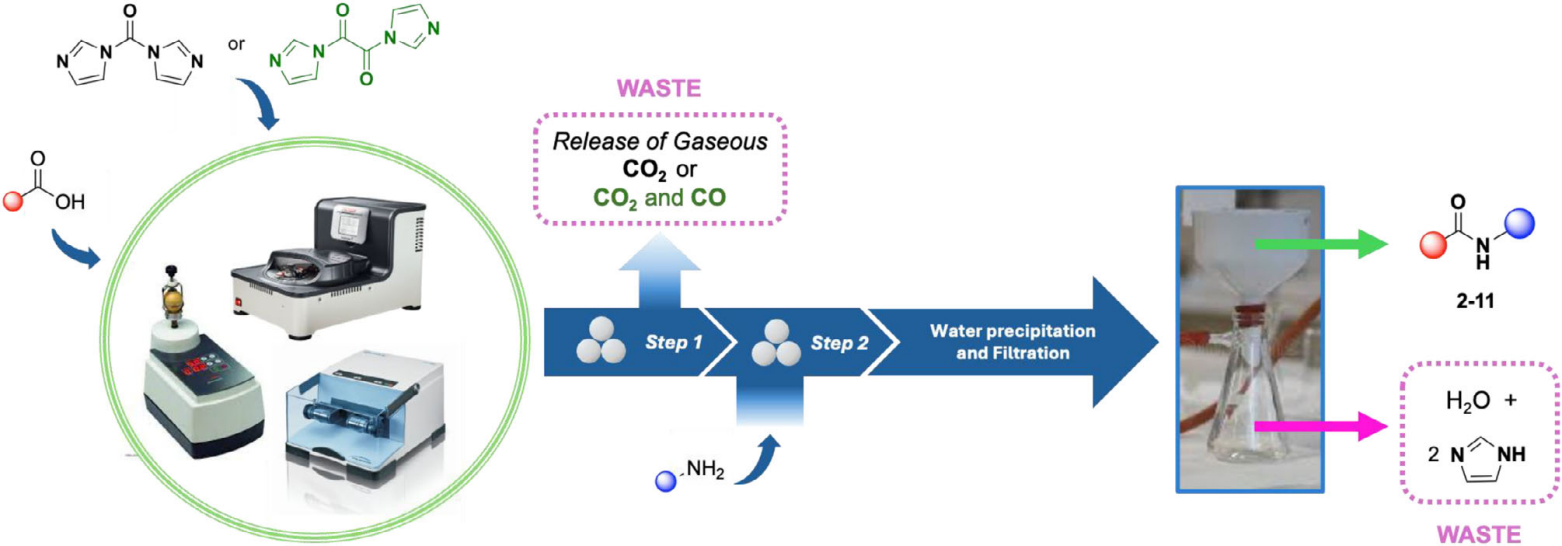
Downstream and eco‐friendly process for recovering the final targets, with no need of organic solvents.

Solvent use during work‐up continues to be one of the most significant challenges that diminishes the advantages of mechanochemical reactions. Therefore, special attention was also devoted to downstream operations, implementing a straightforward recovery of the final targets without the use of organic solvents, by minimizing the use of toxic/hazardous additional chemicals and the amount of waste generated. Therefore, the final targets **2–11** (for both CDI‐ and ODI‐mediated amidation) were universally recovered by precipitation in water, followed by filtration, the only waste being gaseous CO_2_ and CO (from for ODI) in the first step, and the water‐soluble imidazole, generated in the second step (Figure 2).

### 2.3 Comparative Greenness Assessment of Mechanochemical Versus Solution‐Based Processes by Green Chemistry Metrics

The eco‐footprint of the mechanochemical processes was quantitatively assessed by an array of green chemistry metrics calculations [10, 13], using the Chem21 [59], and DOZN^TM^ 3.0 [12, 60, 61, 77] tools (*c.f*., excel files in the Supporting Information). These metrics represent essential tools that should guide the design and optimization of synthetic protocols, particularly when the objective is sustainability and environmental compatibility. The evaluations performed using the DOZN toolkit were paired with calculations with Chem21 to secure an even more objective and quantitative approach to the evaluation of greenness, sustainability and safety of the process. Therefore, green metrics such as atom economy (AE) [78], real mass economy (RME) [79], process mass intensity (PMI) [80], and optimum efficiency (OE) [59] were calculated along with the assessment for solvent and reagent choice, reaction and workup setup, and hazards. The scores obtained with both toolkits, were compared with the corresponding solution‐based preparation methods.

In particular, for the three agrochemicals **9**–**11**, metrics were calculated for both CDI and ODI‐mediated mechanochemical syntheses at gram‐scale in planetary ball‐mills, selecting the methods delivering the best yields (Table 3 and Supporting Information). No CDI‐ or ODI‐mediated synthesis in solution are known for the agrochemicals **9–11**. Therefore, the solution‐based procedures to access Mepronil **9** [15], Flutolanil **10** [16, 17] and Fluxapyroxad **11** [18, 19] were selected from the literature among those that were most closely related to the mechanochemical synthetic methods used herein, to ensure a robust and meaningful comparison. For Fluxapyroxad specifically, the comparison was performed against both a laboratory‐scale synthesis [19] and the industrial method [81], as the former mirrors the activation of acid/amine addition approach, while the latter offers a realistic benchmark against established manufacturing methods.

### 2.4 DOZN Assessment

**FIGURE 3:**
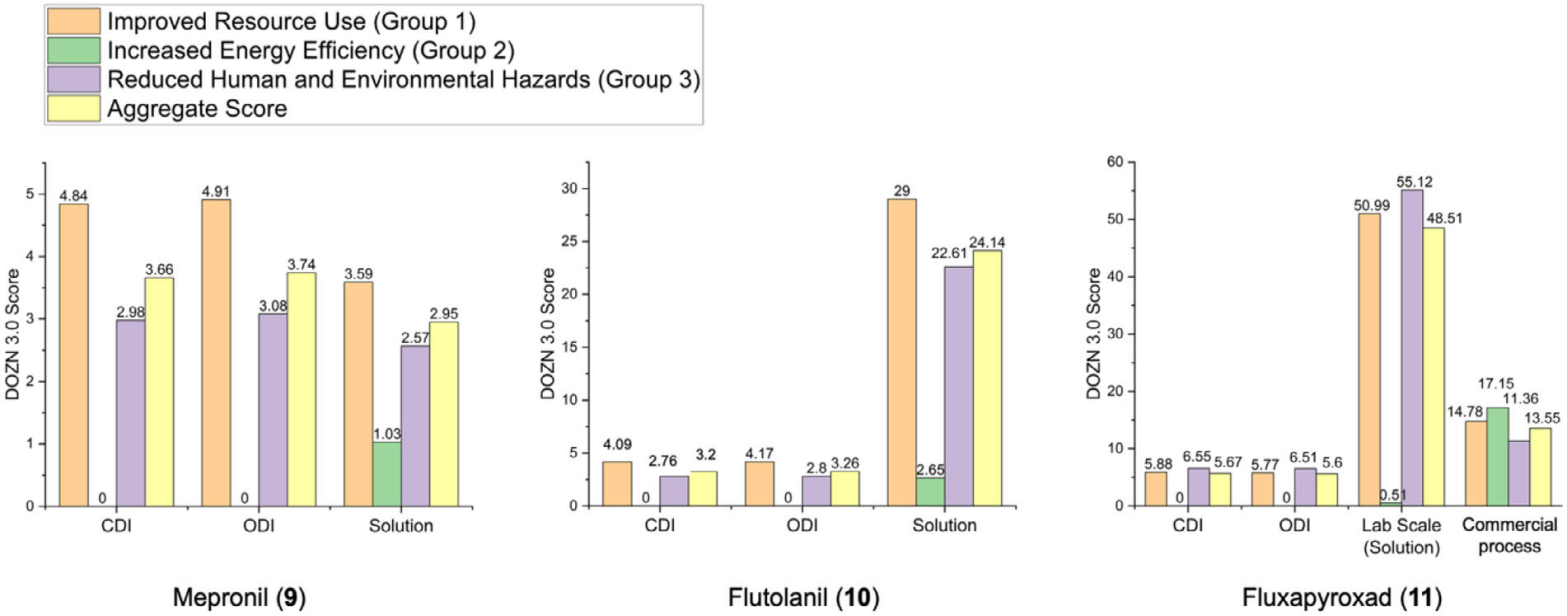
Comparison of solution‐based versus planetary ball mill (PBM) syntheses for both CDI‐ and ODI‐mediated amidation reaction, by DOZN^TM^ 3.0 quantitative scoring tool. Legend: *Group 1* (Improved Resource Use – **orange**), *Group 2* (Increased Energy Efficiency – **green**), *Group 3* (Reduced Human and Environmental Hazards – **violet**), and Aggregate score – **yellow**.

The evaluation of greenness using DOZN^TM^ 3.0 [61] is presented in Figure 3 (and Supporting Information). The tool quantitatively assesses adherence to the twelve principles of green chemistry and has become a valuable approach for evaluating mechanochemical manufacturing processes of commercially relevant molecules in comparison to solution‐based approaches at different scales. DOZN^TM^ tool operates by assigning a score, from 0 to 100 for each of the twelve green principles [60], which are subsequently aggregated into three principle‐based groups reflecting thematic similarities: Improved Resource Use (principles 1, 2, 7, 8, 9, 11 – Group 1), Increased Energy Efficiency (principle 6, Group 2), and Reduced Human and Environmental Hazard (principles 3, 4, 5, 10, 12 – Group 3). An overall *aggregate score* is calculated as the mean of all twelve principle scores. The lower the value, the greener the process, with the optimum *aggregate score* value being below 1.

In the case of Mepronil (**9**), solution‐based synthesis outperformed both CDI‐ or ODI‐ mediated amide bond synthesis by milling, delivering better scores for Groups 1 and 3 (3.59 and 2.57 respectively), as well as for the aggregate (2.95). Performance in this instance is primarily constrained by water consumption relative to product recovery during the work‐up (Figure 2). However, this parameter could be further improved at larger scale, particularly if the solvent (water) is recycled for subsequent workups. The only advantage of the mechanochemical approach was observed for Group 2 (energy efficiency), being the reactions performed at room temperature and atmospheric pressure (instead of heating at 100°C) [15]. Contrarily, for the preparation of Flutolanil (**10**) and Fluxapyroxad (**11**), both involving electron‐withdrawing reagents and substrates, and consistently with the experimental data, both CDI‐ and ODI‐mediated mechanochemical synthesis outperformed the respective solution‐based methods for each group of principles, and with 2.4‐ and 8.6‐fold better aggregate scores for both targets, respectively.

For both Flutolanil (**10**) and Fluxapyroxad (**11**), although lower yields were obtained by both mechanochemical methods (CDI and ODI‐based) compared to solution‐based counterparts, scores for Group 1 and Group 3 resulted better. This is explained mainly by two factors that play a major role in the general economy of the synthetic methods by mechanochemistry. For Group 1 (improved resource use) the better scores resulted not only by the avoidance of solvents (e.g., DMF, CH_2_Cl_2_, Et_2_O), but also in the strategies employed for the activation of the acid and for the isolation of the final product. By mechanochemistry, for both CDI and ODI activating agent, imidazole is generated as by‐product. Being water soluble, it can be washed by water. Conversely, solution‐based routes make use of stoichiometric amount of chlorinating agents to activate the starting carboxylic acid (e.g., oxalyl chloride [16] and PCl_5_ [19] for Flutolanil **10** and Fluxapyroxad **11**, respectively), which produce HCl as a by‐product and necessitate subsequent neutralization with basic additives (i.e., triethylamine or pyridine), directly inflating resource consumption and negatively affecting also Group 3 (reduced human and environmental hazards). Group 3 displayed better scores for the mechanochemical protocols, through systematic elimination of hazardous solvents (DMF, dichloromethane, and diethyl ether) and substitution of air sensitive chlorinating reagents (e.g., PCl_3_ or oxalyl chloride), needing controlled inert gas atmosphere, by safer and air stable imidazole‐based coupling agents (i.e., CDI and ODI). This approach allowed to reduce substantially the occupational health risks and environmental contamination, as their reaction with atmospheric moisture do not generate hazardous acids (e.g., gaseous HCl or phosphoric acid) and exothermic heat. Additionally, the work‐up conducted directly into the milling jar further reduces both acute occupational health risks and environmental contamination, by employing only water or dilute hydrochloric acid, further minimizing exposure to organic solvents and volatile organic compounds. The staggering difference in scores in the case of Fluxapyroxad (**11**) can be further justified by looking at the difference in the workup procedure. The solution‐based protocol needs column chromatography to purify the final compound, extensively dispendious in terms of polluting resources (e.g., silica, petroleum ether mainly), while the mechanochemical counterpart rely only on precipitation in acidified water (Figure 2).

When it comes to Group 2 (increased energy efficiency), the score accounts for any deviation from room standard conditions, which are room temperature and atmospheric pressure. The mechanochemical methods (for both CDI and ODI) operated at room‐temperature and in significantly shorter reaction times compared to their solution‐based counterparts. Indeed, the activation of the acid was the less efficient for each of the targets **9–11**. In the case of Mepronil (**9**), the reaction with POCl_3_ required 3 h at 100°C [15], for Flutolanil (**10**), the reaction by oxalyl chloride needed cooling to 0°C [16], and in the case of the industrial synthesis of Fluxapyroxad (**11**), the reaction occurs in an autoclave under high pressure of CO (9 bar), at high temperatures (130°C) and for a prolonged time (20 h) [81], yielding the worst overall score in this category across the entire assessment.

In general, for all the agrochemicals **9–11**, when comparing CDI vs ODI method by milling, similar scores where always obtained for each group of principles and for the aggregate scores. The minimal variations between the two mechanochemical approaches reflect their inherent similarity in terms of resource consumption patterns, stoichiometry, and reaction conditions, which do not generate sufficient differentiation in the assessment by DOZN^TM^ 3.0.

### 2.5 CHEM21 Assessment

Assessment by CHEM21 [59] (Table 4 and Supporting Information) quantitatively compares methods through green metrics, including the Overall Efficiency (OE), which enables direct comparison across different reaction classes, a feature not consistently achievable with Atom Economy (AE) or Reaction Mass Efficiency (RME). As intrinsic atom‐ or mass‐efficiency varies among transformations, OE provides a more broadly applicable and discriminating metric for assessing synthetic efficiency [59]. Chem21 takes into account also other qualitative criteria that facilitate the assessment of energy efficiency, safety, and potential environmental and health risks. The evaluation framework employs a categorical flag system with clear visual indicators to enhance interpretability and enabling the identification of reaction performance across distinct sustainability dimensions. Each criterion is designated by a color‐coded flag: green denotes “preferred,” amber signifies “acceptable with some concerns,” and red indicates “undesirable.”

**TABLE 4:** Comparison of CHEM21 [60] assessment for solution‐based versus PBM protocol using CDI or ODI as a coupling agents for the synthesis of compounds (**9**), (**10**), and (**11**) (for the full assessment, *c.f*., Supporting Information).

CDI (PBM) versus ODI (PBM) versus Solution																	
	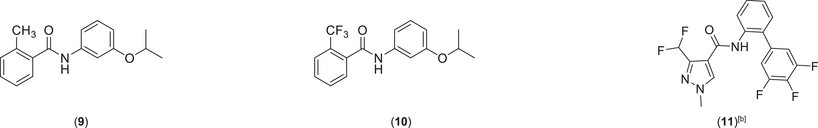																
Metrics^a^	(9)					(10)					(11)^b^						
**AE**	61.4	vs	56.4	vs	80.8	64.2	vs	60.8	vs	69.0	67.9	vs	64.7	vs	62.8	vs	82.5
**RME**	40.5	vs	48.7	vs	61.8	31.2	vs	35.8	vs	54.9	14.6	vs	40.2	vs	50.5	vs	30.3
**OE** ^c^	66.0	vs	86.3	vs	76.5	48.6	vs	58.9	vs	79.6	21.5	vs	62.2	vs	80.4	vs	36.8
**PMI** ^d^	45.1	vs	35.4	vs	13.4	52.4	vs	43.3	vs	55.0	108.9	vs	37.8	vs	742.7	vs	60.4
**PMI_reaction_**	2.5	vs	2.1	vs	6.3	3.2	vs	2.8	vs	50.4	8.0	vs	3.0	vs	28.7	vs	20.9
**PMI_workup_**	42.6	vs	33.3	vs	7.2	49.2	vs	40.5	vs	4.6	100.9	vs	34.9	vs	714.0	vs	39.6
***E*‐factor**	44.1	vs	34.4	vs	12.4	51.4	vs	42.3	vs	54.0	107.9	vs	36.8	vs	741.7	vs	59.4
**Solvent**	No solvent		No solvent		Xylene	No solvent		No solvent		DCM, DMF, THF	NMI (for LAG)		NMI (for LAG)		Et_2_O, THF, AcOEt		ACN
Work‐up, recovery	Water Precipitation, Filtration		Water Precipitation, Filtration		Water washing	Water Precipitation, Filtration		Water Precipitation, Filtration		Crystalliz.	Water Precipitation, Filtration		Water Precipitation, Filtration		Water Washing, Column chrom.		Trituration ( Et_2_O ), Filtration
**Health & safety**																	
**Main hazard statements**	H360d		H331, H220, H360d, H372		H225, H302, H312, H314, H318, H330, H332, H372, H412	H360d		H331, H220, H360d, H372		H220, H225, H260, H280, H302, H304, H312, H314, H315, H319, H331, H332, H335, H336, H341, H350, H351, H360d, H361, H370, H372, H373, H401, H411	H360d		H331, H220, H360d, H372		H224, H225, H302, H304, H312, H314, H318, H319, H320, H330, H331, H335, H336, H351, H361, H373, H411		H220, H225, H280, H290, H302, H312, H314, H317, H318, H319, H331, H332, H335, H336, H351, H360D, H372, H400, H410, H412

*Legend*: Green flag: Preferred; Amber flag: Acceptable with some concerns; Red Flag: Undesirable.

^a^ Best values for AE, RME and OE is 100%, for PMI is 1 and *E*‐Factor = PMI – 1 [87]. When comparing two processes, the one with the lowest PMI will be greenest

^b^ For Fluxapyroxad (**11**) the four columns are referred respectively to: CDI method in PBM, ODI method in PBM, solution‐based method at laboratory scale, and industrial process

^c^ $OE=\frac{RME}{AE}\times 100$ [60];

^d^ Where precise work‐up chemical quantities were unavailable, estimates were derived as detailed in the Supporting Information to ensure fair comparison.

For the three agrochemical targets **9**–**11**, AE, RME and OE alone might suggest an advantage for methodologies in solution. With the exception of Mepronil (**9**) however, metrics such as PMI and E‐factor point to the opposite direction, accounting quantitatively for the resources used in the whole process, which also include the solvents used in the workup (included in the value of PMI_work‐up_), and not only the yield (Table 4).

The mechanochemical methods, particularly the ODI‐mediated amidation, demonstrated superior performance not only in comparison with solution‐based routes for each of the targets (**9‐11**), but also respect to CDI‐mediated method, with the lowest *E*‐factor and the most favorable PMI values (Table 4). Despite the generally lower yields of the mechanochemical methods compared to solution‐based protocols, the better metrics are explained by the absence of solvents (that constitute 66%–96% of the total reaction mass), and the systematic elimination of basic additives required in the solution protocols to neutralize the HCl (or HBr) generated as by‐products. Especially in the case of Fluxapyroxad (**11**) the laboratory scale amidation in solution results in a PMI of 742.7 and an *E*‐factor of 741.7, over 19‐fold worse than the ODI mechanochemical route (PMI 37.8 and *E*‐factor 36.8). This dramatic gap is due to the use of column chromatography as a purification method, which consumes disproportionate resources. This is quantified by the values of PMI_work‐up_ (34.9 vs 714.0, Table 4) in favor for the mechanochemical route. However, in the industrial process the purification conducted by trituration in Et_2_O, followed by filtration, delivered greatly improved PMI and *E*‐factor values (60.4 and 59.4 respectively). The improvement of the total PMI value is also reflected in PMI_work‐up_ values, which is the value that improves most (Table 4). Nonetheless and although PMI and *E*‐factor metrics for the mechanochemical methods with ODI (at 5.4 mmol scale) are better than those of the industrial process, the comparison is not fully realistic. Indeed, mass‐based metrics need to be integrated with energy‐based and life cycle assessments to determine the sustainable footprint of each process [82], ideally at a similar scale.

As for DOZN^TM^ 3.0, Chem21 also substantiated mechanochemical superiority through systematic elimination of hazardous solvents and reactants. Solution‐based procedures rely on solvents with significant occupational and environmental liabilities. The synthesis of Flutolanil (**10**) requires *N,N*‐dimethylformamide (DMF), already subject to strict regulations for industrial use in the EU [83], and dichloromethane (DCM), which is banned for most commercial and industrial uses and is undergoing a complete phase‐out in USA [84]. At laboratory scale, the synthesis of Fluxapyroxad (**11**) makes use of diethyl ether, characterized by an exceptionally low flash point (−45°C) and low autoignition temperature (160°C), representing a severe fire and explosion hazard, while at industrial scale, toxic and polluting PdCl_2_ [85] is used for the carbonylation reaction using CO gas under high pressure.

By contrast, the CDI/ODI‐mediated mechanochemical protocols operate under metal‐ and solvent‐free conditions (or require only minimal amounts of solvent by LAG process with NMI), with potentially better environmental footprint, also in terms of downstream processing, as the final targets are recovered by precipitation in water, instead of: i) employing *n*‐hexane for crystallization in the case of Flutolanil (**10**), and ii) purification by column chromatography for Fluxapyroxad (**11**). Therefore, the PBM route, skipping the need for wasteful workup procedures, along with the easier experimental setup that avoids the use of hazardous chemicals, shows in the CHEM21 assessment a better overall performance, even when yields are lower than those of the solution‐based methods considered for the assessment.

Overall, with the exception of Mepronil (**9**), in all cases, and independently on the assessment tool used (DOZN^TM^ 3.0 or CHEM21), the mechanochemical methods displayed superiority in terms of overall greenness, in comparison to the solution‐based counterparts, at laboratory scale.

## 3 Conclusion

We developed a mechanochemical method for amide bond formation was disclosed and applied to the preparation of a library, including three marketed agrochemicals, Mepronil (**9**), Flutolanil (**10**), and Fluxapyroxad (**11**) at gram‐scale. Therefore, 1,1’‐oxalyldimidazole (ODI) was used as valid alternative to the green activating agent 1,1’‐carbonyldiimidazole (CDI), not suitable for deactivated acids (e.g., containing fluorinated substituents). The mechanochemical amidation reactions by both CDI and ODI were systematically compared and the reactivity profiles investigated by both vibrating ball‐mill (VBM) and planetary ball‐mill (PBM). Accordingly, different mechanochemical processing conditions, set by adjusting chemical variables (e.g., neat grinding vs LAG processes), technical (e.g., type of ball‐mills, milling media, size and number of milling balls, milling jar volume) and process (e.g., operating frequency, and reaction time) parameters were explored. Kinetic investigation for the CDI‐mediated synthesis was also performed. Kinetic analysis suggests that mechanical activation dominates only the early reaction stages. Kinetic datasets were interpreted based on a kinetic model that allows bridging global and local kinetics. It appears that the fraction of powder effectively processed per unit time as well as the reaction probability depend strongly on the substrate nature following the trend Fluxapyroxad **11** > Flutolanil **10** > Mepronil **9**. These findings indicate that, once molecular contact is established, the local chemical environment plays a decisive role in determining the likelihood of chemical transformation. For the three selected agrochemicals **9–11**, the yields obtained were discussed in terms of total cumulative energy (E*_total_* in kJ) and energy delivered to the reactants during each single impact (*E*_impact_ in mJ). Additionally, the reactivity profiles of both CDI and ODI by PBM were investigated, showing different stability as a function of the mechanochemical stress delivered, modulated by specific planetary mill geometries (e.g., Table 4). The selection of 1,1‐ oxalyl diimidazole (ODI) over 1,1'‐carbonyldiimidazole (CDI) when appropriate, represents a pragmatic and new approach to promote amide synthesis by mechanochemistry [33], especially when reactivity constraints (mostly, unfavorable electronic factors) require more reactive coupling agents, as demonstrated for Flutolanil (**10**) and Fluxapyroxad (**11**).

For both CDI‐ and ODI‐mediated mechanochemical syntheses of marketed molecules of interest for agrochemical industry (**9‐11**), the eco‐footprint was assessed by using two different tools for green chemistry metrics calculations (DOZN^TM^ 3.0 and CHEM21 tools). The results were compared to the most pertinent solution‐based processes. The tools delivered converging outputs in the evaluation of the mechanochemical approaches as a whole, and despite the formation of CO as by product from the ODI‐mediated synthesis [69, 70]. Indeed, the assessment of a method for its sustainability, whatever the method is, cannot focus exclusively only on the evaluation of ‘one specific reagent’ or a specific by product formed, but should consider the process as a whole (i.e., efficiency, productivity, safety, costs, including PMI data, operational simplicity, downstream operations, waste treatment etc.). Specifically, while solution‐based chemistry for the preparation of Mepronil (**9**) already demonstrates acceptable performance and sustainability, in the case of Flutolanil (**10**) and Fluxapyroxad (**11**), both CDI‐ and ODI‐mediated mechanochemical synthesis delivered better green chemistry metrics (despite their lower productivity) than the comparative solution‐based processes, with no organic solvents used, at any stage, from synthesis to recovery of the final products, with waste easily removed in water, confirming once again, that amide synthesis by mechanochemistry constitutes a promising route [33].

## Author Contributions

**Andrea Casagrande**: experimental investigations, formal analysis and green metrics. **Rubén Solorzano‐Rodriguez**: review and editing (green chemistry metrics). **Joris Vezzani**: DSC analyses. **Maria Carta**: writing (section on kinetics), review and editing. **Francesco Delogu**: writing (section on kinetics), review and editing. **Evelina Colacino**: funding acquisition, conceptualization, supervision, writing – original draft, review and editing. **Allan Niidu**: funding acquisition, review and editing. All authors have approved the final version of the manuscript.

## Conflicts of Interest

The authors declare no conflicts of interest.

## Supporting information



The authors have cited additional references within the Supporting Information [15, 16, 17, 19, 65, 87, 88, 89, 90, 91, 92, 93, 94]. The data supporting this article, including ^1^H, ^13^C{^1^H} and ^19^F{^1^H} NMR, FT‐IR (ATR) spectra, DSC analyses for compounds **2–11**, excel spreadsheets for green chemistry metrics calculations by Chem21 and DOZN 3.0 tools data input are available as part of the Supplementary Information.

**Supporting File 1**: chem71190‐sup‐0002‐DataFile.zip.

## Data Availability

The data that support the findings of this study are available in the supplementary material of this article.

## References

[1] J. Magano , “Large‐Scale Amidations in Process Chemistry: Practical Considerations for Reagent Selection and Reaction Execution,” Organic Process Research & Development 26 (2022): 1562–1689.

[2] R. M. de Figueiredo , J.‐S. Suppo , and J.‐M. Campagne , “Nonclassical Routes for Amide Bond Formation,” Chemical Reviews 116 (2016): 12029–12122. 27673596 10.1021/acs.chemrev.6b00237

[3] J. Boström , D. G. Brown , R. J. Young , and G. M. Keserü , “Expanding the Medicinal Chemistry Synthetic Toolbox,” Nat Rev Drug Discovery 17 (2018): 709–727. 30140018 10.1038/nrd.2018.116

[4] J. R. Dunetz , J. Magano , and G. A. Weisenburger , “Large‐Scale Applications of Amide Coupling Reagents for the Synthesis of Pharmaceuticals,” Organic Process Research & Development 20 (2016): 140–177.

[5] B. Luo , Y. Hu , Y. Wu , M. Liu , and K. Fang , “Comprehensive Overview of Pyrazole Amide Derivatives as Novel Pesticides for Crop Protection and Pest Management,” Journal of Agricultural and Food Chemistry 73 (2025): 30582–30600. 41267652 10.1021/acs.jafc.5c09296

[6] C. Lamberth , S. Jeanmart , T. Luksch , and A. Plant , “Current Challenges and Trends in the Discovery of Agrochemicals,” Science 341 (2013): 742–746. 23950530 10.1126/science.1237227

[7] H. C. J. Godfray , J. R. Beddington , I. R. Crute , et al., “Food Security: The Challenge of Feeding 9 Billion People,” Science 327 (2010): 812–818. 20110467 10.1126/science.1185383

[8] M. W. A. Phillips , “Agrochemical Industry Development, Trends in R&D and the Impact of Regulation,” Pest Management Science 76 (2020): 3348–3356. 31858688 10.1002/ps.5728 PMC7540551

[9] Food and Agriculture Organisation of United Nations (FAO) : https://www.fao.org/faostat/en/#data/RP/visualize (accessed February 22, 2026).

[10] N. Fantozzi , J.‐N. Volle , A. Porcheddu , D. Virieux , F. García , and E. Colacino , “Green Metrics in Mechanochemistry,” Chemical Society Reviews 52 (2023): 6680–6714. 37691600 10.1039/d2cs00997h

[11] O. Galant , G. Cerfeda , A. S. McCalmont , et al., “Mechanochemistry Can Reduce Life Cycle Environmental Impacts of Manufacturing Active Pharmaceutical Ingredients,” ACS Sustainable Chemistry & Engineering 10 (2022): 1430–1439.

[12] P. Sharma , C. Vetter , E. Ponnusamy , and E. Colacino , “Assessing the Greenness of Mechanochemical Processes With the DOZN 2.0 Tool,” ACS Sustainable Chemistry & Engineering 10 (2022): 5110–5116. 10.1021/acssuschemeng.2c00519 PMC9044506 35493693

[13] A. Casagrande , A. Niidu , R. Aav , D. Kananovich , and E. Colacino , Encyclopedia of Green Chemistry, First Edition, ed. B. Török , (Elsevier, 2025), 2550–2571.

[14] IUPAC Guiding Principles of Responsible Chemistry https://iupac.org/responsible‐chemistry/(accessed February 22, 2026). 10.1039/d5sc08844e PMC13159559 42125140

[15] I. Chiyomaru , S. Kawada , and K. Takita , “US3937840A,” (1976), https://patents.google.com/patent/US3937840A/en?oq=US3937840A.

[16] R. I. Patel , B. Saxena , and A. Sharma , “General Electron–donor–acceptor Complex Mediated Thioesterification Reaction via Site‐selective C–H Functionalization Using Aryl Sulfonium Salts,” Green Chemistry 26 (2024): 10265–10274.

[17] K. Yabutani , K. Ikeda , S. Hatta , and T. Harada , “US4093743A,” (1977).

[18] As an Example, Fluxapyroxad (Sercadis is a top‐tier Succinate dehydrogenase Inhibitors (SDHI) Fungicide Class With an Estimated Global Market Size of 3.9‐4.0 Billions USD,in 2025, https://www.mordorintelligence.com/industry-reports/sdhi-fungicide-market#:~:text=SDHI%20Fungicide%20Market%20Analysis%20by,%25%20CAGR%20over%202026%2D2031, (accessed February 16, 2026).

[19] Y. Zhang , Z. Chen , J. Nie , F.‐G. Zhang , and J.‐A. Ma , “Development of Cyanopyrazoles as Building Blocks to Fungicide Fluxapyroxad and Analogues,” Journal of Organic Chemistry 84 (2019): 7148–7158. 31058497 10.1021/acs.joc.9b00819

[20] K. J. Ardila‐Fierro and J. G. Hernández , “Sustainability Assessment of Mechanochemistry by Using the Twelve Principles of Green Chemistry,” ChemSusChem 14 (2021): 2145–2162. 33835716 10.1002/cssc.202100478

[21] E. Colacino , V. Isoni , D. Crawford , and F. García , “Upscaling Mechanochemistry: Challenges and Opportunities for Sustainable Industry,” Trends in Chemistry 3 (2021): 335–339.

[22] F. Gomollón‐Bel , “Ten Chemical Innovations That Will Change Our World: IUPAC Identifies Emerging Technologies in Chemistry With Potential to Make Our Planet More Sustainable,” Chemistry International 41 (2019): 12–17.

[23] F. Gomollón‐Bel , “Mechanochemists Want to Shake up Industrial Chemistry,” ACS Central Science 8 (2022): 1474–1476. 36439310 10.1021/acscentsci.2c01273 PMC9686207

[24] F. Cuccu , L. De Luca , F. Delogu , et al., “Mechanochemistry: New Tools to Navigate the Uncharted Territory of ‶Impossible″ Reactions,” Chemsuschem 15 (2022): e202200362. 35867602 10.1002/cssc.202200362 PMC9542358

[25] L. Konnert , F. Lamaty , J. Martinez , and E. Colacino , “Solventless Mechanosynthesis of N‐Protected Amino Esters,” Journal of Organic Chemistry 79 (2014): 4008–4017. 24738762 10.1021/jo500463y

[26] J. G. Hernández and C. Bolm , “Altering Product Selectivity by Mechanochemistry,” Journal of Organic Chemistry 82 (2017): 4007–4019. 28080050 10.1021/acs.joc.6b02887

[27] O. V. Lapshin , E. V. Boldyreva , and V. V. Boldyrev , “Role of Mixing and Milling in Mechanochemical Synthesis (Review),” Russian Journal of Inorganic Chemistry 66 (2021): 433–453.

[28] J. M. Ottino , The Kinematics of Mixing: Stretching, Chaos and Transport, (Cambridge University Press, 1989) ISBN 9780521368780.

[29] S. Mateti , M. Mathesh , Z. Liu , et al., “Mechanochemistry: A Force in Disguise and Conditional Effects Towards Chemical Reactions,” Chemical Communications 57 (2021): 1080–1092. 33438694 10.1039/d0cc06581a

[30] N. Q. Vo , S. Odunuga , P. Bellon , and R. S. Averback , “Forced Chemical Mixing in Immiscible Alloys During Severe Plastic Deformation at Elevated Temperatures,” Acta Materialia 57 (2009): 3012–3019.

[31] E. Juaristi and C. G. Avila‐Ortiz , “Salient Achievements in Synthetic Organic Chemistry Enabled by Mechanochemical Activation,” Synthesis 55 (2023): 2439–2459.

[32] A. A. Michalchuk , I. A. Tumanov , V. A. Drebushchak , and E. V. Boldyreva , “Advances in Elucidating Mechanochemical Complexities via Implementation of a Simple Organic System,” Faraday Discussions 170 (2014): 311–335. 25406486 10.1039/c3fd00150d

[33] T. Nikonovich , C. Chatzigiannis , C. Bordier , et al., Chemical Reviews (2026), in press, 10.1021/acs.chemrev.5c00534. PMC13261792 42159286

[34] H. A. Staab , “Reaktionsfähige Heterocyclische Diamide Der Kohlensäure,” Justus Liebigs Annalen der Chemie 609 (1957): 75–83.

[35] H. A. Staab , “Reaktionsfähige N‐Carbonsäureester und N‐Carbonsäureamide des Imidazols und Triazols,” Justus Liebigs Annalen der Chemie 609 (1957): 83–88.

[36] H. A. Staab , “New Methods of Preparative Organic Chmistry IV. Syntheses Using Heterocyclic Amides (Azolides),” Angewandte Chemie International Edition in English 1 (1962): 351–367.

[37] G. W. Anderson and R. Paul , “N,N'‐Carbonyldiimidazole, A New Reagent for Peptide Synthesis,” Journal of the American Chemical Society 80 (1958): 4423–4423.

[38] J. Gante , “Peptide Syntheses via N‐(1‐Imidazolylcarbonyl)‐ and N‐(1‐Imidazolylthiocarbonyl)‐Amino Esters,” Angewandte Chemie International Edition in English 5 (1966): 315.

[39] R. Paul and G. W. Anderson , “N,N'‐Carbonyldiimidazole, a New Peptide Forming Reagent^1^ ,” Journal of the American Chemical Society 82 (1960): 4596–4600.

[40] A. K. Saha , H. Rapoport , and P. Schultz , “1,1'‐Carbonylbis(3‐methylimidazolium) Triflate: An Efficient Reagent for Aminoacylations,” Journal of the American Chemical Society 111 (1989): 4856–4859.

[41] J.‐S. Suppo , G. Subra , M. Bergès , R. Marcia de Figueiredo , and J.‐M. Campagne , “Inverse Peptide Synthesis via Activated α‐Aminoesters,” Angewandte Chemie International Edition 53 (2014): 5389–5393. 24757099 10.1002/anie.201402147

[42] D. J. Dale , J. Draper , P. J. Dunn , et al., “The Process Development of a Scaleable Route to the PDE5 Inhibitor UK‐357,903,” Organic Process Research & Development 6 (2002): 767–772.

[43] D. J. Dale , P. J. Dunn , C. Golightly , et al., “The Chemical Development of the Commercial Route to Sildenafil: A Case History,” Organic Process Research & Development 4 (2000): 17–22.

[44] T.‐X. Métro , J. Bonnamour , T. Reidon , J. Sarpoulet , J. Martinez , and F. Lamaty , “Mechanosynthesis of Amides in the Total Absence of Organic Solvent From Reaction to Product Recovery,” Chemical Communications 48 (2012): 11781–11783.PMID: 23108313. 23108313 10.1039/c2cc36352f

[45] L. Konnert , M. Dimassi , L. Gonnet , F. Lamaty , J. Martinez , and E. Colacino , “Poly(ethylene) Glycols and Mechanochemistry for the Preparation of Bioactive 3,5‐disubstituted Hydantoins,” RSC Advances 6 (2016): 36978–36986.

[46] M. Lupacchini , A. Mascitti , L. Tonucci , N. d'Alessandro , E. Colacino , and C. Charnay , “From Molecules to Silicon‐Based Biohybrid Materials by Ball Milling,” ACS Sustainable Chemistry & Engineering 6 (2018): 511–518.

[47] L. Konnert , L. Gonnet , I. Halasz , et al., “Mechanochemical Preparation of 3,5‐Disubstituted Hydantoins From Dipeptides and Unsymmetrical Ureas of Amino Acid Derivatives,” Journal of Organic Chemistry 81 (2016): 9802–9809. 27679874 10.1021/acs.joc.6b01832

[48] A. Mascitti , M. Lupacchini , R. Guerra , et al., “Poly(ethylene glycol)s as Grinding Additives in the Mechanochemical Preparation of Highly Functionalized 3,5‐disubstituted Hydantoins,” Beilstein Journal of Organic Chemistry 13 (2017): 19–25. 28179944 10.3762/bjoc.13.3 PMC5238572

[49] A. Porcheddu , F. Delogu , L. De Luca , and E. Colacino , “From Lossen Transposition to Solventless “Medicinal Mechanochemistry”,” ACS Sustainable Chemistry & Engineering 7 (2019): 12044–12051.

[50] M. Lanzillotto , L. Konnert , F. Lamaty , J. Martinez , and E. Colacino , “Mechanochemical 1,1′‐Carbonyldiimidazole‐Mediated Synthesis of Carbamates,” ACS Sustainable Chemistry & Engineering 3 (2015): 2882–2889.

[51] S. Murata , “1,1′‐oxalyldiimidazole, a New Reagent for Activation of Carboxylic Acid,” Chemistry Letters 12 (1983): 1819–1820.

[52] T. Kitagawa , H. Sasaki , and N. Ono , “A Useful Method for the Conversion of Aldehyde Oximes Into Nitriles Using 1,1'‐oxalyldiimidazole,” Chemical and Pharmaceutical Bulletin 33 (1985): 4014–4016.

[53] A. Nefzi , M. A. Giulianotti , and R. A. Houghten , “Solid‐phase Synthesis of Branched Thiohydantoin Benzimidazolinethiones and Branched Thiohydantoin Tetrahydroquinoxalinediones,” Tetrahedron Letters 41 (2000): 2283–2287.

[54] Z. Liu , W. Chen , M. A. Giulianotti , and R. A. Houghten , “Solid‐phase Synthesis of 2,3,4,5‐tetrahydro‐1H‐benzo[e][1,4]Diazepine Derivatives,” Tetrahedron Letters 52 (2011): 4112–4113.

[55] M. Menichincheri , D. Ballinari , A. Bargiotti , et al., “Catecholic Flavonoids Acting as Telomerase Inhibitors,” Journal of Medicinal Chemistry 47 (2004): 6466–6475. 15588081 10.1021/jm040810b

[56] T. C. Stamatatos , S. P. Perlepes , C. P. Raptopoulou , et al., “Initial Use of 1,1′‐oxalyldiimidazole for Inorganic Synthesis: Decomposition of the Ligand as a Means to the Preparation of an Imidazole‐ and Oxalate(‐2)‐containing, 1D Copper(II) Complex,” Inorganic Chemistry Communications 12 (2009): 402–405.

[57] J. H. Lee , J.‐E. R. Rho , T.‐H. D. Rho , and J. G. Newby , “Advent of Innovative Chemiluminescent Enzyme Immunoassay,” Biosensors and Bioelectronics 26 (2010): 377–382. 20739174 10.1016/j.bios.2010.07.126

[58] O. F. Jafter , S. Lee , J. Park , C. Cabanetos , and D. Lungerich , “Navigating Ball Mill Specifications for Theory‐to‐Practice Reproducibility in Mechanochemistry,” Angewandte Chemie International Edition 63 (2024): e202409731, https://lungerich‐group.github.io, The online tool is available under, accessed December 23, 2025. 39148147 10.1002/anie.202409731

[59] C. R. McElroy , A. Constantinou , L. C. Jones , L. Summerton , and J. H. Clark , “Towards a Holistic Approach to Metrics for the 21st Century Pharmaceutical Industry,” Green Chemistry 17 (2015): 3111–3121.

[60] A. DeVierno Kreuder , T. House‐Knight , J. Whitford , et al., “A Method for Assessing Greener Alternatives between Chemical Products Following the 12 Principles of Green Chemistry,” ACS Sustainable Chemistry & Engineering 5 (2017): 2927–2935, DOZN^TM^ is a universal tool, suitable for scoring any process, no matter the activation technique used, once the Detailed Experimental/Process Conditions Are Known. The DOZN^TM^ tool is accessible free of charge here, https://bioinfo.merckgroup.com/dozn (accessed June 27, 2025).

[61] P. Sharma and E. Ponnusamy , “DOZN™3.0: a Quantitative Green Chemistry Evaluator for Sustainable Chemistry,” Sustainable Chemistry for Climate Action 7 (2025): 100129, https://www.sciencedirect.com/science/article/pii/S2772826925000744.

[62] R. Schmidt , C. F. Burmeister , M. Baláž , A. Kwade , and A. Stolle , “Effect of Reaction Parameters on the Synthesis of 5‐Arylidene Barbituric Acid Derivatives in Ball Mills,” Organic Process Research & Development 19 (2015): 427–436.

[63] A. Stolle , R. Schmidt , and K. Jacob , “Scale‐up of Organic Reactions in Ball Mills: Process Intensification With Regard to Energy Efficiency and Economy of Scale,” Faraday Discuss 170 (2014): 267–286. 25406485 10.1039/c3fd00144j

[64] J. A. Grzyb , M. Shen , C. Yoshina‐Ishii , W. Chi , R. S. Brown , and R. A. Batey , “Carbamoylimidazolium and Thiocarbamoylimidazolium Salts: Novel Reagents for the Synthesis of Ureas, Thioureas, Carbamates, Thiocarbamates and Amides,” Tetrahedron 61 (2005): 7153–7175.

[65] L. Vugrin , M. Carta , S. Lukin , E. Meštrović , F. Delogu , and I. Halasz , “Mechanochemical Reaction Kinetics Scales Linearly With Impact Energy,” Faraday Discussions 241 (2023): 217–229. 36149388 10.1039/d2fd00083k

[66] S. Bhatnagar , M. Felderhoff , and J.‐H. Schöbel , “Solvent‐Free Mechanocatalytic Reduction of Nitro Compounds With Hydrogen Gas and Its Application in the Synthesis of Drugs and Their Intermediates,” Advanced Synthesis & Catalysis 367 (2025): e70071.

[67] F. Puccetti , T. Rinesch , S. Suljić , K. Rahimi , A. Herrmann , and C. Bolm , “NMR in Operando Monitoring of Mechanochemically Accelerated Sublimations,” Chem 9 (2023): 1318–1332.

[68] S. Reichle , M. Felderhoff , and F. Schüth , “Mechanocatalytic Room‐Temperature Synthesis of Ammonia From Its Elements Down to Atmospheric Pressure,” Angewandte Chemie International Edition 60 (2021): 26385–26389. 34651400 10.1002/anie.202112095 PMC9299217

[69] For an example on the use of CO in industrial settings : P. T. Anastas and M. M. Kirchhoff , “Origins, Current Status, and Future Challenges of Green Chemistry,” Accounts of Chemical Research 35 (2002): 686–694. 12234198 10.1021/ar010065m

[70] For an example on the use of CO in industrial settings: BHC Company , “BHC Company Ibuprofen Process. In The Presidential Green Chemistry Challenge Awards Program: Summary of 1997 Award Entries and Recipients; EPA744‐S‐97‐001,” (1998): 2, U.S. Environmental Protection Agency, Office of Pollution Prevention and Toxics: Washington, DC.

[71] S. F. E. Hansen and T. Ulven , “Oxalyl Chloride as a Practical Carbon Monoxide Source for Carbonylation Reactions,” Organic Letters 17 (2015): 2832–2835, https://pubs.acs.org/doi/10.1021/acs.orglett.5b01252. 26000869 10.1021/acs.orglett.5b01252

[72] J. Wang , Y. Yin , X. He , et al., “Nickel‐Catalyzed Highly Selective Reductive Carbonylation Using Oxalyl Chloride as the Carbonyl Source,” ACS Catalysis 13 (2023): 8161–8168.

[73] C. A. Rudd Gretton , “Prescription Digital Therapeutics WO20260041667A1,” (2026).

[74] J. R. Murphy , P. S. Bury , N. A. Wagstaff , P. S. Hinchliffe , and S. E. Osbourn , “Salt Forms of Bis(3‐(2‐(Dimethylamino)ethyl)‐1H‐indol‐4‐yl) 3,3′‐Oxydipropionate,” (2025), WO2025238258A1.

[75] C. Feilding‐Mellen and T. Mason , “Pharmaceutical Compositions Comprising 5‐Methoxy‐N,N‐Dimethyltryptamine (5‐MeO‐DMT),” (2025), WO2025068714A1.

[76] E. Jeong , S. Kim , J.‐H. Lim , et al., “Compound, Electrolyte for Rechargeable Lithium Battery Including the Same, and Rechargeable Lithium Battery Including the Same,” (2026), US20260011782A1.

[77] C. M. Chatzigiannis , S. M. Scalzullo , E. Colacino , and M. E. Rivas , “Multigram‐Scale Organic Synthesis by Resonant Acoustic Mixing: Application to the Sustainable Preparation of Active Pharmaceutical Ingredients,” ChemSusChem 18 (2025): e202500110. 40260910 10.1002/cssc.202500110 PMC12232105

[78] B. Trost , “The Atom Economy—A Search for Synthetic Efficiency,” Science 254 (1991): 1471–1477. 1962206 10.1126/science.1962206

[79] M. G. T. C. Ribeiro and A. A. S. C. Machado , “Greenness of Chemical Reactions – limitations of Mass Metrics,” Green Chemistry Letters and Reviews 6 (2013): 1–18.

[80] J. Martínez , J. F. Cortés , and R. Miranda , “Green Chemistry Metrics, A Review,” Processes 10 (2022): 1274.

[81] T. Zierke , J. Rheinheimer , M. Rack , et al., “WO2008145740A1,” (2008).

[82] E. Lucas , A. J. Martín , S. Mitchell , et al., “The Need to Integrate Mass‐ and Energy‐based Metrics With Life Cycle Impacts for Sustainable Chemicals Manufacture,” Green Chemistry 26 (2024): 9300–9309.

[83] Regulation (EU) 2021/2415, L2415/2016 http://data.europa.eu/eli/dir_impl/2021/2415/oj (accessed January 2, 2026).

[84] U.S EPA Regulation: Risk Management for Methylene Chloride Available at https://www.epa.gov/assessing‐and‐managing‐chemicals‐under‐tsca/risk‐management‐methylene‐chloride (Accessed November 19, 2025).

[85] J. Kielhorn , C. Melber , D. Keller , and I. Mangelsdorf , “Palladium – A Review of Exposure and Effects to human Health,” International Journal of Hygiene and Environmental Health 205 (2002): 417–432. 12455264 10.1078/1438-4639-00180

[86] R. A. Sheldon , “The E Factor: Fifteen Years On,” Green Chemistry 9 (2007): 1273.

[87] D. C. Braddock , P. D. Lickiss , B. C. Rowley , et al., “Tetramethyl Orthosilicate (TMOS) as a Reagent for Direct Amidation of Carboxylic Acids,” Organic Letters 20 (2018): 950–953. 29394071 10.1021/acs.orglett.7b03841

[88] B. F. Cain , “2‐Acyloxymethylbenzoic Acids. Novel Amine Protective Functions Providing Amides with the Lability of Esters,” Journal of Organic Chemistry 41 (1976): 2029–2031.

[89] J. Liu , Q. Liu , H. Yi , et al., “Visible‐Light‐Mediated Decarboxylation/Oxidative Amidation of α‐Keto Acids With Amines Under Mild Reaction Conditions Using O_2_ ,” Angewandte Chemie International Edition 53 (2014): 502–506. 24272969 10.1002/anie.201308614

[90] S. Mkrtchyan , M. Jakubczyk , S. Lanka , et al., “Mechanochemical Transformation of CF_3_ Group: Synthesis of Amides and Schiff Bases,” Advanced Synthesis & Catalysis 363 (2021): 5448–5460.

[91] B. Picard , T. Fukuyama , and I. Ryu , “Phosphine‐Free Aminocarbonylation Using Pd/DBU Catalyst: Carbonylative Coupling of Aryl Iodides and Amines,” Journal of Organic Chemistry 88 (2023): 5220–5225. 36525565 10.1021/acs.joc.2c02369

[92] U. Troppmann , W. Mayer , S. Koltzenburg , R. Israels , A. Bauder , and U. Schlotterbeck , “US8735321B2,” (2009).

[93] S.‐P. Wang , C. W. Cheung , and J.‐A. Ma , “Direct Amidation of Carboxylic Acids With Nitroarenes,” Journal of Organic Chemistry 84 (2019): 13922–13934. 31591886 10.1021/acs.joc.9b02068

[94] Y. Wang , B. Tian , Y. Li , et al., “A Sustainable and Versatile Cellulose‐Based CO Surrogate for Carbonylative Reactions,” ChemSusChem 17 (2024): e202301324. 38199959 10.1002/cssc.202301324

