# Supplementary material for: Mechanochemical Amidation Mediated by 1,1’‐Oxalyldiimidazole for the Synthesis of Agrochemicals
Source: Chemistry. 2026 May 29;32(34):e71190. doi: 10.1002/chem.71190 (PMC13569969; doi:10.1002/chem.71190)

SUPPORTING INFORMATION

Mechanochemical amidation using 1,1'-oxalyldiimidazole as a coupling agent for the synthesis of agrochemicals

Andrea Casagrande,[a,b] Rubén Solorzano-Rodriguez,[a] Maria Carta,[c] Francesco Delogu,[c] Joris Vezzani,[a] Allan Niidu,[b] Evelina Colacino.[a]*

[a] A. Casagrande, R. Solorzano-Rodriguez, Joris Vezzani, E. Colacino*
ICGM, Univ. Montpellier, CNRS, ENSCM, Montpellier, France
*E-mail: evelina.colacino@umontpellier.fr

[b] A. Casagrande, A. Niidu
Virumaa College, Tallinn's University of Technology, Ehitajate tee 5, 19086, Tallinn, Estonia

[c] M. Carta, F. Delogu
Department of Mechanical, Chemical and Materials Engineering, University of Cagliari, via Marengo 2, 09123 Cagliari, Italy.

TABLE OF CONTENT

EXPERIMENTAL PART – General Remarks S3

Table S1. Weight for the grinding balls used for the experiments. S4

S1. General experimental procedures S4

S1.1 CDI-mediated synthesis of amides in a horizontal mixer-mill (MM400, Retsch). Method A1 – Compounds 2-5, 7-11 (Table 1). S4

S1.2 CDI-mediated synthesis of amides in a vertical mixer-mill (Pulverisette 23, P23 Fritsch). Method A2. Compounds 2, 3, 5-11 (Table 1). S4

S1.3 CDI-mediated synthesis of amides in a planetary ball-mill (P7 Premium Line, P7P, Fritsch). Method A3. Compounds 9-11 (Table 2, entries 1-9). S5

S1.4 ODI-mediated synthesis of amides in a horizontal vibrating ball mill (MM400, Retsch). Method B1. Compounds 2-4, 6, 8-10 (Scheme 2). S5

S1.5 ODI-mediated synthesis of amides in a planetary ball-mill (P6 or P7 Premium Line, Fritsch). Method B2 - Compounds 9-11 (Table 3). S5

Table S2. Reaction scope and technology assessment sum-up for CDI- and ODI-mediated synthesis of amides. Sum-up of the best experimental conditions for each target. S6

S2. Experimental data and characterizations. S6

2-Methyl-*N*-(4-tolyl)benzamide **(2).** S6

4'-Chloro-toluanilide **(3).** S7

N-(4-methylphenyl)-4-methyl-benzamide **(4).** S8

N-(4-methylphenyl)-2-(trifluoromethyl)benzamide **(5).** S9

N-(4-chlorophenyl)-2-(trifluoromethyl)benzamide **(6).** S10

1-Methyl-*N*-(p-tolyl)-3-(trifluoromethyl)-1H-pyrazole-4-carboxamide **(7).** S11

1-Methyl-*N*-(*p*-chloro)-3-(trifluoromethyl)-1H-pyrazole-4-carboxamide **(8).** S12

2-Methyl-N-(3-propan-2-yloxyphenyl)benzamide (Mepronil) **(9).** S13

N-(3-Isopropoxyphenyl)-2-(trifluoromethyl)benzamide (Flutolanil) **(10).** S15

3-(Difluoromethyl)-1-methyl-*N*-(3',4',5'-trifluoro[1,1'-biphenyl]-2-yl)-1H-pyrazole-4-carboxamide (Fluxapyroxad) **(11).** S16

Table S3. ODI *vs* CDI as Coupling Agents. S17

S3. Kinetic investigations S17

S4. The effect of technical parameter and difference of different models of planetary ball mills in the ODI-mediated synthesis of 9-11. S24

Table S4. Comparison of technical parameters of planetary ball-mills: P6 and P7P at a same sun disc speed (i.e., 500 rpm). S24

S5. Green chemistry metrics S24

References S25

^{1}H, ^{13}C{^{1}H} and ^{19}F{^{1}H} NMR Spectra S26

EXPERIMENTAL PART

General remarks

All reagents were commercially available and used without any further purification.

NMR spectra were recorded at room temperature on a Bruker 400 MHz spectrometer at room temperature. Chemical shifts (δ) of ^{1}H-NMR and ^{13}C{^{1}H}-NMR spectra are reported in ppm relative to residual solvent signals with the appropriate deuterated solvent (DMSO-d_6 and $CDCl_3$). Chemical shifts (*δ*) of ^{1}H NMR and ^{13}C{^{1}H} NMR spectra are reported in ppm relative to residual solvent signals (DMSO in DMSO-d_6: *δ* = 2.52 ppm for ^{1}H and *δ* = 39.52 ppm for ^{13}C{^{1}H} NMR; $CHCl_3$ in $CDCl_3$: δ = 7.27 ppm for 1H and $^{13}CDCl_3$: δ = 77.16 ppm for ^{13}C{^{1}H} NMR); *J* values are given in *Hz*. ^{1}H and ^{13}C{^{1}H} NMR spectra were recorded at 400 MHz and 100.63 MHz respectively. ^{19}F{^{1}H} NMR spectra were recorded at 376 M*Hz* with the same instrument. The chemical shifts are expressed in parts per million (ppm), reported from $CFCl_3$ *δ* = 0.0 ppm. The following abbreviations are used to express the multiplicity: s (singlet), d (doublet), t (triplet), q (quadruplet), quint (quintuplet), dd (doublet of doublet), dt (doublet of triplets), td (triplet of doublets) or m (multiplet). The identity of analytically pure final products was assessed by comparison of the spectral data previously described in the literature and by their fragmentation in GC−MS.

GC–MS analyses were performed in a GC Agilent 6890 (Agilent Technologies, USA) that was fitted with a mass detector Shimadzu GCMS-QP2010 SE, using a 20 m long capillary column, internal diameter (i.d.) of 0.18 mm and film thickness 0.18 µm (Zebron). GC conditions were: injection split 1:20, injector temperature 250 °C, detector temperature 280 °C. Gas carrier: helium (0.74 mL/min), temperature program: from 50 °C (3 min) to 300 °C at 10 °C/min.

HRMS analyses were performed with a Q-TOF mass analyzer (Waters, 2001) with ESI ionization mode [Tolerance = 1.0 mDa / DBE: min = -3.0, max = 100.0 , Element prediction: Off, Number of isotope peaks used for i-FIT = 3, Monoisotopic Mass, Even Electron Ions 1979 formula(e) evaluated with 4 results within limits (up to 50 best isotopic matches for each mass).

Melting points were measured in an open capillary on a Stuart SMP50 automatic melting point apparatus or by Differential Scanning Calorimetry (DSC).

DSC measurements for all compounds were performed with a Netzsch DSC 200F3, at the heating rate of 2 °C min^{-1}. Samples were placed in hermetic aluminum pans.

All the experiments were carried out in duplicate to ensure reproducibility of the experimental data. Yields refer to pure isolated materials.

The Vibrating ball-milling (VBM) experiments were performed in:

a) a horizontal MM400 or MM500 Vario mixer-mill (Retsch GmbH, Haan, Germany) using 5 mL or 10 mL stainless steel jar with 1 stainless steel ball, 10 mm Ø (Table S1) or 10 mL zirconium oxide jar with zirconium oxide ball(s) of 10 mm Ø, 7 mm Ø , 5 mm Ø or 3 mm Ø (Table S1);
b) a vertical mixer-mill Pulverisette 23 (Fritsch GmbH, Idar-Oberstein, Germany) using a 5 mL PTFE jar with 1 zirconium oxide ball of 10 mm Ø – Table S1).

The planetary ball-mill (PBM) experiments were performed in a Pulverisette 6 or Pulverisette 7 Premium (Fritsch GmbH, Idar-Oberstein, Germany) using 12, 20 or 45 mL zirconium oxide jar (with zirconium oxide balls of 5 mm Ø (25 or 50); or an amount of 57.1 g of 3 mm Ø zirconium oxide balls, corresponding to 648 balls – Table S1). Chemical composition for zirconium oxide: a) jars: Zirconium oxide 95%, Hafnium oxide, 1-2%, Magnesium oxide 3.5%, Silicon dioxide: 0.1%, Aluminum oxide 0.1 %, Other 0.1%; b) balls: Zirconium oxide 95% and Yttrium oxide 5% (data source: https://www.fritsch-france.fr/preparation-dechantillons/broyage/broyeurs-planetaires/details/produit/pulverisette-7-classic-line/accessoires/).

Table S1. Weight for the grinding balls used for the experiments.

	Weight for each ball (g)						
	RETSCH			FRITSCH			
Balls diameter (Ø) in mm	**5**	**7**	**10**	**3**	**5**	**7**	**10**
Stainless steel (SS)	0.506	1.388	4.010	-	-	-	-
Zirconium oxide (ZrO_2)	0.414	1.093	3.392	0.088	0.384	0.829	2.948

S1. General experimental procedures

S1.1 CDI-mediated synthesis of amides in a horizontal mixer-mill (MM400 – Retsch).

Method A1 – Compounds 2-5, 7-11 (Table 1).

To a 10 mL ZrO_2 or stainless-steel jar equipped with a single 10 mm diameter ball of the same material (Table S1, Retsch), the acid (1.5 mmol, 1.0 equiv.) and the 1,1'-carbonyldiimidazole (CDI, 1.5 mmol, 1.0. equiv.) were milled for the specified time (30-180 min) at 30 *Hz* in a MM400 mixer mill (*step 1*). After this milling time, the amine (1.35 mmol, 0.9 equiv.) was added, and the reaction mixture was furtherly reacted for the specified time (60-180 min) at 30 *Hz* (*step 2*). Then, 10 mL of water (twice) were added to the crude to precipitate the target compound, which is then isolated by filtration and then dried overnight under vacuum over P_2O_5 to afford the clean product.

S1.2 CDI-mediated synthesis of amides in a vertical mixer-mill (Pulverisette 23, P23 - Fritsch)

Method A2. Compounds 2, 3, 5-11 (Table 1).

To a 5 mL PTFE jar equipped with a single 10 mm Ø ZrO_2 ball (m_{ball} = 2.948 g), the acid (1.5 mmol. 1.0 equiv.) and the 1,1'-carbonyldiimidazole (CDI, 1.5 mmol, 1.0. equiv.) were milled for the specified time (5-90 min) at

50 *Hz* in a Pulverisette 23 (P23, Fritsch®) mixer mill (*step 1*). After this time, the amine (1.35 mmol, 0.9 equiv.) was added, and the reaction mixture is furtherly milled for the specified time (90-180 min.) at 50 *Hz* (*step 2*). Water (5 mL x 3) was added to the jar to precipitate the target compound, isolated by filtration and then dried overnight under vacuum over P_2O_5 to afford the clean product.

S1.3 CDI-mediated synthesis of amides in a planetary ball-mill (P7 Premium Line, P7P – Fritsch)

Method A3. Compounds 9-11 (Table 2, entries 1-9).

To a 45 mL ZrO_2 jar equipped with 25 (Table 2, entries 1 and 4), 50 (Table 2, entries 2, 5 and 7) 5 mm Ø balls or 57.1 g (648) of 3 mm Ø balls (Table 2, entries 3, 6, 8, and 9) of the same material (the weights are given in Table S1), the acid (6 mmol, 1.0 equiv.) and the 1,1'-carbonyldiimidazole (CDI, 6 mmol, 1.0 equiv.) were milled for 60 minutes at 500 rpm in a P7 premium planetary ball mill (Fritsch) (*step 1*). After this time, the amine (5.4 mmol, 0.9 equiv.) was added, and the reaction mixture was furtherly ball-milled for 90 min at 500 rpm (*step 2*). Then, 45 mL of water were added to the crude to precipitate the target compound, isolated by filtration and dried overnight under vacuum over P_2O_5 to afford the clean product. The reaction scale was always 5.4 mmol (0.9 equiv. of amine) except when otherwise specified (Table 2 and experimental part). For entries 1 and 4 (Table 2), the amount was: acid (3 mmol, 1.0 equiv.), 1,1'-carbonyldiimidazole (CDI, 6 mmol, 1.0 equiv.), and amine (2.7 mmol, 0.9 equiv.).

S1.4 ODI-mediated synthesis of amides in a horizontal vibrating ball mill (MM400 – Retsch)

Method B1. Compounds 2-4, 6, 8-10 (Scheme 2).

To a 10 mL ZrO_2 jar equipped with a single 10 mm Ø ball of the same material the acid (1.5 mmol, 1.0 equiv.) and the 1,1'-oxalyldiimidazole (ODI, 1.5 mmol, 1.0 equiv.) were milled for the specified time (30-95 min.) at 30 *Hz*, in a MM400 mixer mill (Retsch) (*step 1*). Then, the amine (1.35 mmol, 0.9 equiv.) was added, and the reaction mixture was furtherly mixed for the specified time (60- 90 min.) at 30 *Hz* (*step 2*). Water was added to the crude 10 mL (twice) to precipitate the target compound, which was isolated by filtration and then dried overnight over P_2O_5 under vacuum to afford the clean product.

S1.5 ODI-mediated synthesis of amides in a planetary ball-mill (P6 or P7 Premium Line Fritsch).

Method B2 - Compounds 9-11 (Table 3).

To a 45 mL ZrO_2 jar equipped with 57.1 g (648) of 3 mm Ø balls of the same material, the acid (6 mmol, 1.0 equiv.) and the 1,1'-oxalyldiimidazole (ODI, 6 mmol, 1.0 equiv.) were milled for the specified time (75 min or 90 min.) at 500 rpm in a P6 planetary ball mill (Fritsch) (*step 1*). Then, the amine (5.4 mmol, 0.9 equiv.) was added and the reaction mixture was furtherly mixed for 90 min. at 500 rpm (*step 2*). 45 mL of water were added to the crude to precipitate the target compound, which was isolated by filtration and then dried overnight over P_2O_5, under vacuum to afford the clean product.

Table S2. Reaction scope and technology assessment sum-up for CDI- and ODI-mediated synthesis of amides.[a] Sum-up of the best experimental conditions for each target.

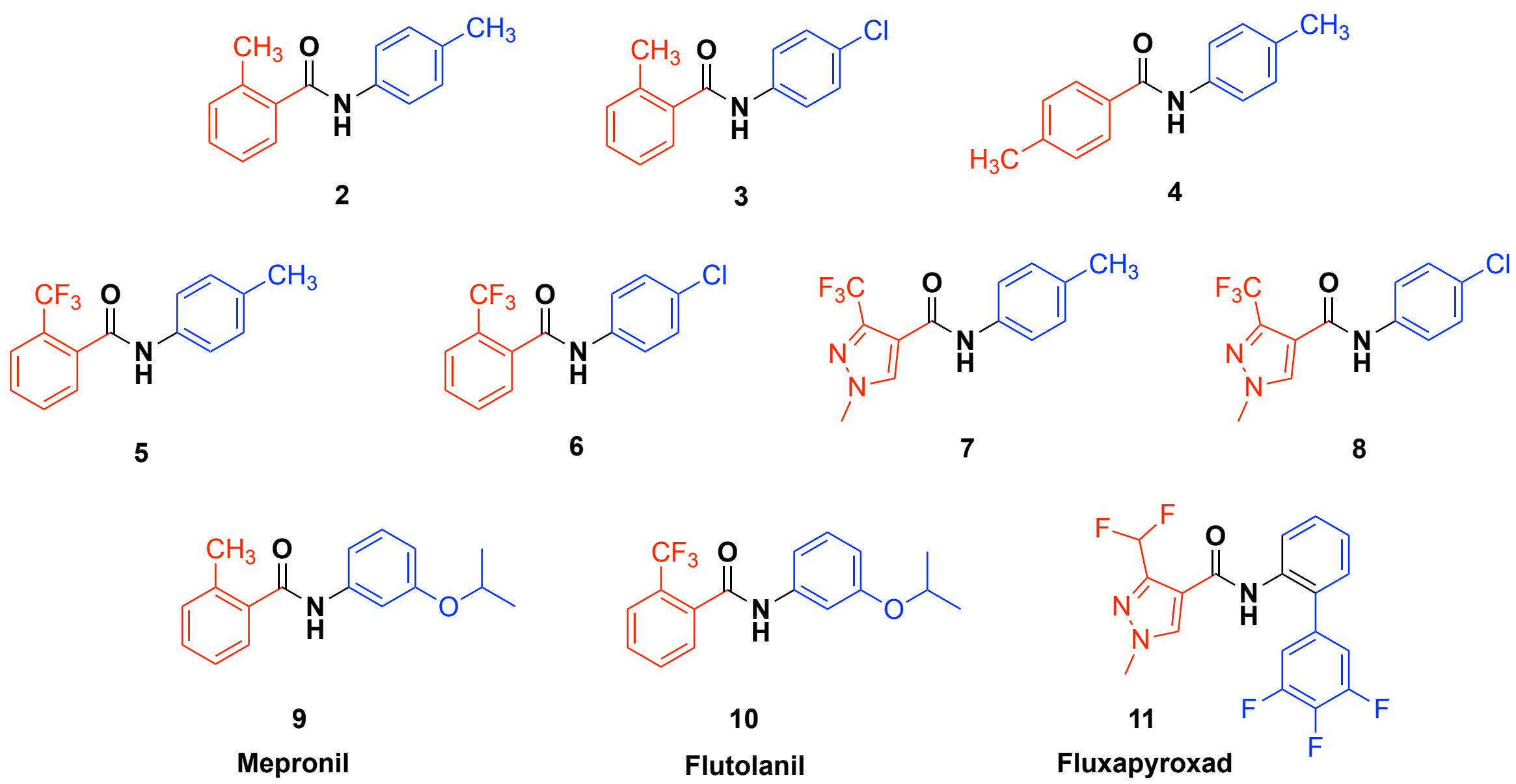

Compound	Mechanochemical method for acid activation / amide bond forming reaction sequence							
	CDI				ODI			
	Vibrating ball-mill		Planetary ball-mill		Vibrating ball-mill		Planetary ball-mill	
	MM400	P23	P6	P7P	MM400	P23	P6	P7P
**2**	A1	A2	n.p.	n.p.	B1	n.p.	n.p.	n.p.
**3**	A1	A2	n.p.	n.p.	B1	n.p.	n.p.	n.p.
**4**	A1	n.p.	n.p.	n.p.	B1	n.p.	n.p.	n.p.
**5**	A1	A2	n.p.	n.p.	B1	n.p.	n.p.	n.p.
**6**	-[b]	A2	n.p.	n.p.	B1	-[b]	n.p.	n.p.
**7**	A1	A2	n.p.	n.p.	B1	n.p.	n.p.	n.p.
**8**	A1	A2	n.p.	n.p.	B1	n.p.	n.p.	n.p.
**9**	A1	A2	A3	A3	B1	n.p.	B2	B2
**10**	A1	A2	A3	A3	B1	n.p.	B2	B2
**11**	-[b]	n.p.	A3	A3	-[b]	n.p.	B2	B2

[a] *Legend*: CDI = 1,1'-carbonyldiimidazole, ODI = 1,1'-oxalyldimidazole, n.p. = not performed; [b] – The product could not be obtained, in any of the mechanochemical conditions explored.

S2. Experimental data and characterizations.

2-Methyl-*N*-(4-tolyl)benzamide (2). CAS [58249-89-1].[1] Compound **2** was prepared according to:

- CDI-mediated synthesis in a vibrating ball mill (Table 1, entry 1):
  - <u>*Method A1*</u> (reaction scale: 1.35 mmol - MM400, Retsch): ZrO_2 or SS grinding media with one 10 mm Ø ball of the same material (*c.f.* for the weight of the balls: Retsch - Table S1) milling at 30 *Hz*. For ZrO_2: *step 1* was 30 min, *step* 2 was 60 min. Yield: 98% (299.4 mg using ZrO_2 as grinding

media). For SS: *step 1* was 30 min, *step* 2 was 90 min. Yield: 94% (286.3 mg, using SS as grinding media);

- *Method A2* (reaction scale: 1.35 mmol, Pulverisette 23, P23 Fritsch): PTFE jar (5 mL) with one 10 mm Ø ZrO_2 ball (m_{ball} = 2.948 g – Table S1), milling at 50 *Hz*; *step 1* was 20 min, *step 2* was 90 min. Yield: 96% (291.4 mg).

• ODI-mediated synthesis in a vibrating ball mill (Scheme 2):

- *Method B1* (reaction scale: 1.35 mmol - MM400, Retsch): ZrO_2 jar (10 mL), with a single 10 mm Ø ZrO_2 ball (m_{ball} = 3.392 g – Table S1), milling at 30 *Hz*; *step 1* was 30 min, *step 2* was 60 min. Yield: 63% (191.8 mg).

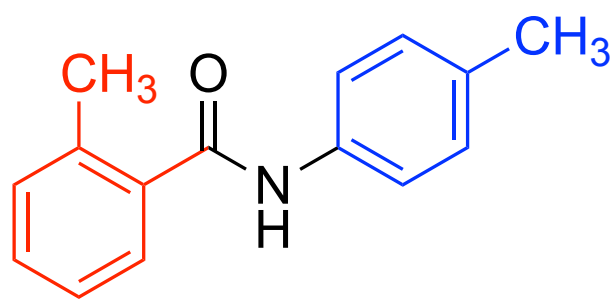

2

White powder; m.p. 142.5°-144.3°C (capillary); by DSC (Figure S1): 141.4°C (lit. 143.5°-144.0°C).[2] **^{1}H NMR** (400 M*Hz*, DMSO-*d*$_6$) *δ* (ppm): 10.18 (s, 1H), 7.62 (d, *J* = 8.4 *Hz*, 2H), 7.44-7.36 (m, 2H), 7.28 (d, *J* = 7.7 *Hz*, 2H), 7.13 (d, *J* = 7.8 *Hz*, 2H), 2.37 (s, 3H), 2.27 (s, 3H). **^{13}C{^{1}H} NMR** (101 M*Hz*, DMSO-*d*$_6$) *δ* (ppm): 167.6, 137.4, 136.8, 135.1, 132.4, 130.5, 129.5, 129.0, 127.1, 125.6, 119.6, 20.5, 19.3. **EI-(+)** *m/z*: 225 [M]$^•$, 208, 119, 91, 65.

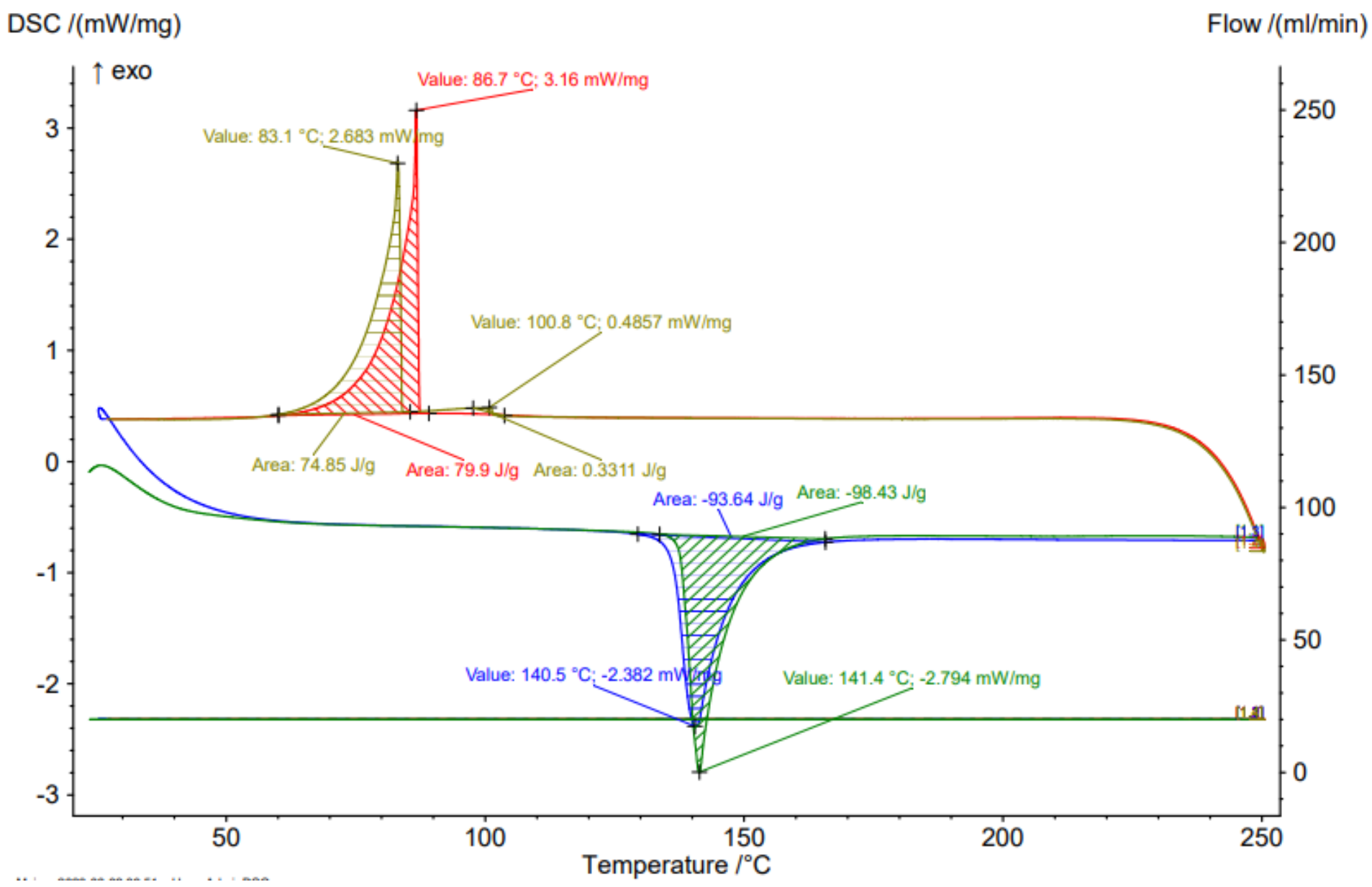

Figure S1. DSC of 2-methyl-*N*-(4-tolyl)benzamide (**2**).

4'-Chloro-toluanilide (3). CAS [65492-69-9].[3] Compound **3** was prepared according to:

• CDI-mediated synthesis in a vibrating ball mill (Table 1, entry 2):

- *Method A1* (reaction scale: 1.35 mmol - MM400, Retsch): ZrO_2 or SS grinding media with one 10 mm Ø ball of the same material (*c.f.* for the weight of the balls: Retsch - Table S1) milling at 30 *Hz*. For ZrO_2: *step 1* was 60 min, *step* 2 was 90 min. Yield: 84% (277.8 mg using ZrO_2 as grinding

media), For SS: *step 1* was 30 min, *step* 2 was 60 min. Yield: 60% (200.5 mg, using SS as grinding media);

- *Method A2* (reaction scale: 1.35 mmol - Pulverisette 23, P23, Fritsch): PTFE jar (5 mL) with one 10 mm Ø ZrO_2 ball (m_{ball} = 2.948 g – Table S1), milling at 50 *Hz*; *step 1* was 20 min, *step 2* was 120 min. Yield: 87% (289.2 mg).

- ODI-mediated synthesis in a vibrating ball mill (Scheme 2):
  - *Method B1* (reaction scale: 1.35 mmol - MM400, Retsch): ZrO_2 jar (10 mL), with a single 10 mm Ø ZrO_2 ball (m_{ball} = 3.392 g – Table S1), milling at 30 *Hz*; *step 1* was 30 min, *step 2* was 60 min. Yield: 63% (207.6 mg).

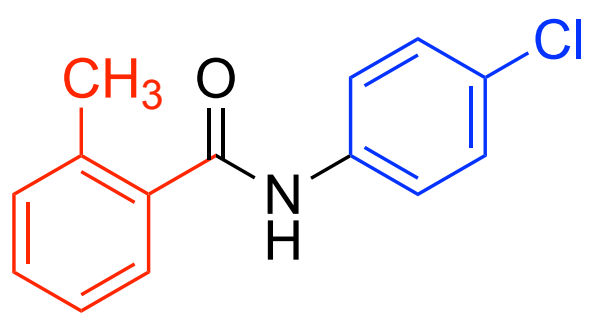

3

White powder, m.p. 133.7°C (capillary), by DSC 135.6°C (Figure S2) (lit. 133°-135°C).[3] **^{1}H NMR** (400 M*Hz*, DMSO-*d*$_6$) *δ* (ppm): 7.78 (d, *J* = 8.41 *Hz*, 2H), 7.47 – 7.38 (m, 4H), 7.32 – 7.28 (m, 2H), 2.38 (s, 3H). **^{13}C{^{1}H} NMR** (101 M*Hz*, DMSO-*d*$_6$) *δ* (ppm):167.9, 138.2, 136.9, 135.2, 130.5, 129.7, 128.6, 127.2, 127.1, 125.7, 121.1, 19.3. **EI-(+)** *m/z* for ^{37}Cl/^{35}Cl: 247/245 [M]$^•$, 119 [M-NH(C_6H_4)Cl]$^•$, 91, 65.

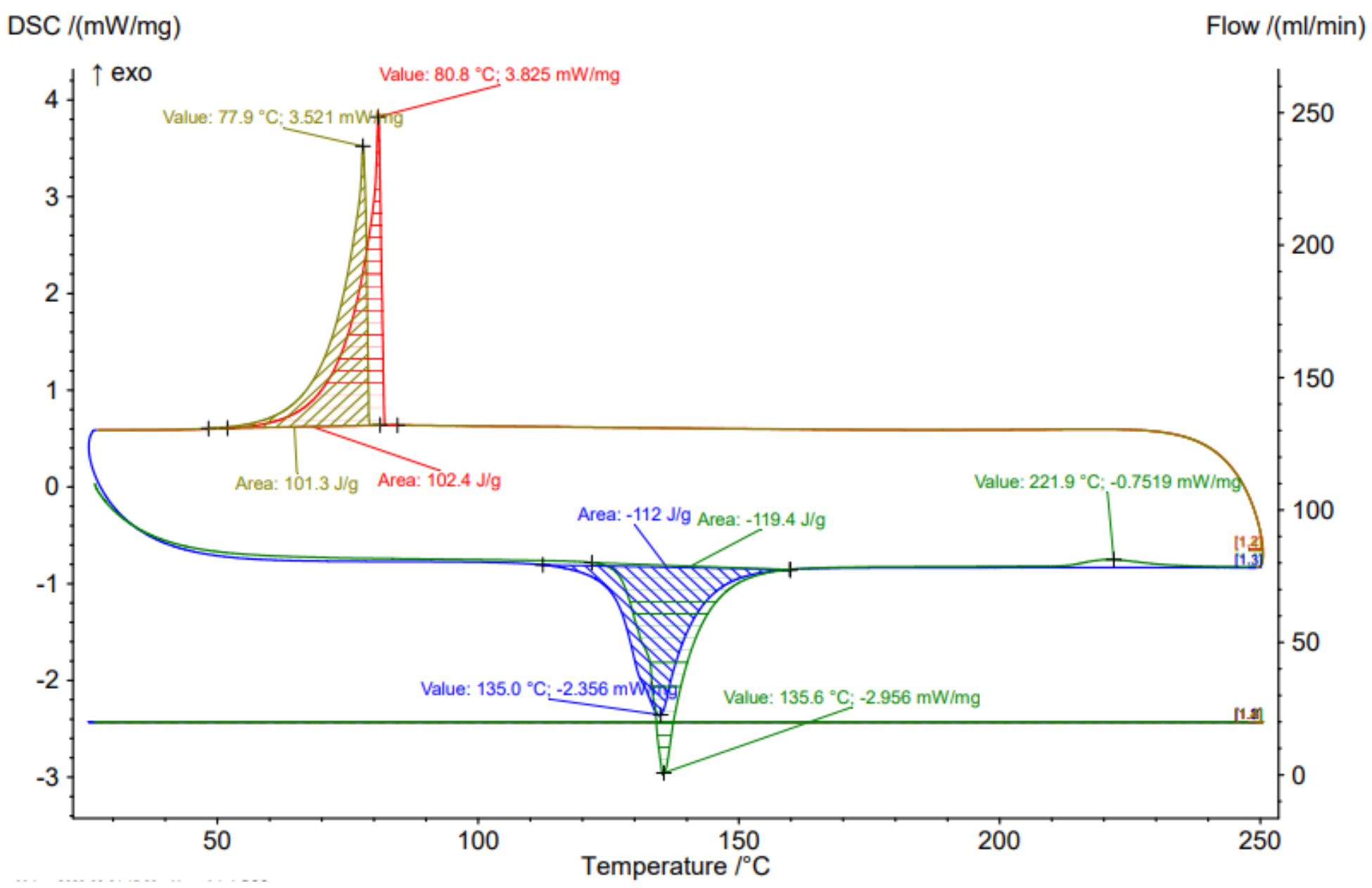

Figure S2. DSC of 4’-chloro-toluanilide (**3**).

***N*-(4-methylphenyl)-4-methyl-benzamide (4).** CAS [6876-66-0].[4] Compound **4** was prepared according to:

- CDI-mediated synthesis in a vibrating ball mill (Table 1, entry 3):
  - *Method A1* (reaction scale: 1.35 mmol - MM400, Retsch): ZrO_2 jar (10 mL) with one 10 mm Ø ball of the same material (m_{ball} = 3.392 g – Table S1), milling at 30 *Hz*. *Step 1* was 30 min, *step* 2 was 60 min. Yield: 90% (272.3 mg).

- ODI-mediated synthesis in a vibrating ball mill (Scheme 2):
    - *Method B1* (reaction scale: 1.35 mmol - MM400, Retsch): ZrO_2 jar (10 mL), with a single 10 mm Ø ZrO_2 ball (m_{ball} = 3.392 g – Table S1), milling at 30 *Hz*; *step 1* was 30 min, *step 2* was 60 min. Yield: 88% (268.4 mg).

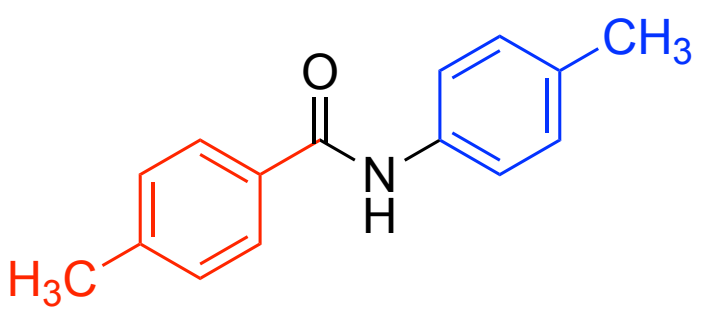

4

White powder, m.p. 162.8°C (lit. 163°C).[5] **^{1}H NMR** (400 M*Hz*, DMSO-d_6)[4] δ (ppm): 10.1 (s, 1H, NH), 7.86 (dt, *J* = 8.08 Hz, *J* =1.94 *Hz*, 2H), 7.64 (dt, *J* = 8.5 *Hz*, *J* = 2.19 *Hz*, 2H), 7.32 (dd, *J* =8.5 *Hz*, *J* = 0.7 *Hz,* 2H), 7.14 (dd, *J* = 8.9 *Hz*, J = 0.48 *Hz,* 2H), 2.38 (s, 3H), 2.27 (s, 3H). **^{13}C{^{1}H}NMR NOE** (101 M*Hz*, DMSO-d_6)[4] δ(ppm): 141.4, 136.7, 132.5, 129.0, 128.9, 127.6, 120.4, 21.0. **EI (+)** *m/z*: 225 [M]$^{\bullet}$, 119, 91, 65.

***N*-(4-methylphenyl)-2-(trifluoromethyl)benzamide (5).** CAS [362718-30-7]. Compound **5** was prepared according to:

- CDI-mediated synthesis in a vibrating ball mill (Table 1, entry 4):
    - *Method A1* (reaction scale: 1.35 mmol - MM400, Retsch): ZrO_2 or SS grinding media with one 10 mm Ø ball of the same material (*c.f.* for the weight of the balls: Retsch - Table S1) milling at 30 *Hz*. For ZrO_2: *step 1* was 60 min, *step* 2 was 90 min. Yield: 82% (310.6 mg, using ZrO_2 as grinding media), For SS: *step 1 (cycled)* four cycles of 15 min, with a rest period of 5 min in between two consecutive cycles (total milling time 60 min), *step* 2 was 180 min. Yield: 73% (276.2 mg, using SS as grinding media);
    - *Method A2* (reaction scale 1.35 mmol - Pulverisette 23, P23 Fritsch): PTFE jar (5 mL) with one 10 mm Ø ZrO_2 ball (m_{ball} = 2.948 g – Table S1), milling at 50 *Hz*; *step 1* was 90 min, *step 2* was 120 min. Yield: 70 % (265.4 mg).
- ODI-mediated synthesis in a vibrating ball mill (Scheme 2):
    - *Method B1* (reaction scale: 1.35 mmol - MM400, Retsch): ZrO_2 jar (10 mL), with a single 10 mm Ø ZrO_2 ball (m_{ball} = 3.392 g – Table S1), milling at 30 *Hz*; *step 1* was 90 min, *step 2* was 60 min. Yield: 38% (141.6 mg).

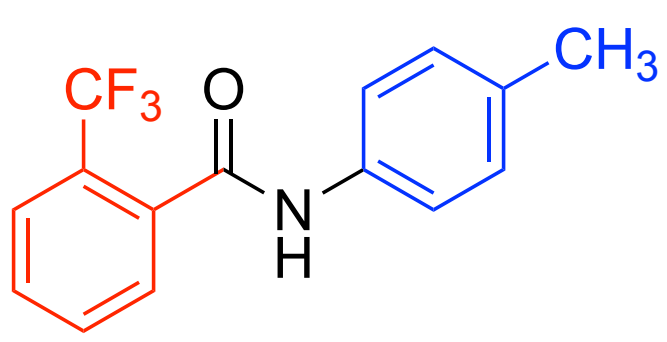

5

White powder, m.p. 135.6-147.2°C (capillary). **^{1}H NMR** (400 M*Hz*, DMSO-d_6) δ (ppm): 10.44 (s, 1H), 7.85-7.77 (m, 2H), 7.70 (dd, *J* = 7.6 *Hz*, *J* = 5.4 *Hz*, 2H), 7.57-7.54 (m, 2H), 7.15 (d, *J* = 8.4 *Hz*, 2H), 2.28 (s, 3H). **^{13}C{^{1}H}NMR NOE** (101 M*Hz*, DMSO-d_6) δ (ppm): 165.8, 136.9, 133.3, 130.4, 129.6, 129.0, 120.1, 100.0, 21.0. **^{19}F{^{1}H}NMR** (376 M*Hz*, DMSO-d_6) δ (ppm): -57.93 (s). **EI-**

(+) *m/z*: 279 [M]•, 173 [M-NH(C_6H_4)CH_3]•. **HRMS ESI-(+)** *m/z* [M+H]+ calcd. for $C_{15}H_{13}F_3NO$ 280.0944, found 280.0947.

***N*-(4-chlorophenyl)-2-(trifluoromethyl)benzamide (6).** CAS [425616-11-1]. Compound **6** was prepared according to:

- CDI-mediated synthesis in a vibrating ball mill (Table 1, entry 5):
  - <u>*Method A2*</u> (reaction scale: 1.35 mmol - Pulverisette 23, P23 Fritsch): PTFE jar (5 mL) with one 10 mm Ø ZrO_2 ball (m_{ball} = 2.948 g – Table S1), milling at 50 *Hz*; *step 1* was 90 min, *step 2* was 180 min. Yield: 60% (243.2 mg).
- ODI-mediated synthesis in a vibrating ball mill (Scheme 2):
  - <u>*Method B1*</u> (reaction scale 1.35 mmol - MM400, Retsch): PFTE jar (10 mL), with a single 10 mm Ø ZrO_2 ball (m_{ball} = 3.392 g – Table S1), milling at 30 *Hz*; *step 1* was 90 min, *step 2* was 90 min. Yield: 41% (166.2 mg).

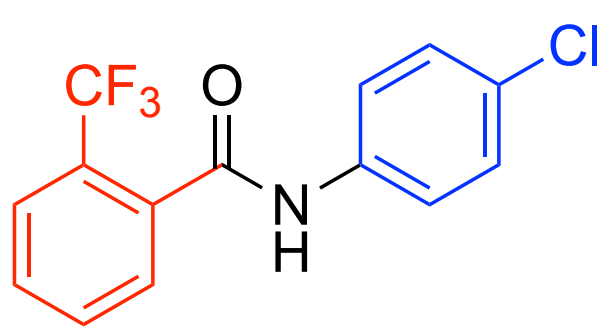

6

White powder, m.p. 137.2-140.4°C (capillary), DSC 135.5°C (Figure S3). **^{1}H NMR** (400 M*Hz*, DMSO-d_6) *δ* (ppm): 10.71 (s, 1H), 7.88-7.80 (m, 2H), 7.75-7.72 (m, 4H), 7.43 (d, *J* = 8.8 *Hz*, 2H). **^{13}C{^{1}H}NMR NOE** (101 M*Hz*, DMSO-d_6) *δ* (ppm): 166.1, 138.3, 136.4, 133.1, 130.7, 129.1 (d, *J* = 21.8 Hz, CF_3), 128.0, 125.6, 122.9, 121.7. **^{19}F{^{1}H}NMR** (376 M*Hz*, DMSO-d_6) *δ*(ppm): -57.93 (s). **EI-(+)** *m/z* for ^{37}Cl/^{35}Cl: 301/299 [M]•, 173 [M-NH(C_6H_4)Cl]•. **HRMS ESI-(+)** *m/z* [M+H]+ calcd. for $C_{14}H_{10}ClF_3NO$ 300.0398, found 300.0399.

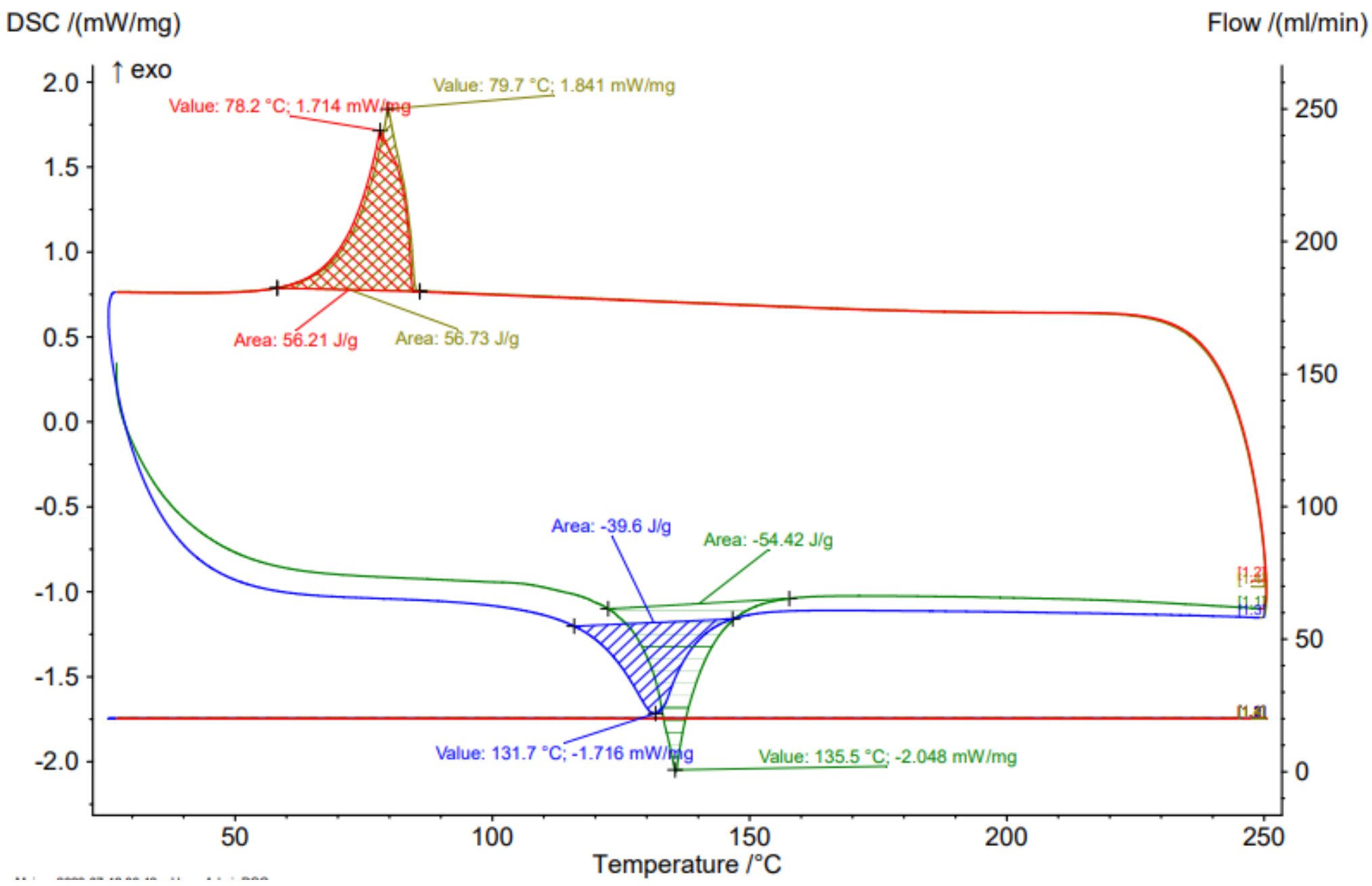

Figure S3. DSC of *N*-(4-chlorophenyl)-2-(trifluoromethyl)benzamide (**6**).

1-Methyl-*N*-(p-tolyl)-3-(trifluoromethyl)-1H-pyrazole-4-carboxamide (7). CAS [1561088-36-5]. Compound **7** was prepared according to:

- CDI-mediated synthesis in a vibrating ball mill (Table 1, entry 6):
  - <u>*Method A1*</u> (reaction scale: 1.35 mmol - MM400, Retsch): ZrO_2 or SS grinding media with one 10 mm Ø ball of the same material (*c.f.* for the weight of the balls: Retsch - Table S1) milling at 30 *Hz*. For ZrO_2: *step 1* was 60 min, *step* 2 was 90 min. Yield: 91% (348 mg, using using ZrO_2 as grinding media), For SS: *step 1* was 60 min, *step* 2 was 90 min. Yield: 94% (361.3 mg, using SS as grinding media);
  - <u>*Method A2*</u> (reaction scale: 1.35 mmol - Pulverisette 23, P23 Fritsch): PTFE jar (5 mL) with one 10 mm Ø ZrO_2 ball (m_{ball} = 2.948 g – Table S1), milling at 50 *Hz*; *step 1* was 5 min, *step 2* was 90 min. Yield: 63% (242.9 mg).
- ODI-mediated synthesis in a vibrating ball mill (Scheme 2):
  - <u>*Method B1*</u> (reaction scale: 1.35 mmol - MM400, Retsch): ZrO_2 jar (10 mL), with a single 10 mm Ø ZrO_2 ball (m_{ball} = 3.392 g – Table S1), milling at 30 *Hz*; *step 1* was 60 min, *step 2* was 60 min. Yield: 59% (224.0 mg).

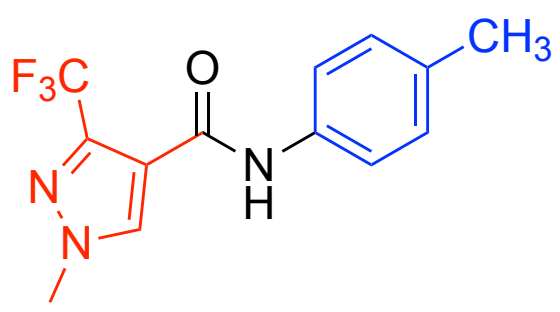

7

White powder, m.p. 158.1-160.5°C (capillary), DSC 156.0°C (Figure S4). **^{1}H NMR** (400MHz, DMSO-d_6) δ(ppm): 8.49 (s, 1H), 7.55 (d, *J* = 8.4 *Hz*, 2H), 7.13 (d, *J* = 8.2 *Hz*, 2H), 3.98 (s, 3H), 2.27 (s, 3H). **^{13}C{^{1}H}NMR NOE** (101MHz, DMSO-d_6) δ(ppm): 159.0 (s, C=O), 139.5 (d, *J* = 37.46 Hz, CF_3), 136.8 (s), 134.7 (s), 133.0 (s), 129.6 (s), 121.1 (d, *J* = 268.87 Hz, C-CF_3), 120.0 (s), 117.2 (s), 31.0 (s), 20.9 (s). **^{19}F{^{1}H}NMR** (376MHz, DMSO-d_6) δ(ppm): -59.58 (s, 3F). **EI-(+)** *m/z*: 283 $[M]^{\bullet}$, 177 $[M\text{-}NH(C_6H_4)CH_3]^{\bullet}$. **HRMS ESI-(+)** *m/z* $[M+H]^+$ calcd. for $C_{13}H_{13}F_3N_3O$ 284.1011, found: 284.1005.

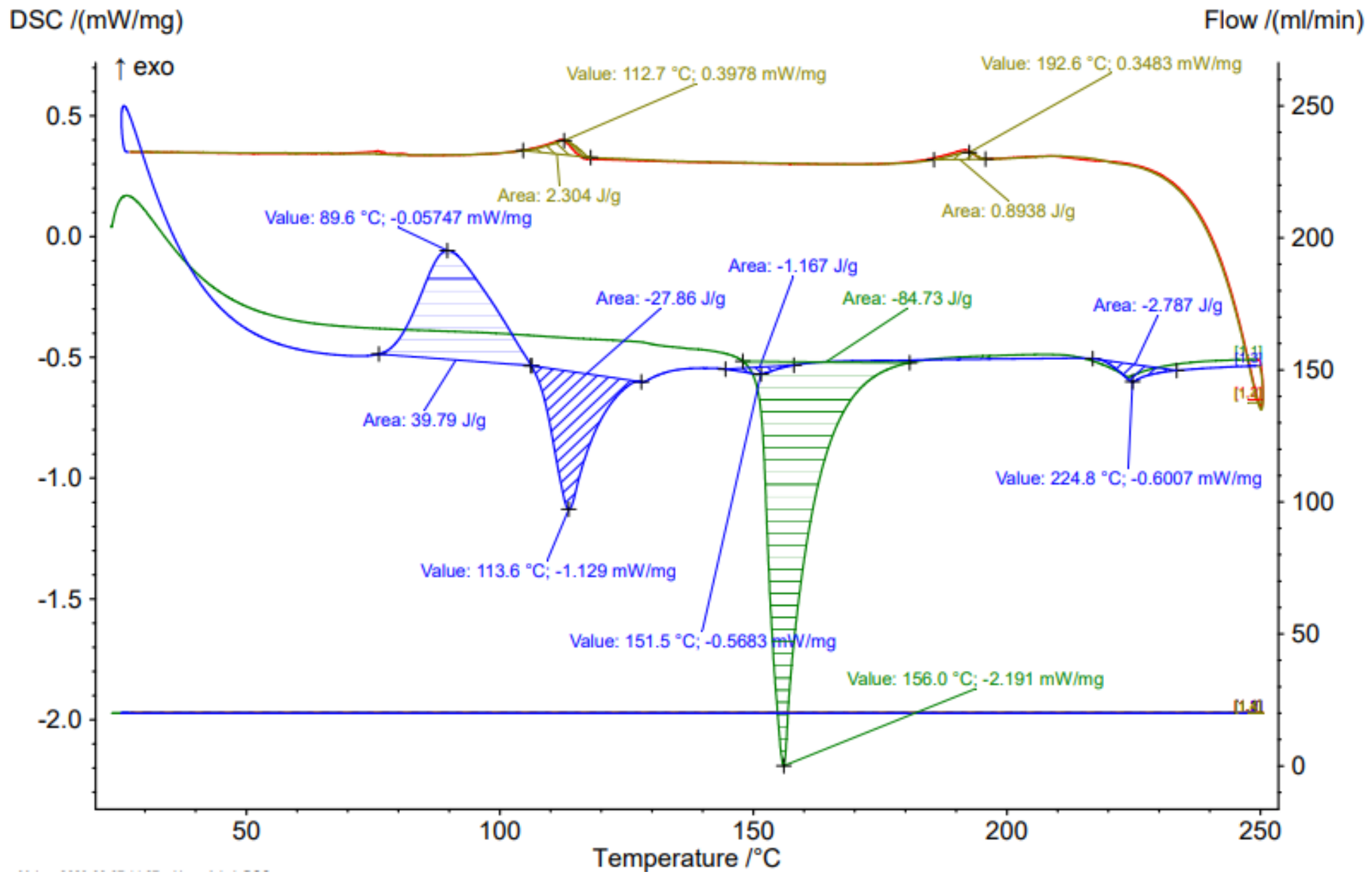

Figure S4. DSC of 1-methyl-*N*-(p-tolyl)-3-(trifluoromethyl)-1H-pyrazole-4-carboxamide (**7**).

1-Methyl-*N*-(*p*-chloro)-3-(trifluoromethyl)-1H-pyrazole-4-carboxamide (8). CAS [956705-90-1]. Compound **8** was prepared according to:

- CDI-mediated synthesis in a vibrating ball mill (Table 1, entry 7):
  - <u>*Method A1*</u> (reaction scale: 1.35 mmol - MM400, Retsch): ZrO_2 or SS grinding media with one 10 mm Ø ball of the same material (*c.f.* for the weight of the balls: Retsch - Table S1) milling at 30 *Hz*. For ZrO_2: *step 1* was 60 min, *step* 2 was 90 min. Yield: 85% (348 mg, using ZrO_2 as grinding media), For SS: *step 1* was 60 min, *step* 2 was 90 min. Yield: 67% (273.8 mg, using SS as grinding media);
  - <u>*Method A2*</u> (reaction scale: 1.35 mmol - Pulverisette 23, P23 Fritsch): PTFE jar (5 mL) with one 10 mm Ø ZrO_2 ball (m_{ball} = 2.948 g – Table S1), milling at 50 *Hz*; *step 1* was 5 min, *step 2* was 90 min. Yield: 71% (289.2 mg).
- ODI-mediated synthesis in a vibrating ball mill (Scheme 2):
  - <u>*Method B1*</u> (reaction scale 1.35 mmol - MM400, Retsch): ZrO_2 jar (10 mL), with a single 10 mm Ø ZrO_2 ball (m_{ball} = 3.392 g – Table S1), milling at 30 *Hz*; *step 1* was 75 min, *step 2* was 90 min. Yield: 80% (327.3 mg).

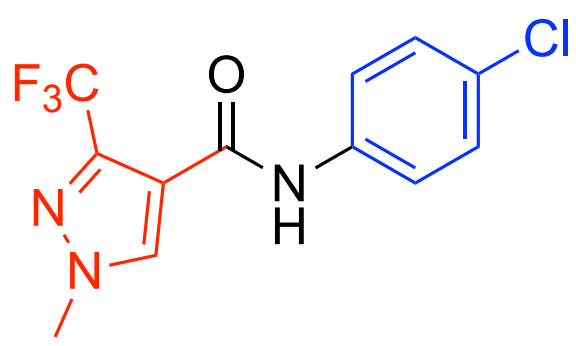

8

White powder, m.p. 153.1-158.8°C (capillary), by DSC: 156.4°C (Figure S5). **^{1}H NMR** (400 M*Hz*, DMSO-*d*$_6$) *δ* (ppm): 10.24 (s, 1H), 8.52 (s, 1H), 7.71 (dt *J* = 9.0 *Hz*, *J* = 2.7 *Hz*, 2H), 7.39 (dt *J* = 8.9 *Hz*, *J* = 2.7 *Hz*, 2H), 3.98 (s, 3H). **^{13}C{^{1}H}NMR NOE** (101 M*Hz*, DMSO-*d*$_6$) *δ* (ppm): 159.2 (s, C=O), 139.8 (d, *J* = 37.1 *Hz*, CF_3), 138.3, 134.9, 129.1, 127.7, 121.8, 121.4 (d, *J* = 268.8 *Hz*, C-CF_3), 116.8, 40.0. **^{19}F{^{1}H}NMR** (376 M*Hz*, DMSO-*d*$_6$) *δ*(ppm): -59.76 (s, 3F). **EI-(+)** *m/z* for ^{37}Cl/^{35}Cl: 303/305 [M]$^{•}$, 177 [M-NH(C_6H_4)Cl]$^{•}$. **HRMS ESI-(+)** *m/z* [M+H]$^+$ *calcd.* for $C_{12}H_{10}ClF_3N_3O$ 304.0459, *found*: 304.0462.

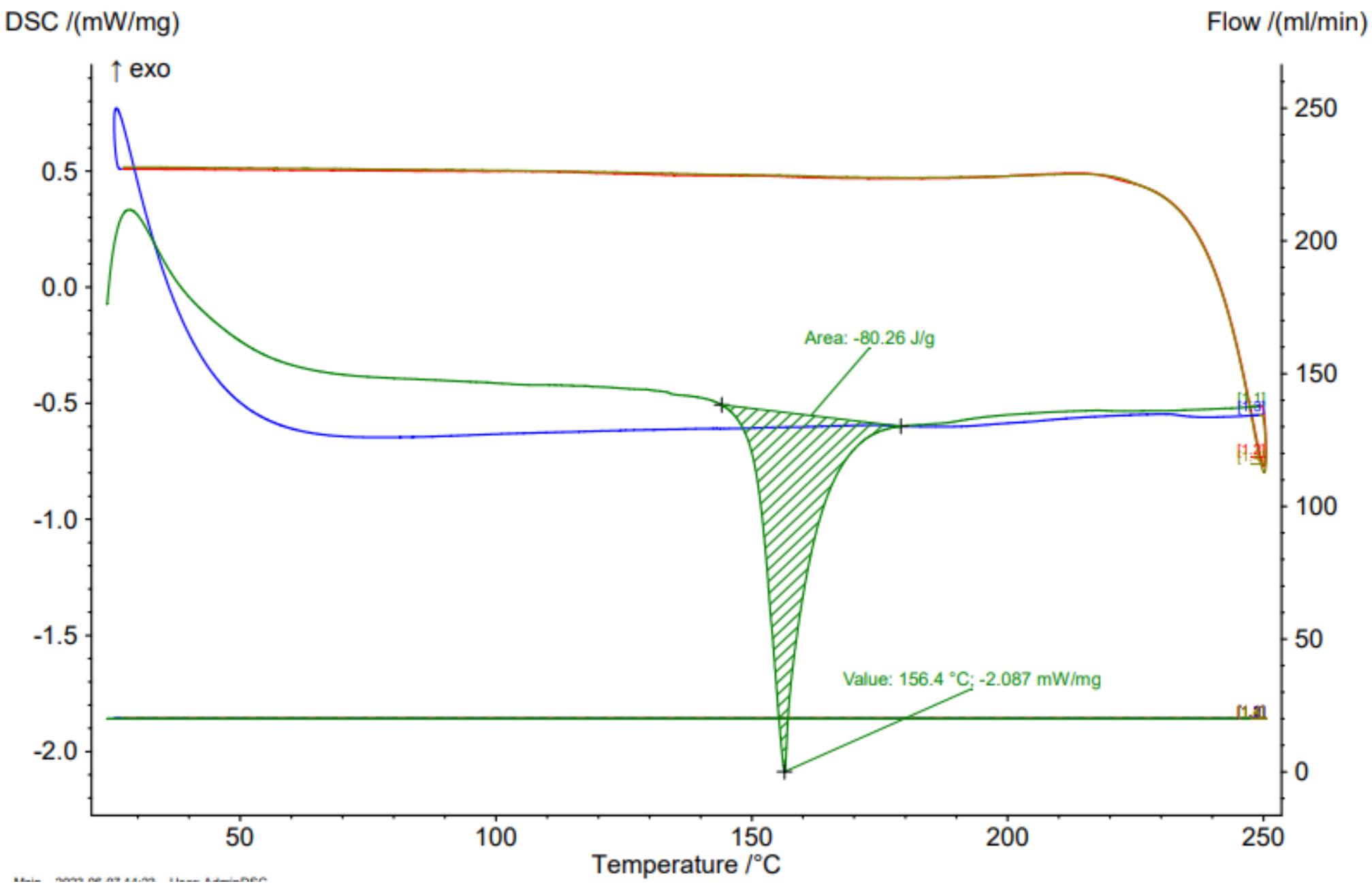

Figure S5. DSC of 1-methyl-*N*-(*p*-chloro)-3-(trifluoromethyl)-1H-pyrazole-4-carboxamide (**8**).

2-Methyl-N-(3-propan-2-yloxyphenyl)benzamide (Mepronil) (9). CAS [55814-41-0].[6] Compound **9** was prepared according to:

- CDI-mediated synthesis in a vibrating ball mill (Table 1, entry 8):
  - <u>*Method A1*</u> (reaction scale: 1.35 mmol - MM400, Retsch): ZrO_2 or SS grinding media with one 10 mm Ø ball of the same material (*c.f.* for the weight of the balls: Retsch - Table S1) milling at 30 *Hz*. For ZrO_2: *step 1* was 30 min, *step* 2 was 90 min. Yield: 66% (240 mg, using ZrO_2 as grinding media), For SS: *step 1* was 60 min, *step* 2 was 90 min. Yield: 70% (254 mg, using a 5 mL SS jar as grinding media);
  - <u>*Method A2*</u> (reaction scale: 1.35 mmol - Pulverisette 23, P23 Fritsch): PTFE jar (5 mL) with one 10 mm Ø ZrO_2 ball (m_{ball} = 2.948 g – Table S1), milling at 50 *Hz*; *step 1* was 20 min, *step 2* was 90 min. Yield: 61% (223.0 mg).
- CDI-mediated synthesis in a planetary ball mill (Table 2, entries 1-3 and Table 3, entry 1):

- *Method A3* (P7 Premium Line, Fritsch - Table 2):
  - *a)* entry 1 (reaction scale: 2.7 mmol): ZrO_2 jar (20 mL), with 25 ZrO_2 balls of 5 mm Ø (m_{ball} = 0.384 g – Table S1), milling at 500 rpm *Hz*; *step 1* was 60 min, *step 2* was 90 min. Yield: 3% (21 mg);
  - *b)* entry 2 (reaction scale: 5.4 mmol): ZrO_2 jar (45 mL), with 50 ZrO_2 balls of 5 mm Ø (m_{ball} = 0.384 g – Table S1), milling at 500 rpm *Hz*; *step 1* was 60 min, *step 2* was 90 min. Yield: 73% (1.06 g);
  - *c)* entry 3 (reaction scale: 5.4 mmol): ZrO_2 jar (45 mL), with 57.1 g (648) ZrO_2 balls of 3 mm Ø (m_{ball} = 0.088 g – Table S1), milling at 500 rpm *Hz*; *step 1* was 60 min, *step 2* was 90 min. Yield: 60% (880 mg).
- *Method A3* (P6 Fritsch - Table 3):
  - *d)* entry 1 (reaction scale: 5.4 mmol): ZrO_2 jar (45 mL), with 50 ZrO_2 balls of 5 mm Ø (m_{ball} = 0.384 g – Table S1), milling at 500 rpm *Hz*; *step 1* was 60 min, *step 2* was 90 min. Yield: 16% (231.7 mg);

• ODI-mediated synthesis in a vibrating ball mill (Scheme 2):

- *Method B1* (reaction scale: 1.35 mmol - MM400, Retsch): ZrO_2 jar (10 mL), with a single 10 mm Ø ZrO_2 ball (m_{ball} = 3.392 g – Table S1), milling at 30 *Hz*; *step 1* was 30 min, *step 2* was 90 min. Yield: 90% (327.2 mg).

• ODI-mediated synthesis in a planetary ball mill (Table 3, entry 1):

- *Method B2* (reaction scale: 5.4 mmol)
  - *a)* entry 1 (P6 Fritsch): ZrO_2 jar (45 mL), with 57.1 g (648) ZrO_2 balls of 3 mm Ø (m_{ball} = 0.088 g – Table S1), milling at 500 rpm *Hz*; *step 1* was 75 min, *step 2* was 90 min. Yield: 93% (1.35 g);
  - *b)* entry 1 (P7 Premium Line, Fritsch): ZrO_2 jar (45 mL), with 57.1 g (648) ZrO_2 balls of 3 mm Ø (m_{ball} = 0.088 g – Table S1), milling at 500 rpm *Hz*; *step 1* was 75 min, *step 2* was 90 min. Yield: 83% (1.20 g).

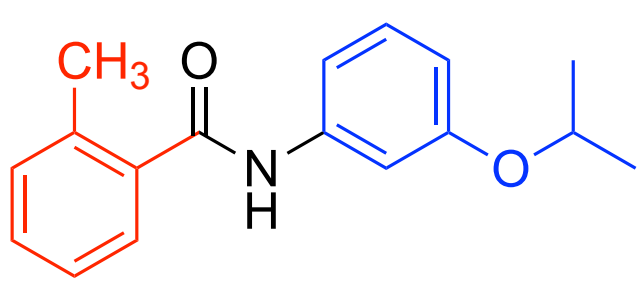

9

White powder, m.p. 92.5°-93.3°C (capillary), by DSC: 92.2°C (Figure S6) (lit. 92°-94°C);[7] **¹H NMR** (400 M*Hz*, $CDCl_3$) *δ* (ppm):[8] 7.48 – 7.35 (m, 4H), 7.28 – 7.21 (m, 3H), 7.04 (d, *J* = 7.5 *Hz*, 1H), 6.69 (dd, *J* = 1.7 *Hz*, *J* = 8.2 *Hz*, 1H), 4.59 (quint, *J* = 5.9 *Hz*, 1H), 2.50 (s, 3H), 1.35 (d, *J* = 6.0 *Hz*, 6H); **¹H NMR** (400 M*Hz*, DMSO-d_6) *δ* (ppm): 10.24 (s, 1H), 7.46-7.38 (m, 3H), 7.33-7.19 (m, 4H), 6.66 (d, *J* = 7.7 *Hz*, 1H), 4.56 (quint, *J* = 6.88 *Hz*, 1H), 2.39 (s, 3H), 1.28 (d, *J* = 5.9 *Hz*, 6H); **$^{13}C\{^1H\}$NMR NOE** (101 M*Hz*, $CDCl_3$) *δ*(ppm):[8] 168.1, 158.7, 139.3, 136.7, 136.6, 131.4, 130.4, 129.9, 126.7, 126.0, 112.31, 111.9, 107.7, 70.2, 22.2, 20.0; **$^{13}C\{^1H\}$NMR NOE** (101 M*Hz*, DMSO-d_6) *δ*(ppm): 168.3, 158.2,

141.0, 137.8, 135.6, 130.9, 130.1, 129.9, 127.6, 126.1, 112.2, 111.0, 107.6, 69.6, 22.3, 19.8. **EI-(+)** *m/z*: 269 [M]$^{\bullet}$, 119 [M-NH(C_6H_4)OC_3H_7]$^{\bullet}$.

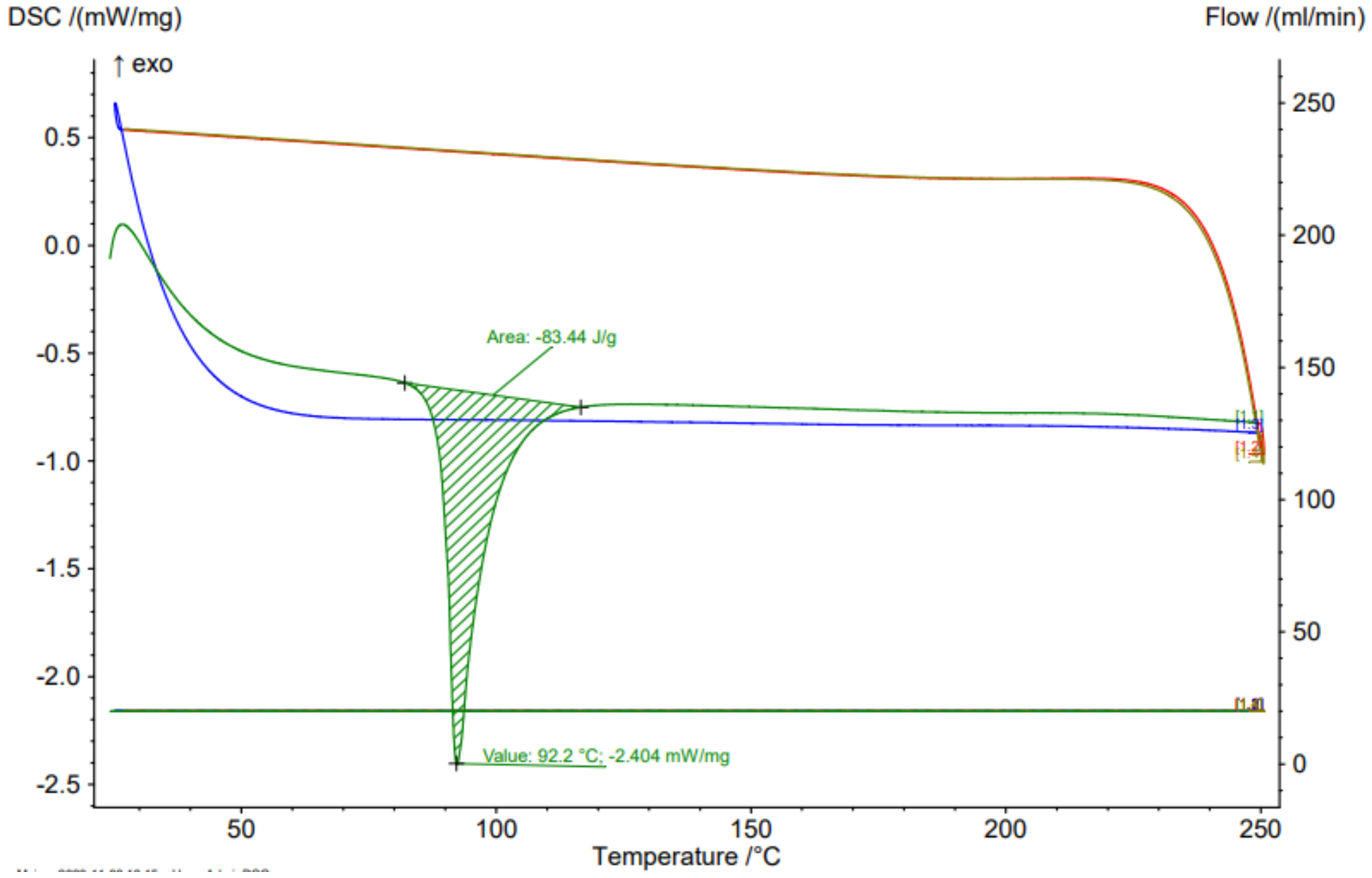

Figure S6. DSC of Mepronil (**9**).

***N*-(3-Isopropoxyphenyl)-2-(trifluoromethyl)benzamide (Flutolanil) (10).** CAS [66332-96-5]. Compound **10** was prepared according to:

- CDI-mediated synthesis in a vibrating ball mill (Table 1, entry 9):
  - <u>*Method A1*</u> (MM400, Retsch): ZrO_2 or SS grinding media with one 10 mm Ø ball of the same material (*c.f.* for the weight of the balls: Retsch - Table S1) milling at 30 *Hz*. For ZrO_2 (reaction scale: 1.35 mmol): *step 1* was 60 min, *step* 2 was 90 min. Yield: 50% (217 mg, using ZrO_2 as grinding media). For SS (reaction scale: 1.50 mmol): *step 1* was 60 min, *step* 2 was 90 min. Yield: 46% (223 mg, using a 5 mL SS jar);
  - <u>*Method A2*</u> (reaction scale: 1.35 mmol – Pulverisette 23, P23 Fritsch): PTFE jar (5 mL) with one 10 mm Ø ZrO_2 ball (m_{ball} = 2.948 g – Table S1), milling at 50 *Hz*; *step 1* was 90 min, *step 2* was 120 min. Yield: 32% (135.0 mg).
- CDI-mediated synthesis in a planetary ball mill (Table 2, entries 4-6 and table 3, entry 2):
  - <u>*Method A3*</u> (P7 Premium, Fritsch - Table 2):
    - *a)* entry 4 (reaction scale: 2.7 mmol): ZrO_2 jar (20 mL), with 25 ZrO_2 balls of 5 mm Ø (m_{ball} = 0.384 g – Table S1), milling at 500 rpm *Hz*; *step 1* was 60 min, *step 2* was 90 min. Yield: 0%;

b) entry 5 (reaction scale: 5.4 mmol): ZrO_2 jar (45 mL), with 50 ZrO_2 balls of 5 mm Ø (m_{ball} = 0.384 g – Table S1), milling at 500 rpm *Hz*; *step 1* was 60 min, *step 2* was 90 min. Yield: 52% (914 mg);

c) entry 6 (reaction scale: 5.4 mmol): ZrO_2 jar (45 mL), with 57.1 g (648) ZrO_2 balls of 3 mm Ø (m_{ball} = 0.088 g – Table S1), milling at 500 rpm *Hz*; *step 1* was 60 min, *step 2* was 90 min. Yield: 37% (653 mg);

- Method A3 (P6 Fritsch - Table 3, entry 2):

d) entry 2 (reaction scale: 5.4 mmol): ZrO_2 jar (45 mL), with 50 ZrO_2 balls of 5 mm Ø (m_{ball} = 0.384 g – Table S1), milling at 500 rpm *Hz*; *step 1* was 60 min, *step 2* was 90 min. Yield: 21% (360.8 mg).

- ODI-mediated synthesis in a vibrating ball mill (Scheme 2):
  - Method B1 (reaction scale: 1.35 mmol - MM400 Retsch): ZrO_2 jar (10 mL), with a single 10 mm Ø ZrO_2 ball (m_{ball} = 3.392 g – Table S1), milling at 30 *Hz*; *step 1* was 60 min, *step 2* was 60 min. Yield: 50% (214.0 mg).
- ODI-mediated synthesis in a planetary ball mill (Table 3, entry 2):
  - Method B2 (reaction scale: 5.4 mmol):

a) entry 2 (P6 Fritsch): ZrO_2 jar (45 mL), with 57.1 g (648) ZrO_2 balls of 3 mm Ø (m_{ball} = 0.088 g – Table S1), milling at 500 rpm *Hz*; *step 1* was 75 min, *step 2* was 90 min. Yield: 64% (1.11 g).

b) entry 2 (P7 Premium Fritsch): ZrO_2 jar (45 mL), with 57.1 g (648) ZrO_2 balls of 3 mm Ø (m_{ball} = 0.088 g – Table S1), milling at 500 rpm *Hz*; *step 1* was 75 min, *step 2* was 90 min. Yield: 55% (0.954 g).

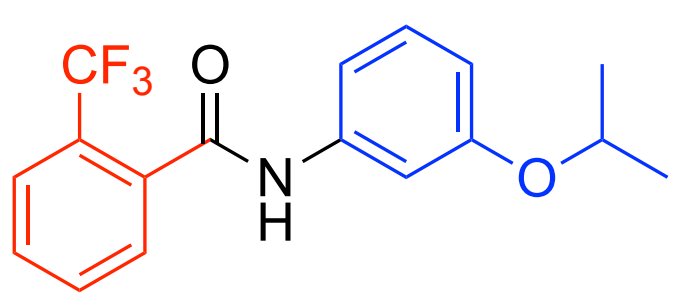

10

White powder, m.p. 100.1°-100.4°C (capillary), by DSC: 99.9°C (Figure S7) (lit. 95°-97°C,[9] 102-103°C[10]); **^{1}H NMR** (400 M*Hz*, $CDCl_3$) δ (ppm):[10] 7.75 (d, *J* = 7.7 *Hz*, 1H), 7.66 – 7.57 (m, 3H), 7.39 (s, 1H), 7.33 (s, 1H), 7.26 – 7.22 (m, 1H), 7.03 (d, *J* = 7.8 *Hz*, 1H), 6.72 (dd, *J* = 1.9 *Hz*, *J* = 8.5 *Hz*, 1H), 4.59 (quint, *J* = 6.0 *Hz*, 1H), 1.35 (d, *J* = 6.0 *Hz*, 6H); **^{1}H NMR** (400 M*Hz*, DMSO-d_6) δ (ppm): 10.49 (s, 1H), 7.86-7.78 (m, 2H), 7.73-7.68 (m, 2H), 7.33 (s, 1H), 7.22-7.18 (m, 2H), 6.67 (d, *J* = 7.3 *Hz*, 1H), 4.55 (quint, *J* = 6.0 *Hz*, 1H), 1.27 (d, *J* = 6.0 *Hz*, 6H). **^{13}C{^{1}H}NMR NOE** (101 M*Hz*, $CDCl_3$) δ(ppm):[10] 165.7, 158.8, 138.7, 136.0, 132.4, 130.4, 130.0, 128.8, 126.7 (q, *J* = 4.6 *Hz*), 112.7, 112.2, 108.0, 70.2, 22.2; **^{13}C{^{1}H}NMR** (101 M*Hz*, DMSO-d_6) δ (ppm): 166.0, 158.2, 140.6, 133.1, 130.5, 130.1, 129.0, 126.8, 112.2, 111.3, 107.6, 69.7, 22.3.**^{19}F{^{1}H}NMR** (376 M*Hz*, DMSO-d_6)[7] δ (ppm): -57.92 (s). **EI-(+)** *m/z*: 323 $[M]^{\bullet}$, 173 $[M-NH(C_6H_4)OC_3H_7]^{\bullet}$.

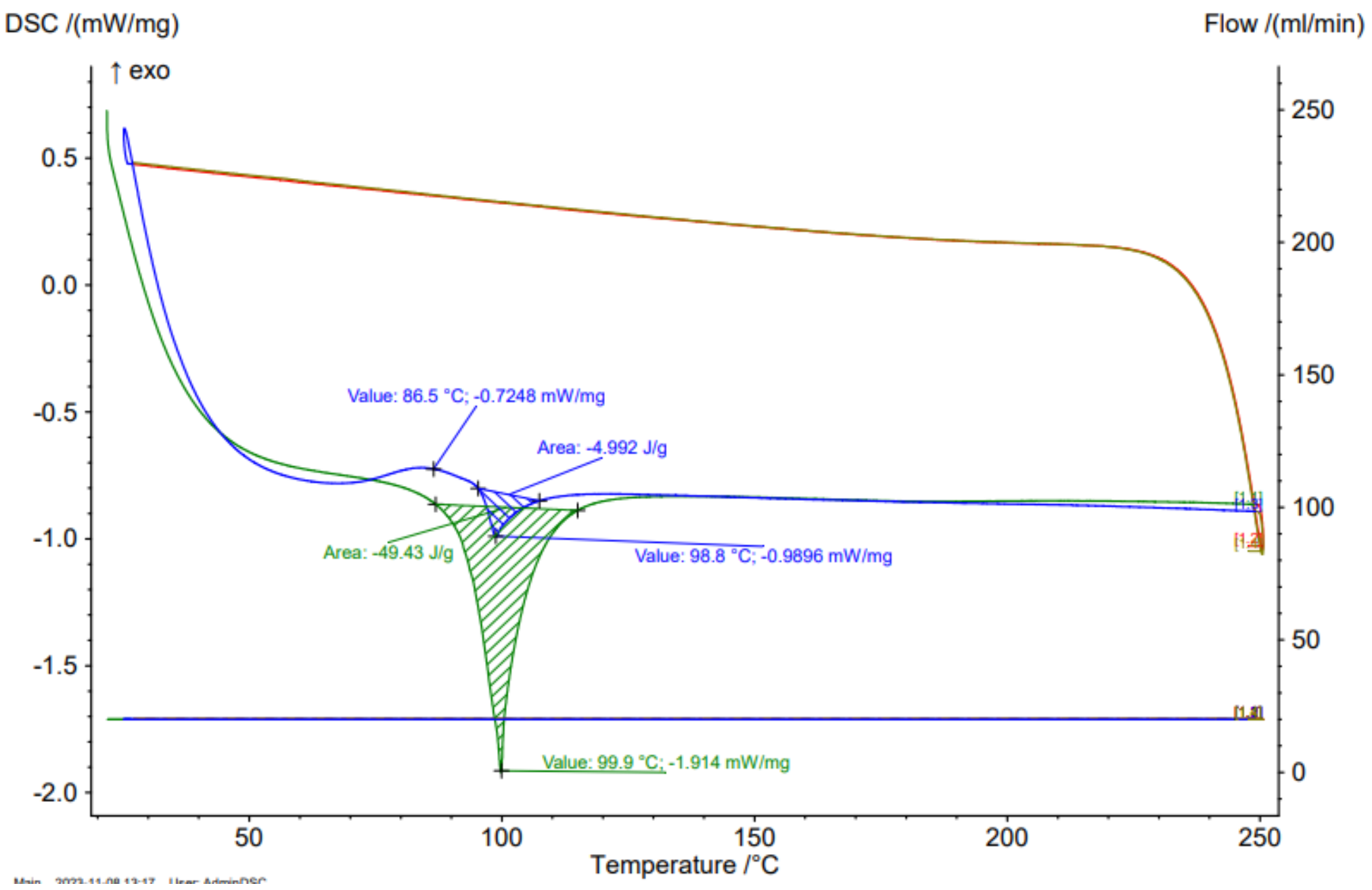

Figure S7. DSC of Flutolanil (**10**).

3-(Difluoromethyl)-1-methyl-*N*-(3′,4′,5′-trifluoro[1,1′-biphenyl]-2-yl)-1H-pyrazole-4-carboxamide (Fluxapyroxad) (11). CAS [907204-31-3].[11] Compound **11** was prepared according to:

- CDI-mediated synthesis in a planetary ball mill (Table 2, entries 7-9 and Table 3, entry 3):
  - *Method A3* (P7 Premium, Fritsch - Table 2):
    - *a)* entry 7 (reaction scale: 5.4 mmol): ZrO_2 jar (45 mL), with 50 ZrO_2 balls of 5 mm Ø (m_{ball} = 0.384 g – Table S1), milling at 500 rpm *Hz*; *step 1* was 60 min, *step 2* was 90 min. Yield: 3 % (55 mg);
    - *b)* entry 8 (reaction scale: 5.4 mmol): ZrO_2 jar (45 mL), with 57.1 g (648) ZrO_2 balls of 3 mm Ø (m_{ball} = 0.088 g – Table S1), milling at 500 rpm *Hz*; *step 1* was 60 min, *step 2* was 90 min. Yield: 6 % (120 mg);
    - *c)* entry 9 (reaction scale: 5.4 mmol): ZrO_2 jar (45 mL), with 57.1 g (648) ZrO_2 balls of 3 mm Ø (m_{ball} = 0.088 g – Table S1), and *N*-methylimidazole (NMI) as LAG solvent (517.6 µL , η = 0.16 µL/mg), milling at 500 rpm *Hz*; *step 1* was 60 min, *step 2* was 90 min. For the work-up: the precipitation was done by stirring the crude in 1M HCl solution instead of water to ensure proper solubilization of the starting amine and to eliminate any residual NMI. Yield: 23% (473 mg).
  - *Method A3* (P6 Fritsch - Table 3, entry 3):
    - *d)* entry 3 (reaction scale: 5.4 mmol): ZrO_2 jar (45 mL), with 57.1 g (648) ZrO_2 balls of 3 mm Ø (m_{ball} = 0.088 g – Table S1), and *N*-methylimidazole (NMI) as LAG solvent (517.6 µL, η = 0.16 µL/mg), milling at 500 rpm *Hz*; *step 1* was 60 min, *step 2* was 90 min. For the work-up: the precipitation was done by stirring the crude in 1M HCl solution instead of water to ensure

proper solubilization of the starting amine and to eliminate any residual NMI. Yield: 1% (14.3 mg).

- ODI-mediated synthesis in a planetary ball mill (Table 3, entry 3):
    - *Method B2* (reaction scale: 5.4 mmol):
        a) reaction performed in a P7P Fritsch: ZrO_2 jar (45 mL), with 57.1 g (648) ZrO_2 balls of 3 mm Ø (m_{ball} = 0.088 g – Table S1), and *N*-methylimidazole (NMI) as LAG solvent (631.3 μL, η = 0.16 µL/mg), milling at 500 rpm *Hz*; *step 1* was 90 min, *step 2* was 90 min. For the work-up: the precipitation was done by stirring the crude in 1M HCl solution instead of water to ensure proper solubilization of the starting amine and to eliminate any residual NMI. Yield: 66% (1.37 g).

        b) reaction performed in a P6 Fritsch: ZrO_2 jar (45 mL), with 57.1 g (648) ZrO_2 balls of 3 mm Ø (m_{ball} = 0.088 g – Table S1), and *N*-methylimidazole (NMI) as LAG solvent (631.3 μL, η = 0.16 µL/mg), milling at 500 rpm *Hz*; *step 1* was 90 min, *step 2* was 90 min. For the work-up: the precipitation was done by stirring the crude in 1M HCl solution instead of water to ensure proper solubilization of the starting amine and to eliminate any residual NMI. Yield: 53% (1.09 g).

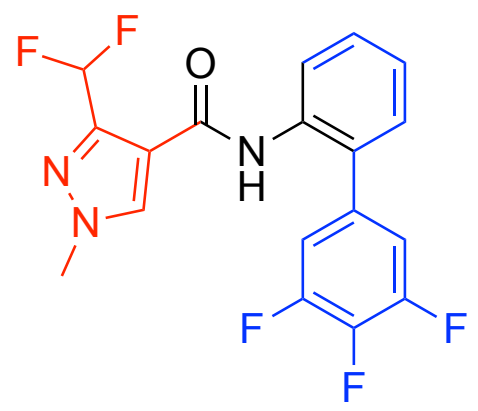

11

White powder, m.p. 157-158°C (capillary) (lit. 155°-158°C).[12] **^{1}H NMR** (400 M*Hz*, $CDCl_3$)[11] δ(ppm): 8.20 (d, *J* = 8.2 *Hz*, 1H), 7.96 (s, 1H), 7.81 (s, 1H), 7.46-7.41 (m, 1H), 7.23-7.22 (m, 2H), 7.02 – 6.98 (m, 2H), 6.64 (t, *J* = 53 Hz, 1H, CHF_2), 3.92z (s, 3H). **^{13}C{^{1}H}NMR** (101 M*Hz*, $CDCl_3$)[11] δ(ppm): 159.4, 151.4 (dd, J = 4.48 *Hz*, *J* = 10.16 *Hz*), 142.2, 138.3, 136.4, 134.6, 134.1 (t, *J* = 7.32 *Hz*), 131.2, 130.0, 129.3, 125.2, 123.4, 116.7, 113.9 (dd, *J* = 16.6, 4.6 *Hz*), 110.5 (t, *J* = 232.8 *Hz*), 39.6. **^{19}F{^{1}H}NMR** (376 M*Hz*, $CDCl_3$)[9] δ(ppm): -108.62 (s, 2F), -133.69 (d, *J* = 20.5 *Hz*, 2F), -161.48 (t, *J* = 20.5 *Hz*, 1F). **EI-(+)** *m/z*: 381 $[M]^{\bullet}$, 159 $[M-NH(C_{12}H_6F_3)]^{\bullet}$.

Table S3. ODI *vs* CDI as Coupling Agents.

Feature	1,1'-Oxalyl Diimidazole (ODI)	1,1-Carbonyldiimidazole (CDI)
**Structure**	Oxalyl moiety flanked by two imidazole rings	Carbonyl flanked by two imidazole rings
**Reactivity**	**Higher** — forms more reactive intermediates	Moderate — requires heating or long reaction times in some cases
**Mechanism**	Forms **acyl imidazolides** rapidly and releases **CO and CO_2**	Forms acyl imidazolides, releases **CO_2**
**Typical Applications**	Amide and ester formation under **mild conditions**, often in peptide synthesis	Broad use in ester, amide, and urea formation; less selective in some cases
**Reaction Conditions**	Works well at **room temperature**, no need for additives	Often needs **elevated temperature** or longer times
**Racemization**	**Low racemization** risk — good for peptide synthesis	Moderate racemization possible under some conditions
**Byproducts**	Imidazole + **CO + CO_2 → easy to remove, volatile**	Imidazole + **CO_2→ easy to remove, volatile**
**Selectivity**	More **selective** toward nucleophiles like amines	Less selective — alcohols, water, and amines can all react
**Side Reactions**	Fewer — no urea formation or O-acyl side products	Can lead to ***N*-acylureas** or ***O*-acylimidazoles** under some conditions
**Solvent Compatibility**	Good solubility in DCM, DMF, THF	Soluble in DCM, THF, DMF, ACN
**Cost & Availability**	Less common, more expensive	Widely available, cheaper

S3. Kinetic investigations.[13]

Extraction of kinetic curves from pressure data. In our setup, the pressure transducer records the overpressure inside the reactor, Δp, *i.e.* the internal pressure in excess of ambient pressure. This overpressure reflects the total amount of CO_2 produced during milling, which can be used as a kinetic marker once all non-reactive contributions are removed. Nevertheless, CO_2 may originate from two different processes: (i) the desired reaction leading to the formation of the *N*-acyl imidazole intermediate **1**, and (ii) a possible mechanical decomposition of CDI. Therefore, control milling experiments were performed first on CDI alone to quantify any background CO_2 evolution unrelated to the target reaction. The pressure profiles obtained for CDI were analysed considering that part of the pressure rise was induced solely by heating during milling. To isolate the chemical contribution associated with CO_2 formation, the thermal pressure contribution must be subtracted from the total measured pressure. Accordingly, the total pressure increase can be expressed as:

$$\Delta p_{CDI} = \Delta p_{th} + \Delta p_{ch,CDI} \quad \text{(Eq. 1)}$$

Here Δp_{th} is the thermal contribution, arising from gas expansion upon heating, and $\Delta p_{ch,CDI}$ is the chemical contribution, arising from the potential CO_2 generation due to CDI degradation. To estimate the number of moles of CO_2 produced, the measured pressure, Δp_{CDI}, was first corrected for the thermal contribution, Δp_{th}:

$$\Delta p_{ch,CDI} = \Delta p_{CDI} - \Delta p_{th} \quad \text{(Eq. 2)}$$

Δp_{th} is calculated assuming constant reactor volume and ideal gas behavior, with no gas generation occurring during heating:

$$\Delta p_{th} = p_{amb}\left(\frac{T}{T_{amb}}\right) - p_{amb} \quad \text{(Eq. 3)}$$

where p_{amb} is the ambient pressure, T is the measured absolute temperature inside the reactor, and T_{amb} is the ambient temperature. Once Δp_{ch} is known, the number of moles of CO_2, $n_{CO_2,CDI}$, generated can be estimated using the ideal gas law:

$$\mathrm{n}_{\mathrm{CO}_2,CDI} = \frac{\Delta p_{ch,CDI}\, V}{R\, T_{amb}} \quad \text{(Eq. 4)}$$

where $R = 83.14 \frac{L\, mbar}{mol\, K}$ is the gas constant, T_{amb} is the ambient temperature, and V is the free volume available to the gas inside the reactor. The free volume is calculated as:

$$V = V_{reactor} - \left(V_{balls} + V_{powder}\right) \quad \text{(Eq. 5)}$$

with

$$V_{balls} = n_b * V_b \quad \text{(Eq. 6)}$$

where $V_{reactor}$ is the internal volume of the milling jar, V_{balls} and V_{powder} are the volumes occupied by the balls and by the powders, respectively, n_b is the total number of balls inside the reactor, and V_b is the volume of a single ball. After estimating the number of moles of CO_2 generated during CDI milling, $\mathrm{n}_{\mathrm{CO}_2,CDI}$, and knowing the theoretical stoichiometric amount of CO_2 expected if all CDI is decomposed, $\mathrm{n}_{\mathrm{CO}_2,CDI,st}$ the molar fraction of CO_2 produced can be calculated as:

$$\alpha_{CO_2,CDI} = \frac{n_{CO_2,DCI}}{n_{CO_2,DCI,st}} \quad \text{(Eq. 7)}$$

by definition $\alpha_{CO_2,CDI}$ ranges from 0 to1. The time evolution of this quantity is reported in Figure 2.

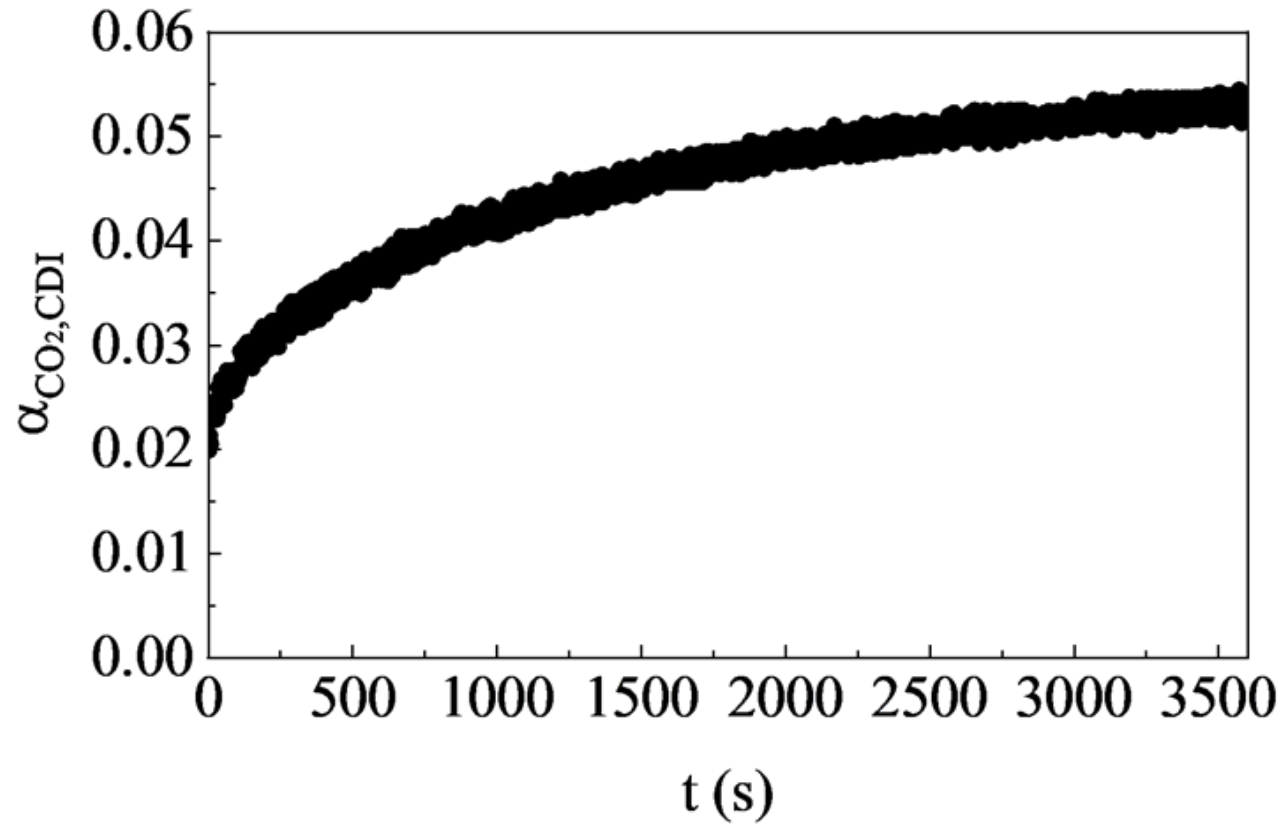

Figure S8. Molar fraction of CO_2 formed due to degradation, $\alpha_{CO_2,CDI}$, as a function of the milling time, t.

Data in Figure S8 indicate that the amount of CO_2 produced due to CDI degradation is negligible. Therefore, the pressure increase observed during the reaction experiments can be fully attributed to the CO_2 released in the first reaction step due to the formation of the *N*-acyl imidazole intermediate **1**. By applying Equation 4 to the measured pressure profiles of the reaction experiments, the time evolution of the molar fraction of CO_2 formed, α_{CO_2}, was obtained. The kinetic profiles are shown in Figure S9 and all exhibit a sigmoidal shape, although their slopes differ markedly.

In particular, the reaction involving Fluxapyroxad **11** proceeded significantly faster than that of Mepronil **9**, rapidly reaching high CO_2 conversion. The profile of Flutolanil **10** appears intermediate in the early stage but then deviates substantially from the other two systems. Notably, both Mepronil **9** and Flutolanil **10** displayed changes in slope after the early stages of the reaction, which is much more pronounced in the latter case. Because these two substrates exhibit comparable rheological behavior as powders during milling, such variations may partly arise from the evolving rheology of the system and, to a lesser extent, from limitations in the sensitivity of the pressure transducer.

To gain further insight into the origin of the slope changes, an additional experiment was performed on Flutolanil **10**. The milling was interrupted after 300 s, which corresponds to the milling time at which a marked change in the kinetic slope is observed, and then restarted after a 10-minutes pause. Since the pressure detector only works when the ball milling is operating, the recorded data consist of two separate datasets that can be properly combined to reconstruct the overall reaction kinetics, as shown in Figure S10.

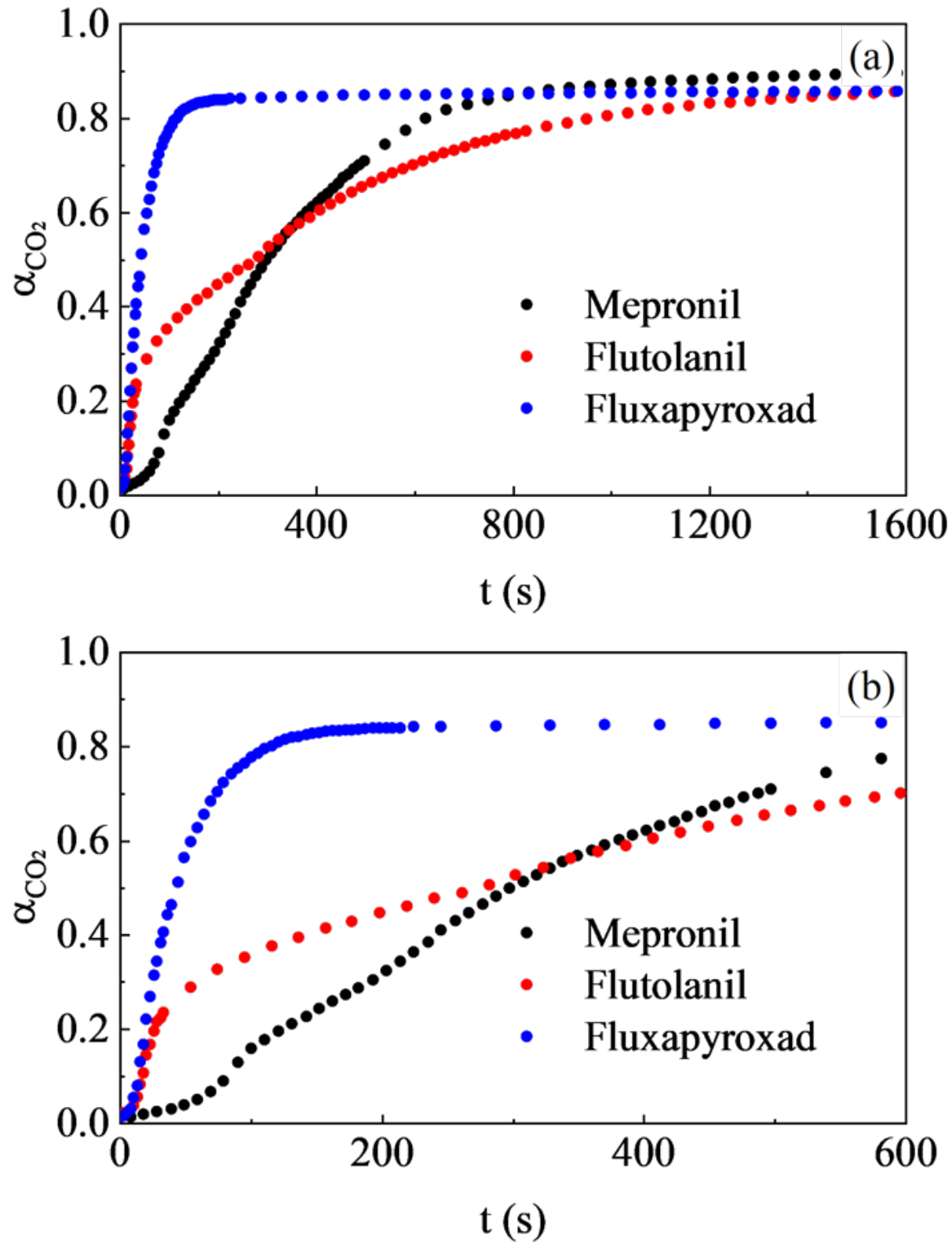

Figure S9. a) Molar fraction of CO_2 formed due to the formation of the *N*-acyl imidazole intermediate **1**, α_{CO_2}, as a function of the milling time, t; b) Magnification of a).

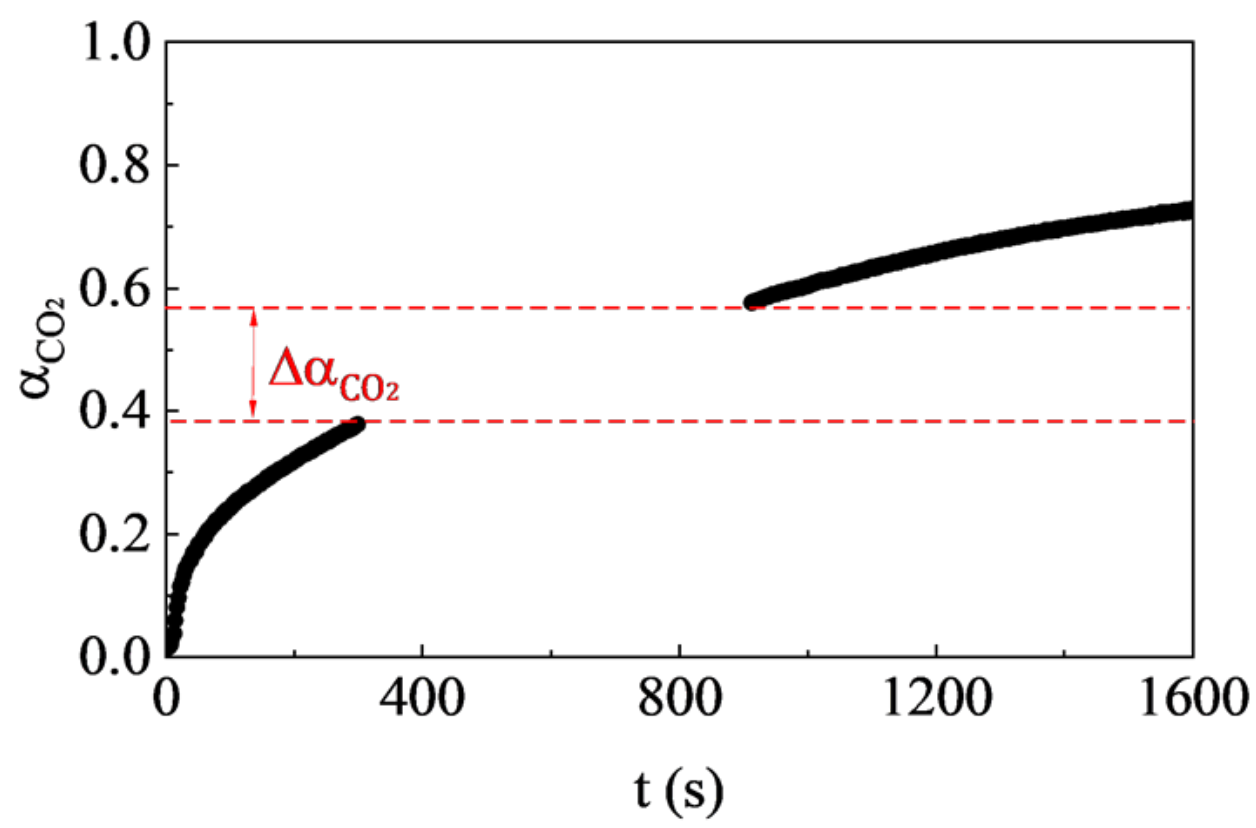

Figure S10. Molar fraction of CO_2 formed due to the formation of the *N*-acyl imidazole intermediate **1**, α_{CO_2}, as a function of the milling time, t.

Kinetic modelling. The kinetic curves can be rationalized by applying a model based on a statistical description of the mechanical processing of powders in ball mills. The model is based on the consideration that upon impact, mechanical stresses are generated at interparticle contacts within the powder volume trapped between the milling tools. When the local stress intensity exceeds the yield stress, plastic

deformation occurs. Under these conditions, so-called critical loading conditions (CLCs) can arise in small, irregularly distributed, volumes v^*. It is within volumes v^* that the reactant transforms into the product.

To a first approximation, the involvement of powders in impacts is stochastic. Therefore, volumes $v^*$are also affected by CLCs stochastically. It follows that volumes v^* that have been affected by CLCs a different number of times coexist within the total volume V of powder inside the reactor. Volumes v^* involved in a different number of CLCs can be expected to exhibit a different product molar fraction, α_i. Then, the global kinetics of the mechanically activated transformation can be satisfactorily rationalized only once local kinetics is combined with a suitable statistical framework. To this aim we assume that: (i) the vigorous stirring of the powders keeps the powder charge homogeneous throughout the milling process, (ii). at each impact, only a small volume $v = \sum v^*$ of the total powder inside the reactor, V, is subjected to critical loading conditions (CLCs) able to induce the transformation, (iii) the fraction of powder affected by CLCs, $\kappa = \frac{\sum v^*}{V}$ remains constant throughout the entire process and, (iv) the local transformation degree, α_i, in volume v changes with the number i of CLCs the volume experiences. The volume fraction of powder affected by CLCs i times after m impacts, $\chi_i(m)$, can be expressed as

$$\chi_i = \left[\frac{(\kappa m)^i}{i!}\right] \exp(-\kappa m) \quad \text{(Eq. 8)}$$

The global transformation degree, $\alpha(m)$, is equal to the sum of the local transformation degrees, α_i, weighted by the volume fractions $\chi_i(m)$ of powder that have experienced CLCs i times. Accordingly,

$$\alpha(m) = \sum_{i=1}^{\infty} \chi_i\,(m)\alpha_{P,i} \quad \text{(Eq. 9)}$$

Taking into account that the total number of impacts, m, can be simply expressed as the product $f\ t$ between impact frequency, f, and time, t, Equation 8 can be re-written in the time-dependent form:

$$\chi_i(t) = \left[(\kappa\ f\ t)^i/i!\right] exp(-\kappa\ f\ t)\ \left[(k\ t)^i/i!\right] exp(-k\ t) \quad \text{(Eq. 10)}$$

where k is the volume fraction of powder affected by CLCs per unit time. Equation 10 can be also re-written as:

$$\alpha(t) = \sum_{i=1}^{\infty} \chi_i\,(t)\alpha_{P,i} \quad \text{(Eq. 11)}$$

The evolution of the local transformation degree, $\alpha_{P,i}$ with the number i of CLCs depends on the physical and chemical nature of the system. In these specific cases, the two solid reactants are initially segregated, and reaction can only occur where fresh interfacial contact is generated. Therefore, the formation of new interface and effective mixing of the reactants due to the occurrence of CLCs are expected to play a crucial role in determining the reaction kinetics. We also assume that the chemical reaction at interface between a pair of suitably oriented molecules can take place with a probability $0 < \Pi < 1$.

Within each individual volume v^* affected by CLCs, the interface area between reactants is expected to increase from an initial value S_{in} to an intrinsic upper limit S_{fin}. It follows that, $\alpha_{P,i}$ can be expressed as (Equation 12):

$$\alpha_{P,i} = 1 - \{[S_{in}\, exp(r\, i) + (S_{fin} - S_{in})]/S_{fin}\}^{-\Pi S_{fin}/(r\, s_{ca}\, N_{in})} \text{ (Eq. 12)}$$

Equation 12 provides an analytical description of the gradual increase of the product molar fraction within the volume v^* as a function of the number of CLCs experienced.

where r is the interface generation rate, s_{ca} and N_{in} are the number of contacts between reactant molecules and the total number of molecules and i is the number of CLCs that affected the volume v^*.

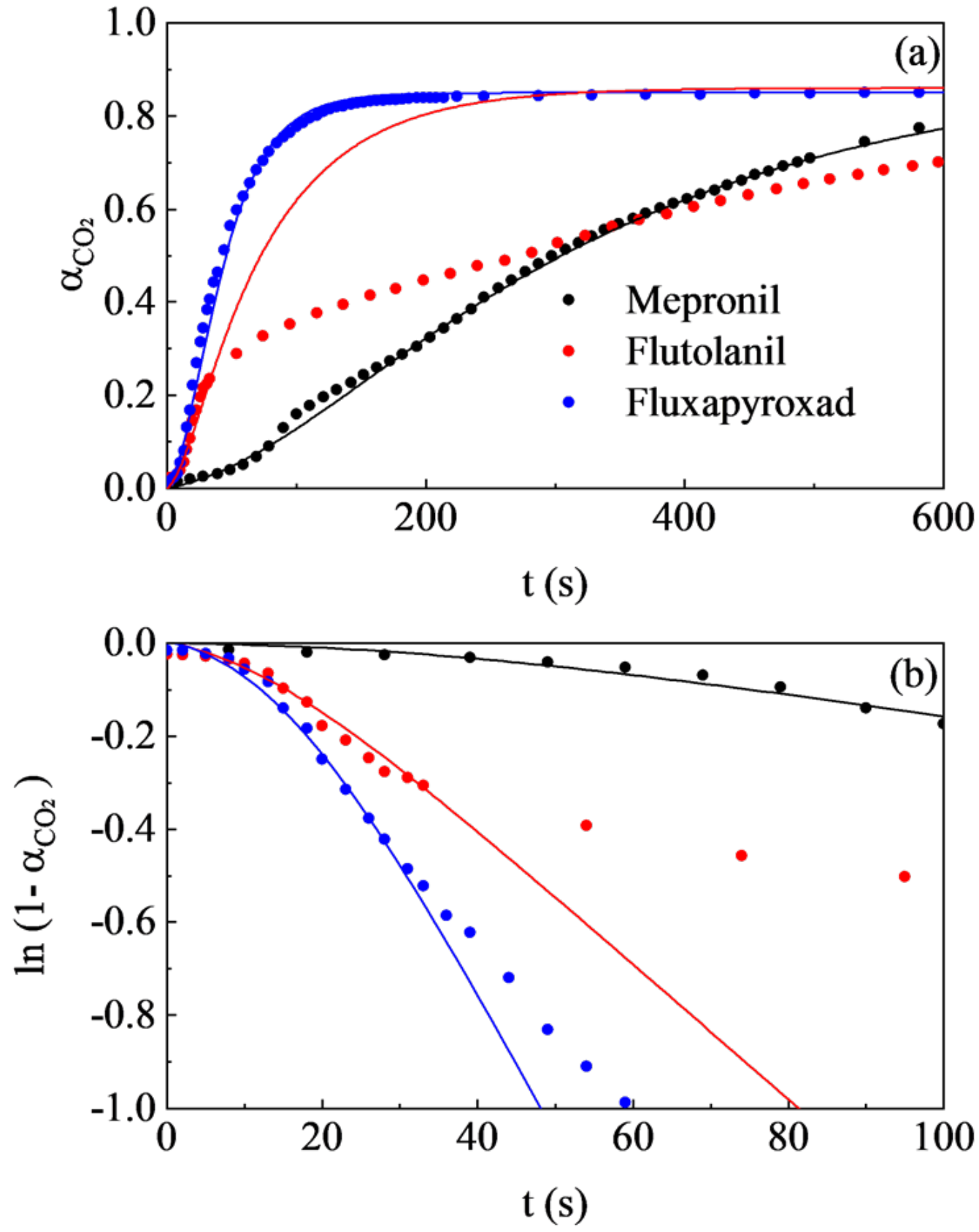

Figure S11. (a) Molar fraction of CO_2 formed due to the formation of the *N*-acyl imidazole intermediate **1**, α_{CO_2}, and (b) the logarithm of $1 - \alpha_{CO_2}$, as a function of the milling time, t, for Mepronil (**9**, black), Flutolanil (**10**, red), and Fluxapyroxad (**11**, blue). Solid lines represent fits obtained using Equation 11.

For clarity, the kinetic model described by Equation 11 accounts for three key parameters that can be interpreted as follows: (i) the deformational factor, r, *i.e.* the rate at which new interfacial area is generated within volumes affected by CLCs, reflecting the efficiency of forced mixing at decreasing length scales; (ii) the chemical factor Π, *i.e.* the probability that two reactant molecules in interfacial contact undergo chemical transformation, depending on the fulfilment of both steric and energetic requirements; (iii) the statistical factor, k, *i.e.* the volume fraction of powder subjected to CLCs during individual impacts, and thus describes the stochastic involvement of particles in mechanical activation.

In general, Equation 10 reproduces the overall transformation profiles of Mepronil **9** and Fluxapyroxad **11**, within the limits imposed by experimental uncertainty, Figure 11a. In contrast, the Flutolanil **10** profile exhibits a clear change in slope that cannot be captured by Equation 11. Consistent with the evidence shown

in Figure 10, this indicates that only the very early stage of the reaction is mechanochemical, whereas beyond the initial stage CO_2 evolution becomes progressively less dependent on mechanical activation. While the later-stage profile of Flutolanil **11** could be reproduced by introducing additional fitting flexibility, for example by allowing time-dependent changes in the interfacial area generation during milling, such extensions would require a dedicated experimental and modelling effort and are therefore beyond the scope of the present study. For this reason, the kinetic analysis is restricted to the early stage of the reaction, where mechanical control is experimentally supported, and is shown on a semi-logarithmic scale in Figure 11b.

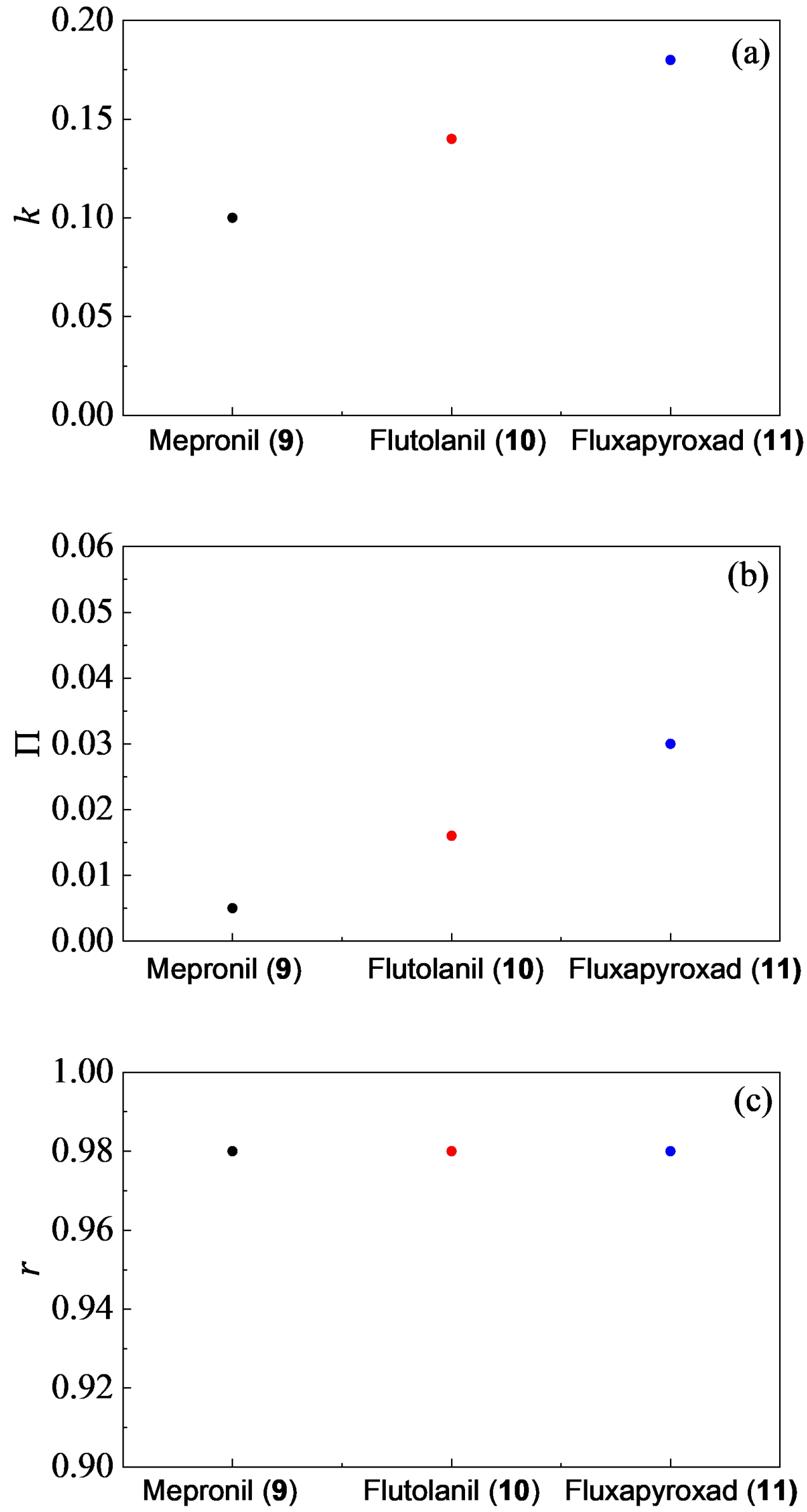

Figure S12. Comparison of fitting parameters obtained from the best fit of kinetic data, Figure 5b. (a) The fraction of powder affected by CLCs per unit time, k, (b) the reaction probability, Π, and (c) the interface area generation rate, r, for Mepronil **9** (black), Flutolanil **10** (red), and Fluxapyroxad **11** (blue).

The parameter values extracted from fitting the experimental data in Figure S11 with Equation 11 allow gaining some insight into the mechanochemical behavior of the different systems. A comparison of these

parameters is reported in Figure S12. It can be seen that the rate of interface generation r remains essentially constant and does not depend on the substrate. This indicates that, at least in the early mechanochemical stage, the generation of fresh interfacial contacts proceeds at a similar rate for Mepronil **9**, Flutolanil **10**, and Fluxapyroxad **11**, consistent with the fact that, in all cases, the reactants are initially present as fully segregated solid phases.

In contrast, the fraction of powder affected by CLCs per unit time, k, and the reaction probability Π vary depending on the substrate. In both cases, is observed the trend Fluxapyroxad **11** > Flutolanil **10** > Mepronil **9**. This means that, for Fluxapyroxad **11**, a larger fraction of the powder is subjected to CLCs per unit time. The intrinsic likelihood of chemical transformation follows a similar trend.

S4. Effect of technical parameter and difference of different models of planetary ball mills in the ODI-mediated synthesis of 9-11.

Table S4. Comparison of technical parameters of planetary ball-mills: P6 and P7P at a same sun disc speed (*i.e.*, 500 rpm).[a]

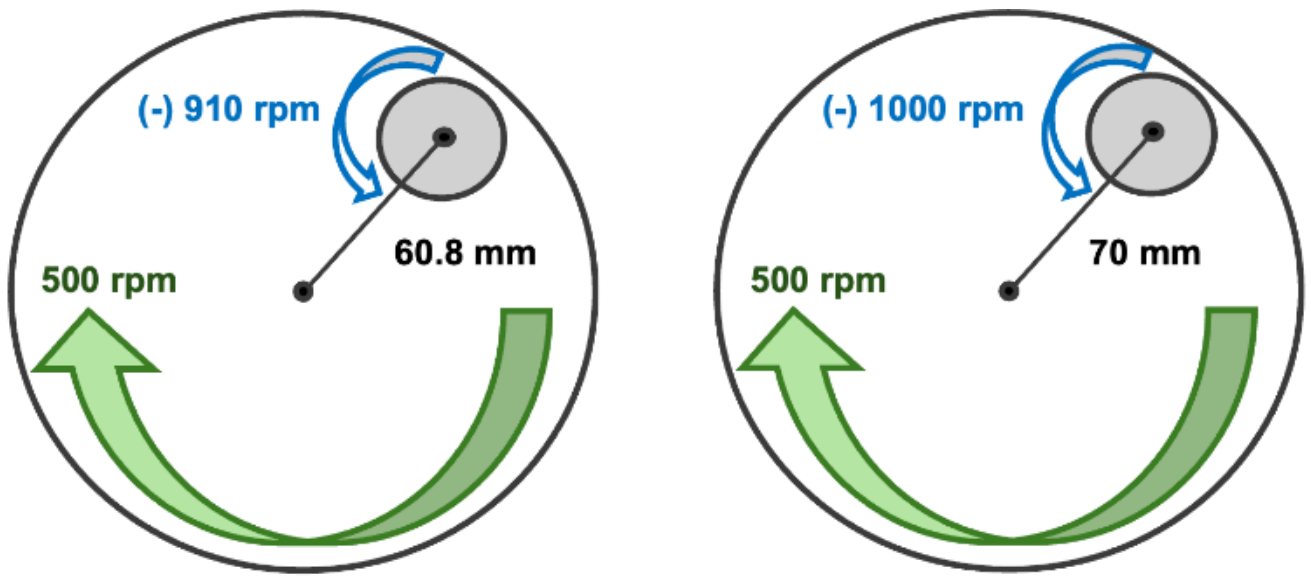

P6 Discs ratio: 1: -1.82 **P7P Discs ratio**: 1: -2

Technical characteristics	P6	P7P
r (mm)	60.8	70.0
^{i} relativ	1: -1.82	1:2
Acceleration (G)	17.12	19.65
Acceleration (m · sec^{-2}) [a]	167.94	192.77

[a] The conversion factor was the value of the force of gravity (9.81 m · sec^{-2}).

S4. Green chemistry metrics

Calculations on green metrics for the solution-based synthesis of the marketed agrochemicals Mepronil (**9**),[7] Flutolanil (**10**)[9, 14] and Fluxapyroxad (**11**)[11] were performed using the experimental data reported in literature. The solution-based protocols selected for the calculations were selected mainly on two criteria: i) the ones with best yields, and ii) the ones that were more closely to the mechanochemical methods herein developed (*i.e.*, considering solvent-based protocols involving the formation of an activated acid, followed by its reaction with an amine, performed either in a single or two steps). The best results for both the CDI- and the ODI-mediated mechanochemical amidation reactions to **9-11** in planetary ball-mills were benchmarked against solution-based protocols. The detailed calculations for both CHEM21 and DOZN™ are available as a separate excel file, as integrant part of the SI.

References

[1] D. C. Braddock, P. D. Lickiss, B. C. Rowley, D. Pugh, T. Purnomo, G. Santhakumar, S. J. Fussell, *Org. Lett.* **2018**, *20*, 950-953.

[2] B. F. Cain, *J. Org. Chem.* **1976**, *41*, 2029-2031.

[3] S. Mkrtchyan, M. Jakubczyk, S. Lanka, M. Yar, K. Ayub, M. Shkoor, M. Pittelkow, V. O. Iaroshenko, *Adv. Synth. Catal.* **2021**, *363*, 5448-5460.

[4] J. Liu, Q. Liu, H. Yi, C. Qin, R. Bai, X. Qi, Y. Lan, A. Lei, *Angew. Chem. Int. Ed.* **2014**, *53*, 502-506.

[5] P. Purushottamachar, A. Khandelwal, T. S. Vasaitis, R. D. Bruno, L. K. Gediya, V. C. O. Njar, *Bioorg. Med. Chem.* **2008**, *16*, 3519-3529.

[6] B. Picard, T. Fukuyama, I. Ryu, *J. Org. Chem.* **2023**, *88*, 5220-5225.

[7] I. Chiyomaru, S. Kawada, K. Takita, **1976**, *US3937840A*.

[8] S.-P. Wang, C. W. Cheung, J.-A. Ma, *J. Org. Chem.* **2019**, *84*, 13922-13934. .

[9] K. Yabutani, K. Ikeda, S. Hatta, T. Harada, *US4093743A* **1977**.

[10] Y. Wang, B. Tian, Y. Li, W. Li, Z. Chen, S. Liu, S. Li, *ChemSusChem* **2024**, *17*, e202301324.

[11] Y. Zhang, Z. Chen, J. Nie, F.-G. Zhang, J.-A. Ma, *J. Org. Chem.* **2019**, *84*, 7148-7158.

[12] U. Troppmann, W. Mayer, S. Koltzenburg, R. Israels, A. Bauder, U. Schlotterbeck, **2009**, *US8735321B2*.

[13] L. Vugrin, M. Carta, S. Lukin, E. Meštrović, F. Delogu, I. Halasz, *Faraday Discuss.* **2023**, *241*, 217–229.

[14] R. I. Patel, B. Saxena, A. Sharma, *Green Chem.* **2024**, *26*, 10265-10274.

^{1}H NMR in DMSO-d_6

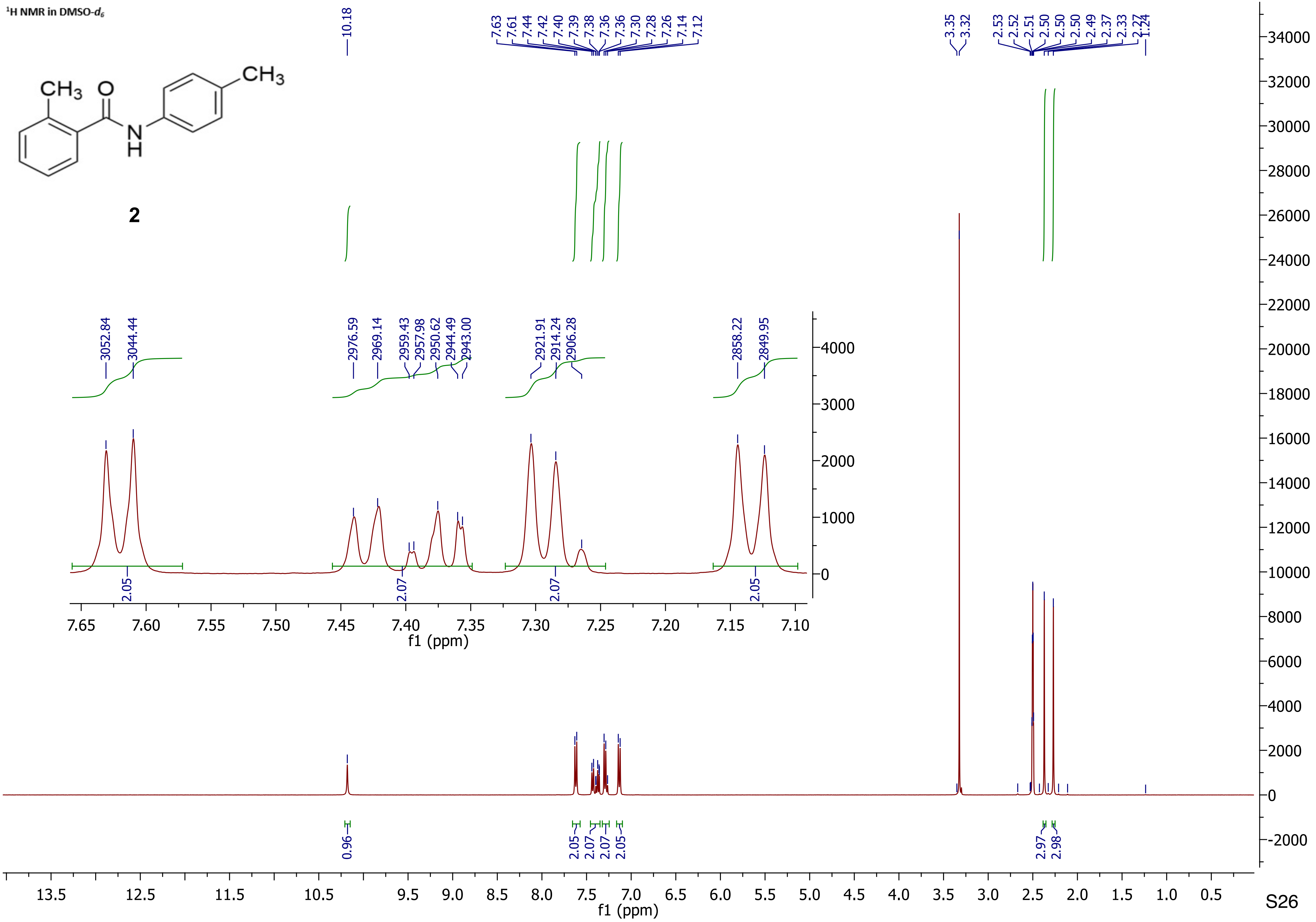

2

$^{13}C\{^1H\}$ NMR in DMSO-d_6

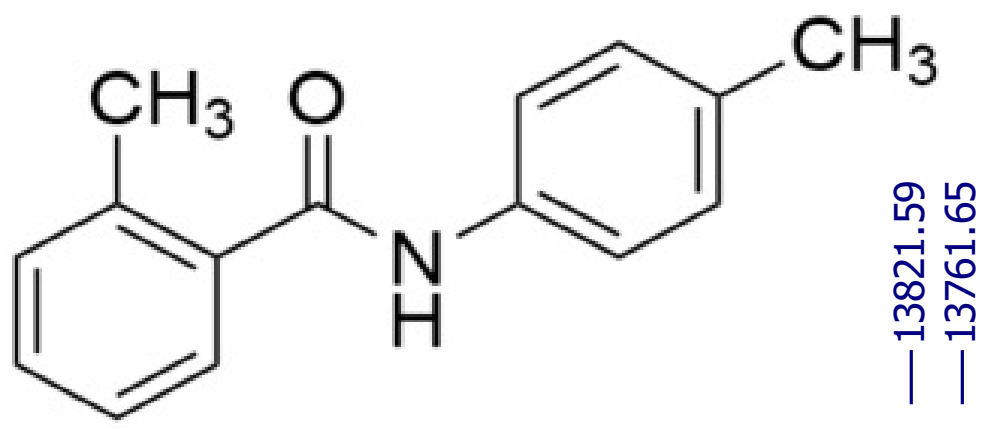

2

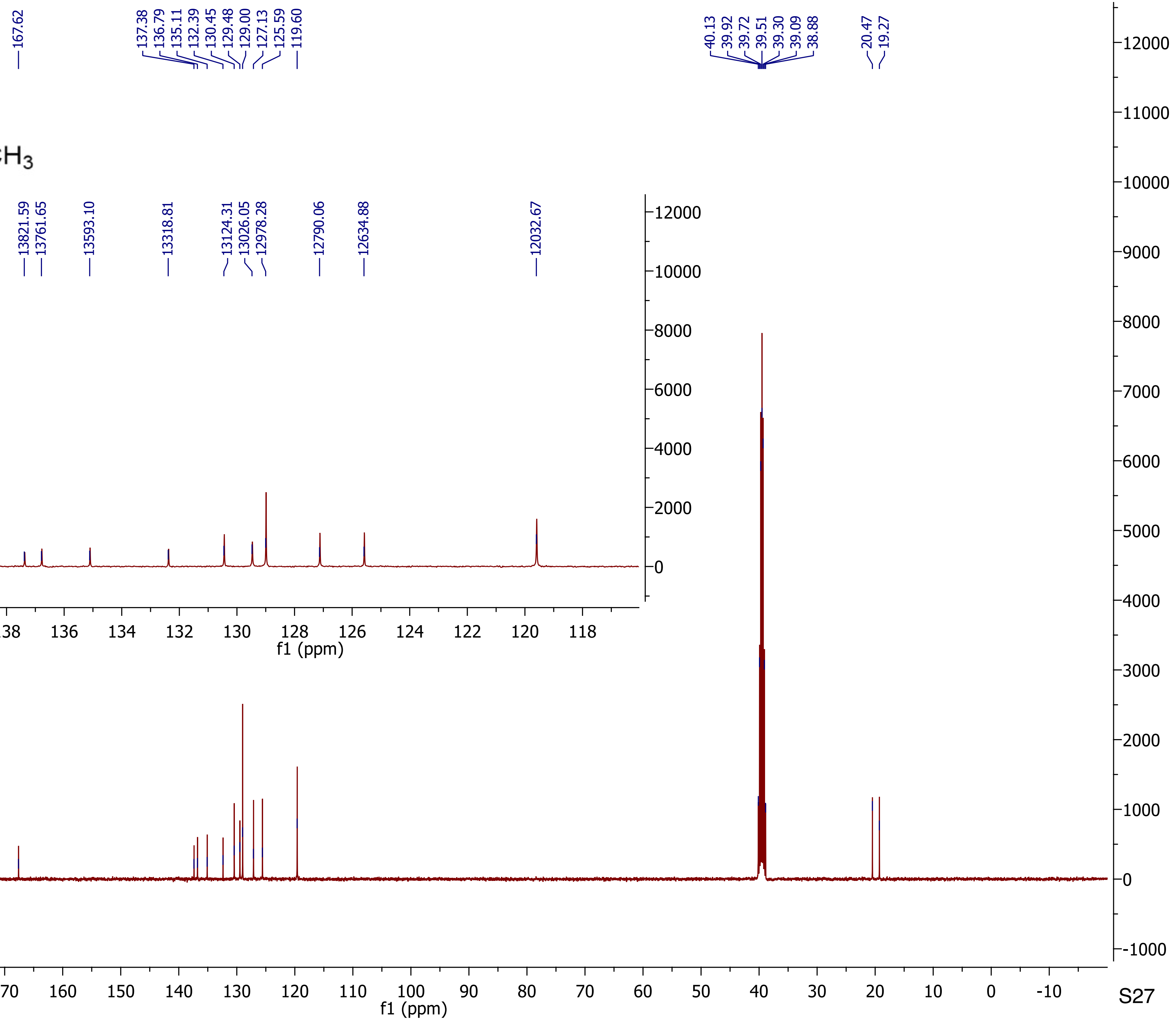

^1H NMR in DMSO-d_6

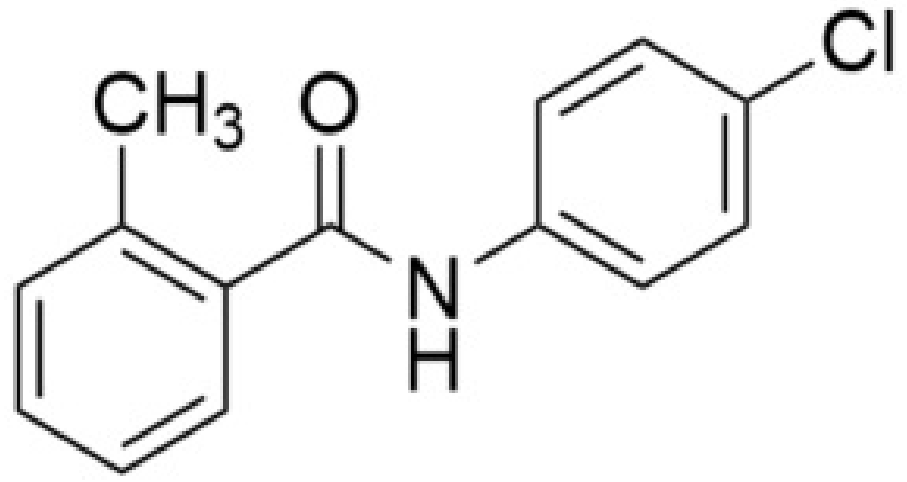

3

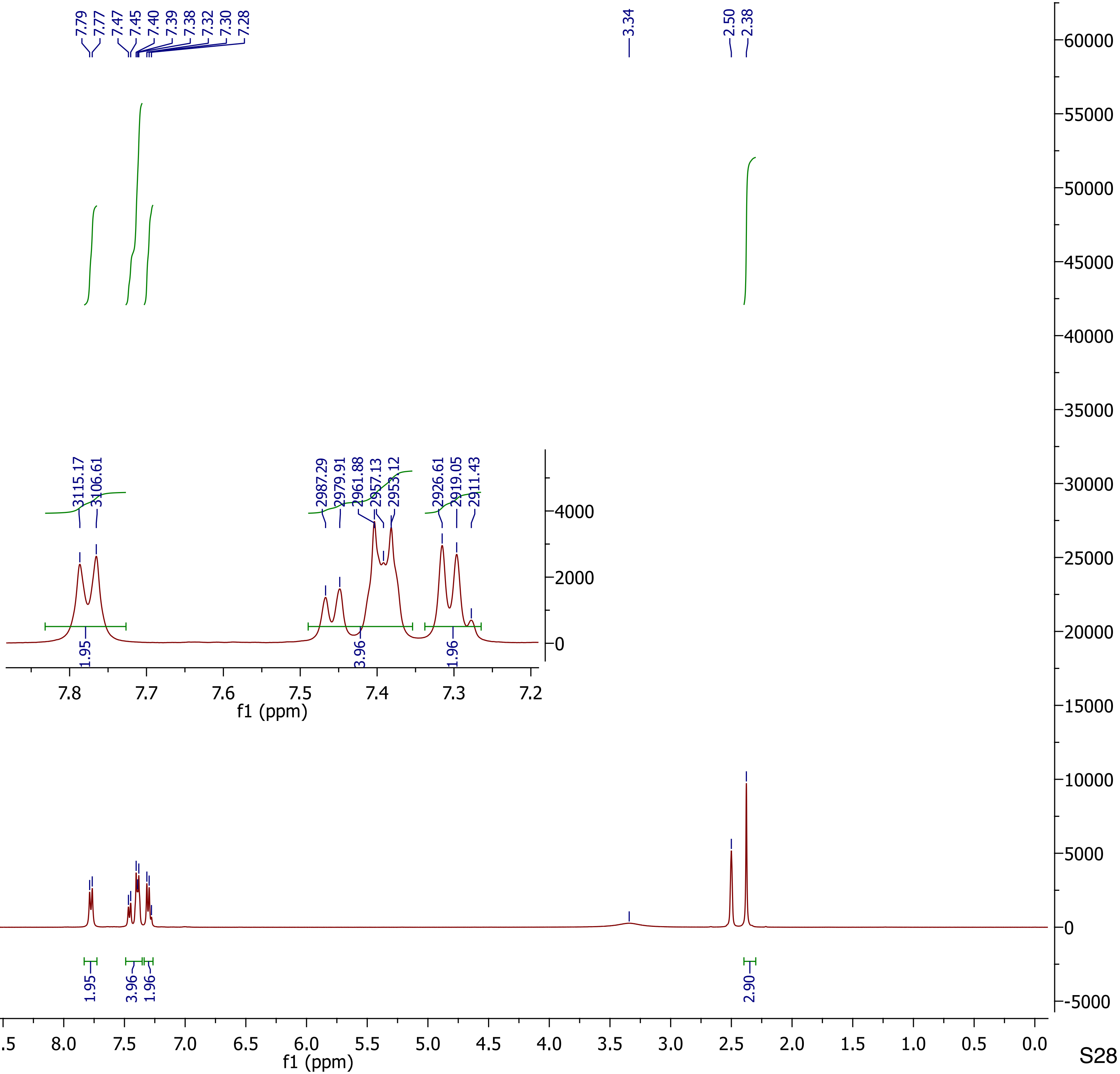

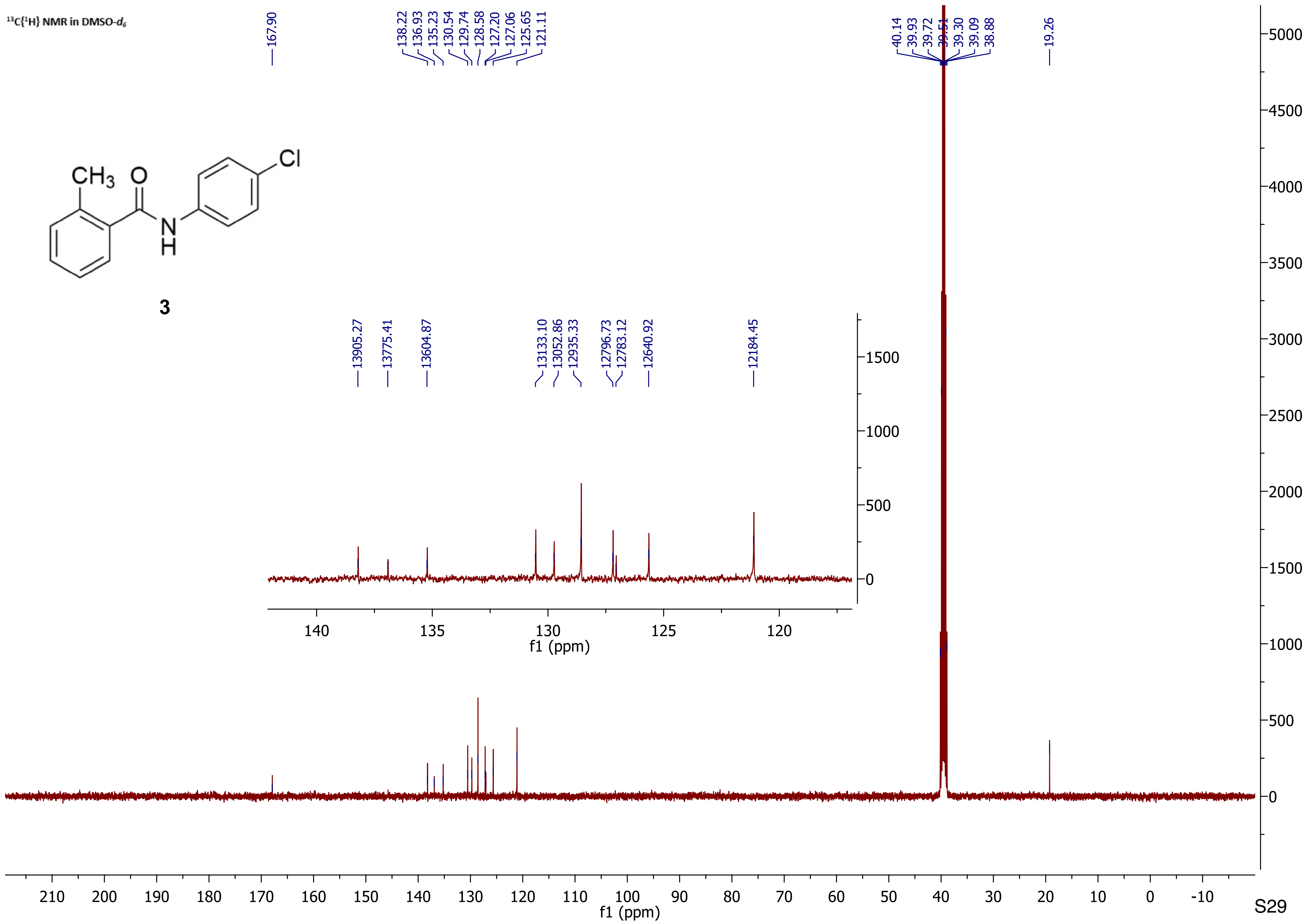

13C{1H} NMR in DMSO-d6
167.90
138.22
136.93
135.23
130.54
129.74
128.58
127.20
127.06
125.65
121.11
40.14
39.93
39.72
39.51
39.30
39.09
38.88
19.26
CH3
O
N
H
Cl
3
13905.27
13775.41
13604.87
13133.10
13052.86
12935.33
12796.73
12783.12
12640.92
12184.45
f1 (ppm)
f1 (ppm)

^{1}H NMR in DMSO-d_6

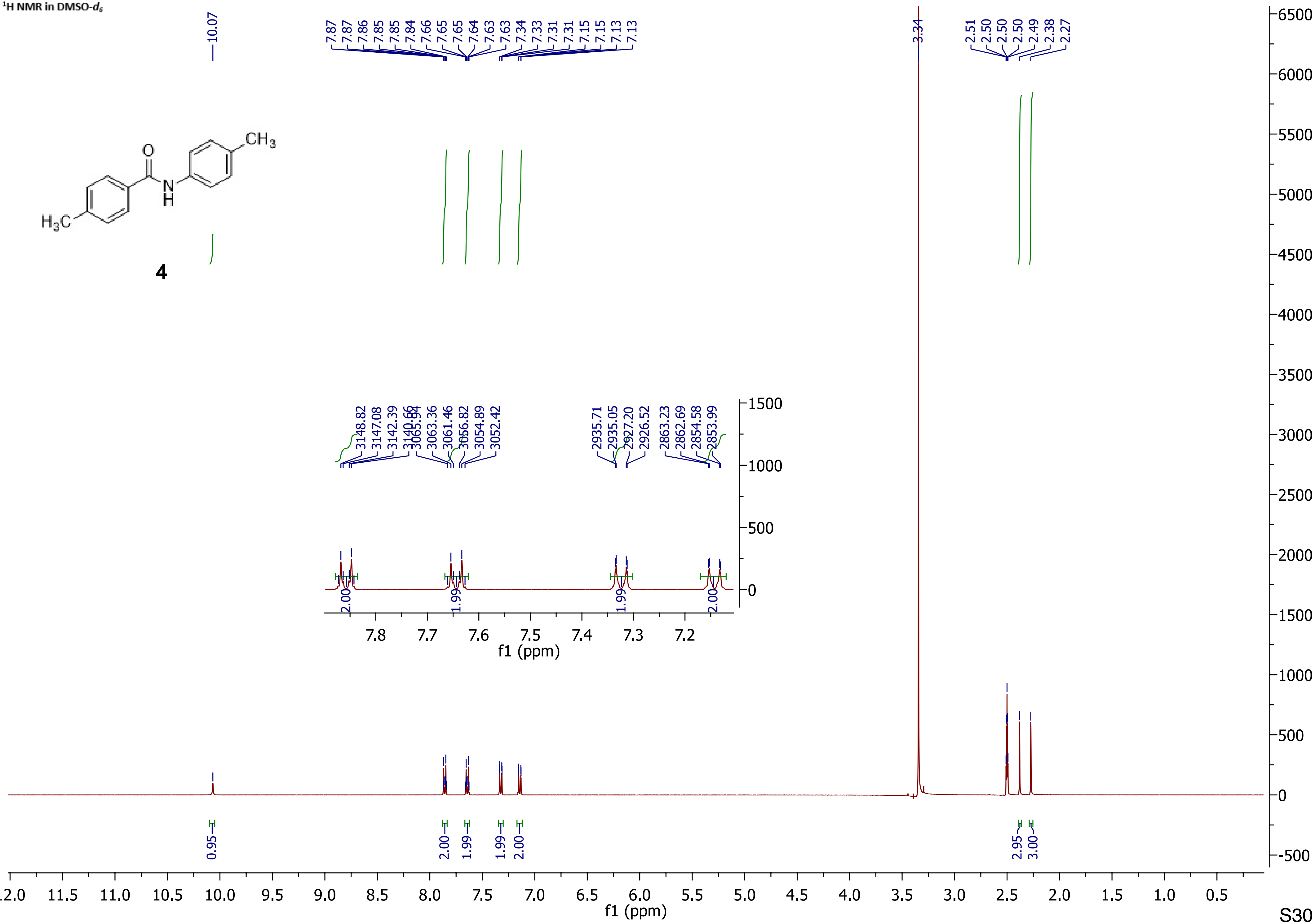

4

$^{13}C\{^1H\}$ NMR in DMSO-d_6

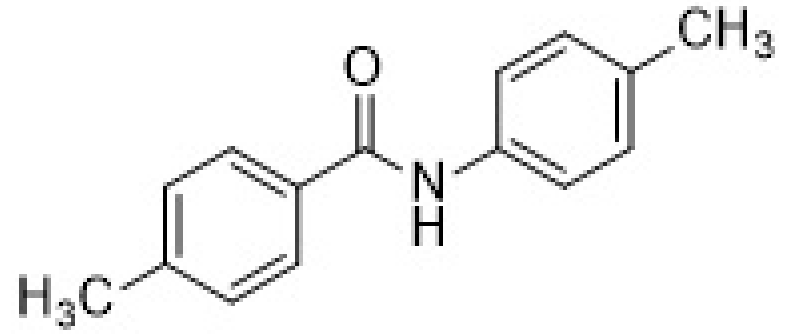

4

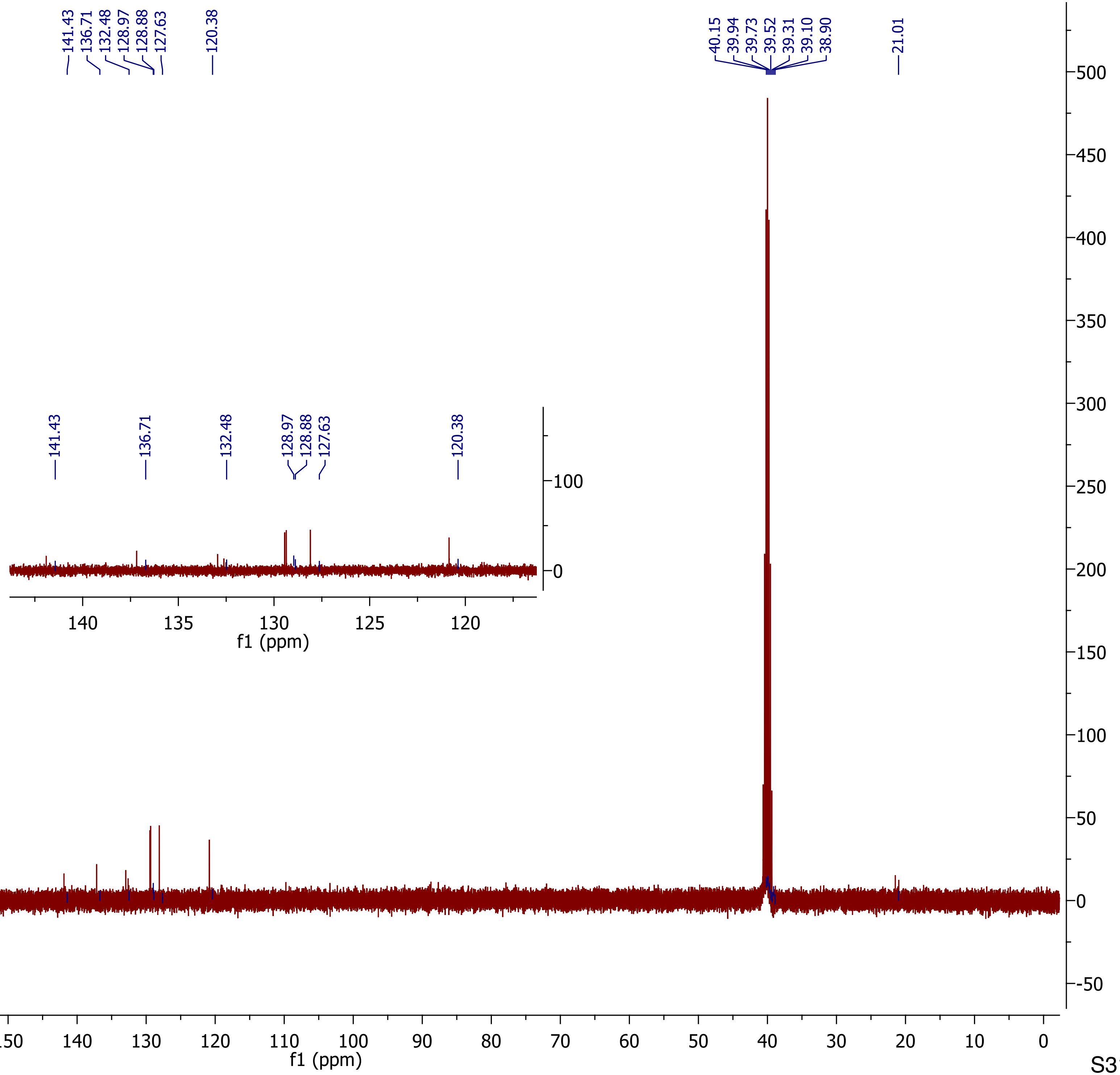

^{1}H NMR in DMSO-d_6

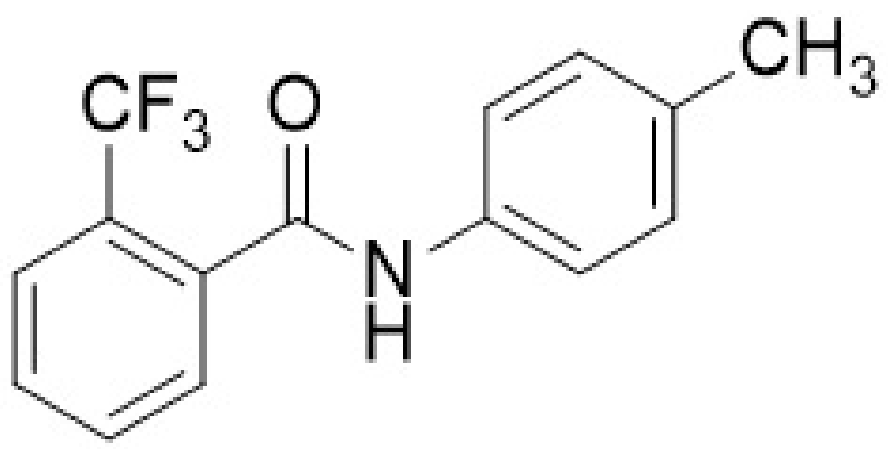

5

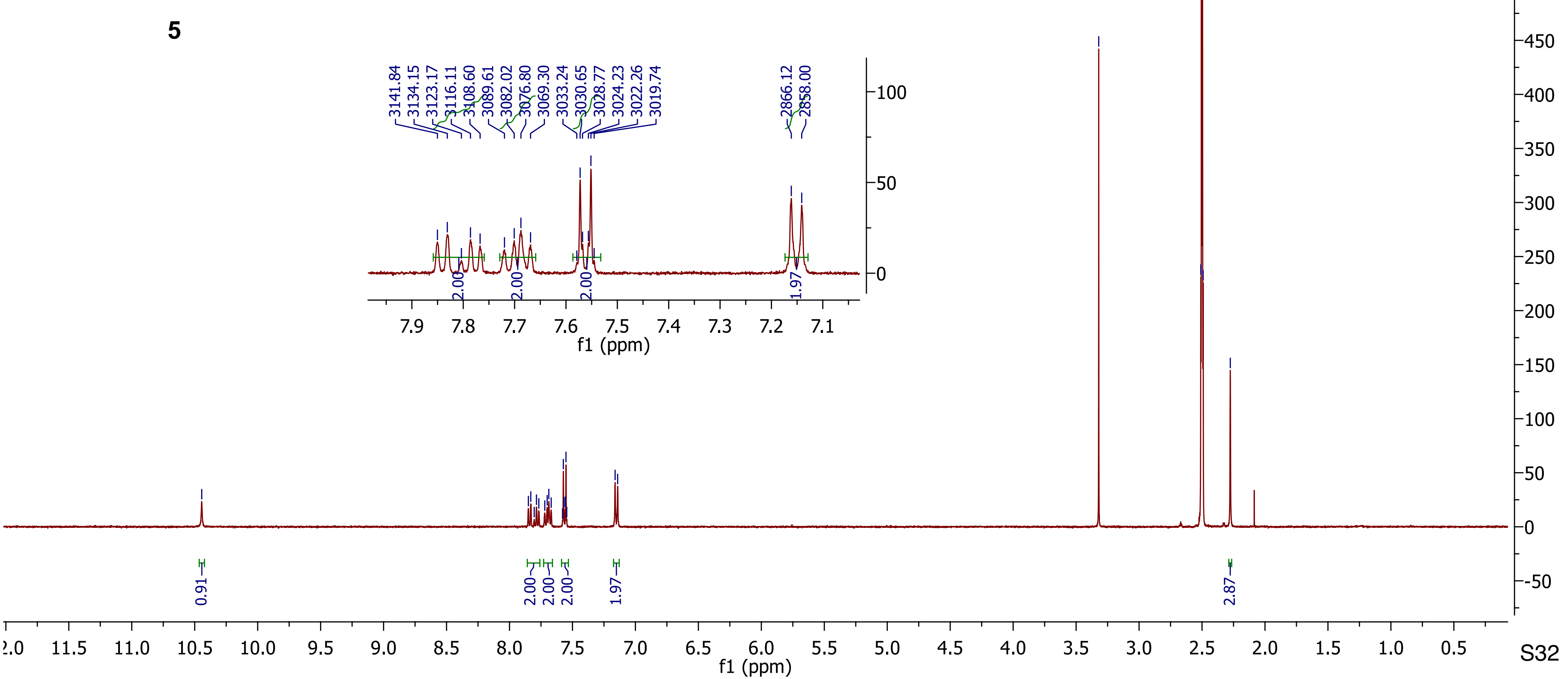

$^{13}C\{^1H\}$ NMR in DMSO-d_6

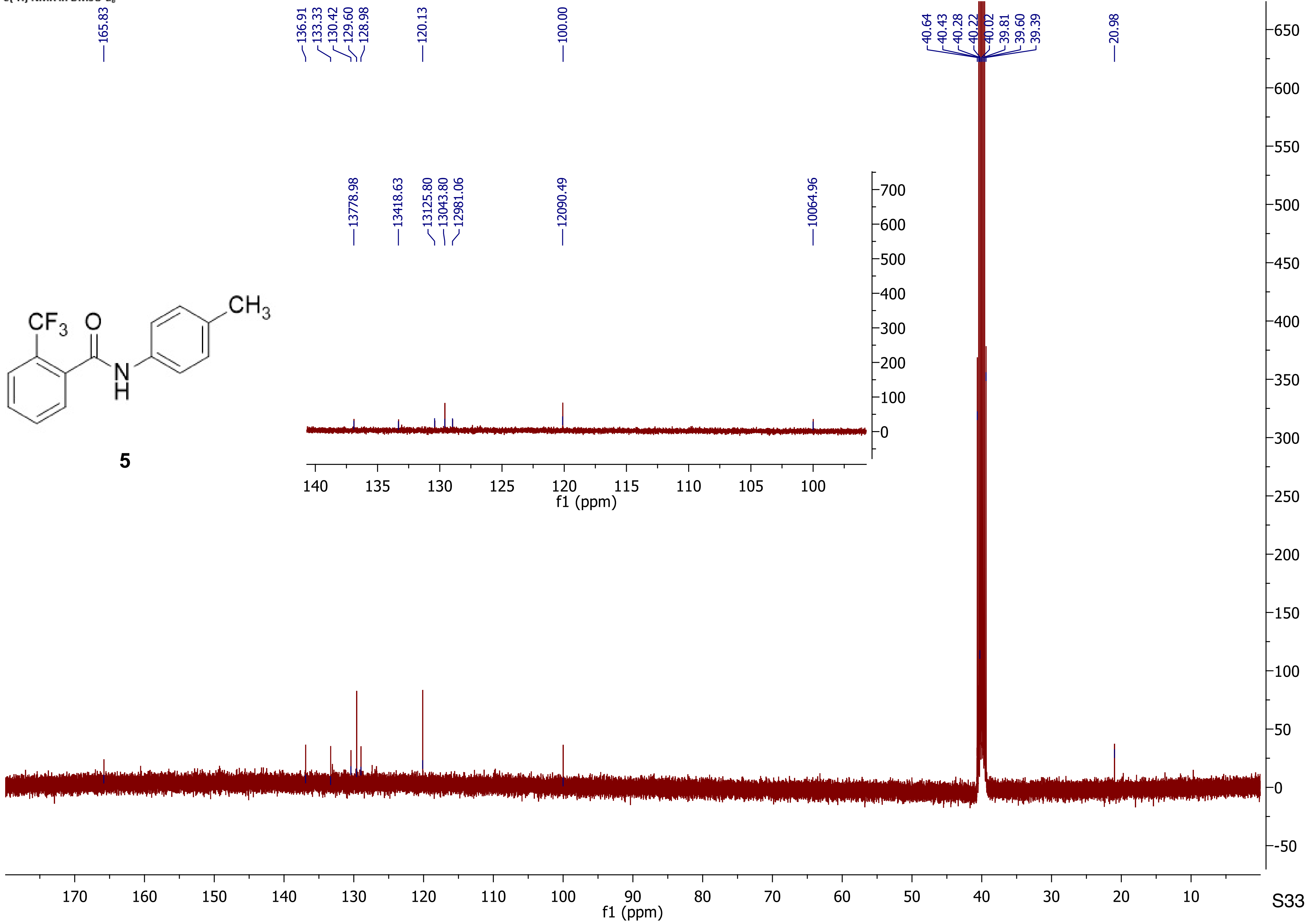

5

$^{19}F\{^1H\}$ NMR in DMSO-d_6

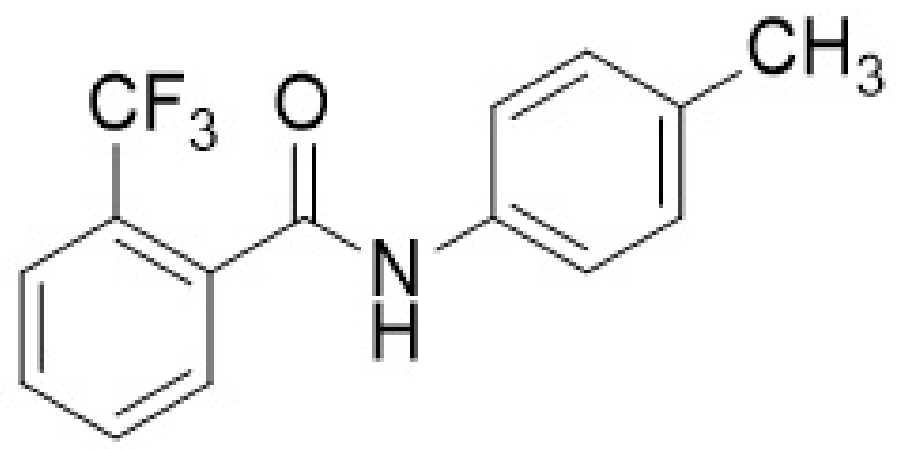

5

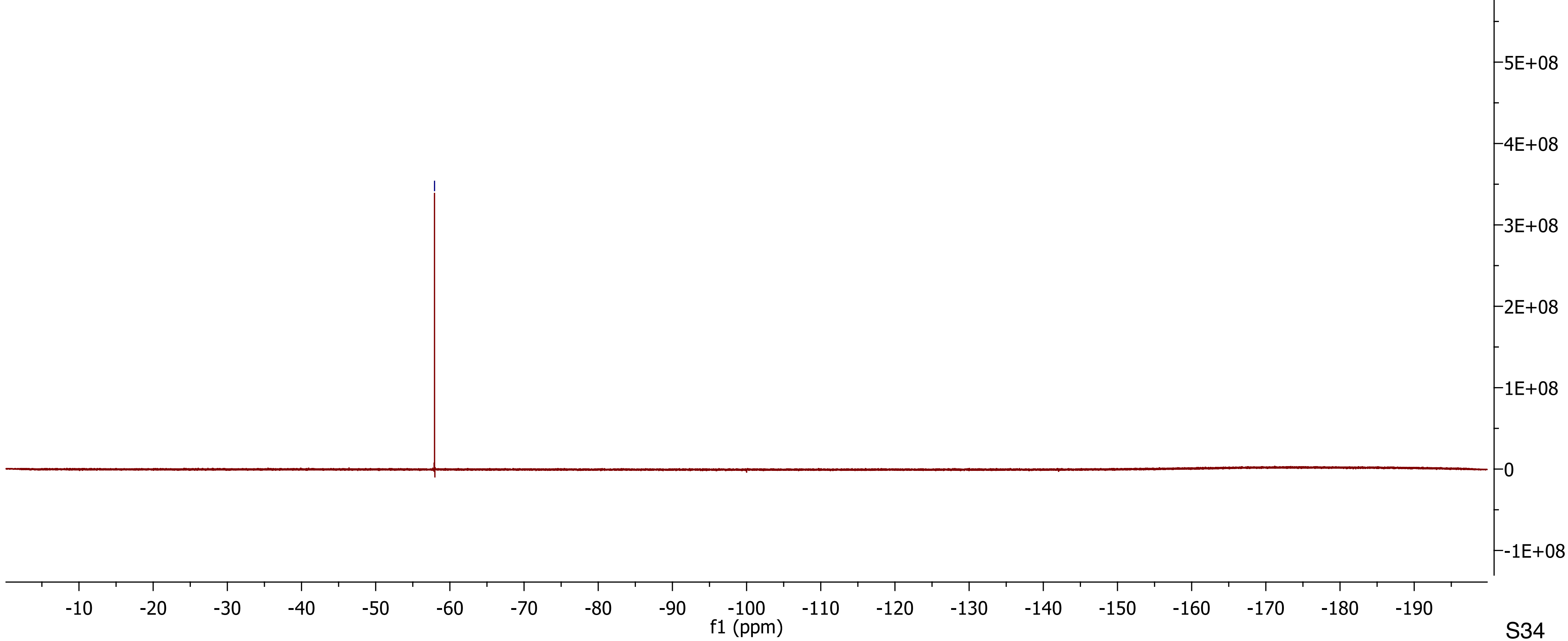

¹H NMR in DMSO-d_6

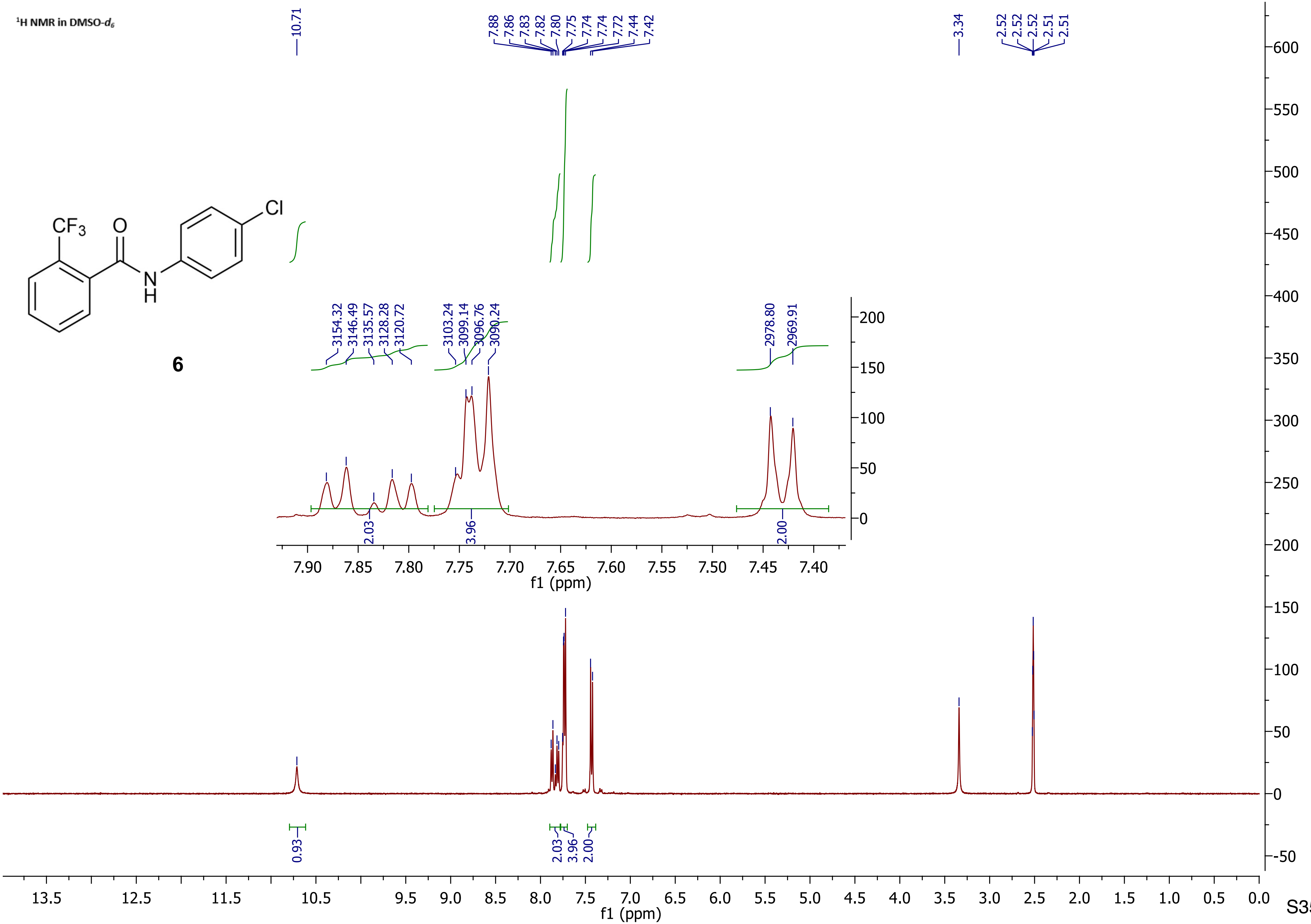

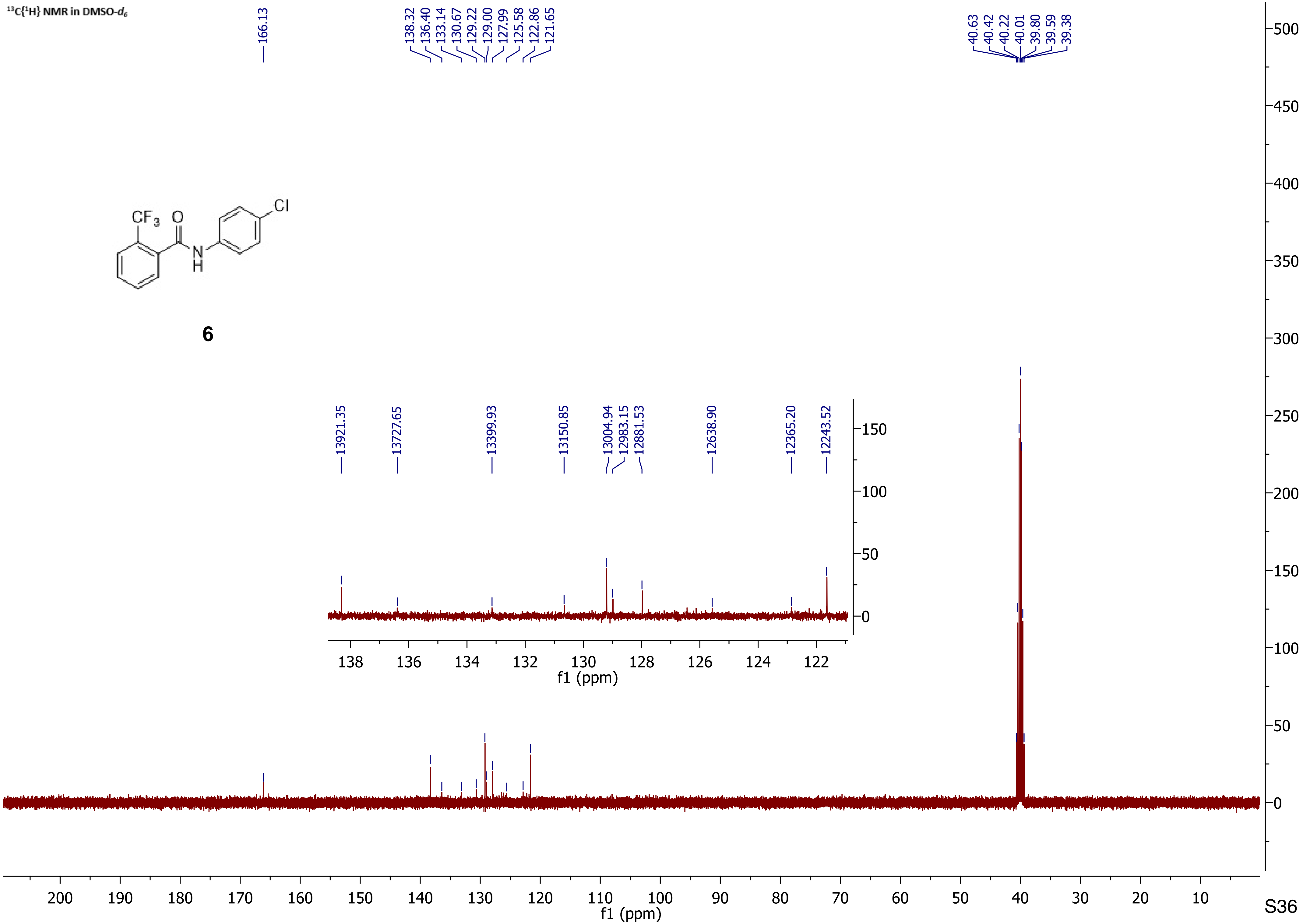

13C{1H} NMR in DMSO-d6
166.13
138.32
136.40
133.14
130.67
129.22
129.00
127.99
125.58
122.86
121.65
40.63
40.42
40.22
40.01
39.80
39.59
39.38
CF3
O
N
H
Cl
6
13921.35
13727.65
13399.93
13150.85
13004.94
12983.15
12881.53
12638.90
12365.20
12243.52
f1 (ppm)
f1 (ppm)

$^{19}F\{^1H\}$ NMR in DMSO-d_6

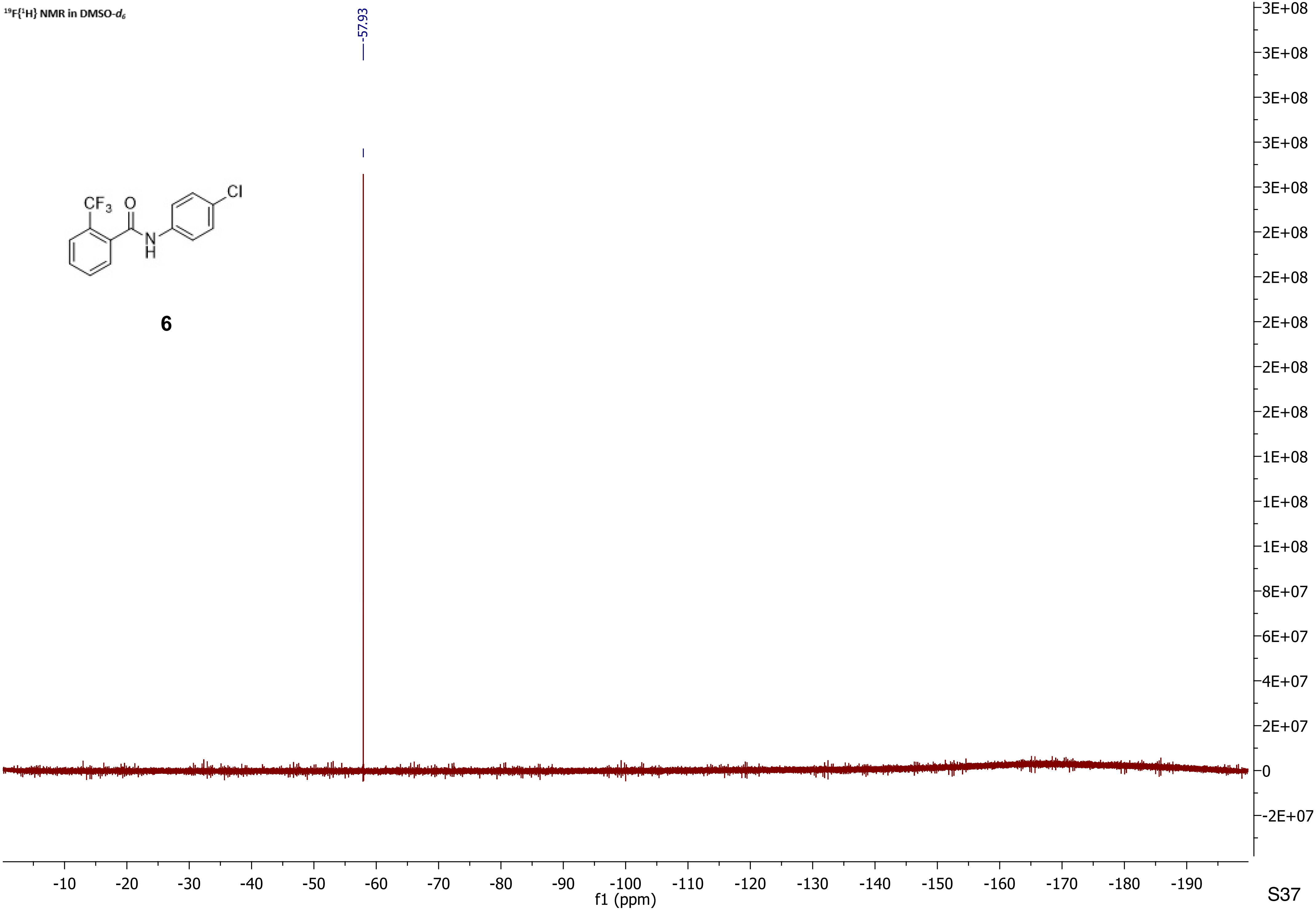

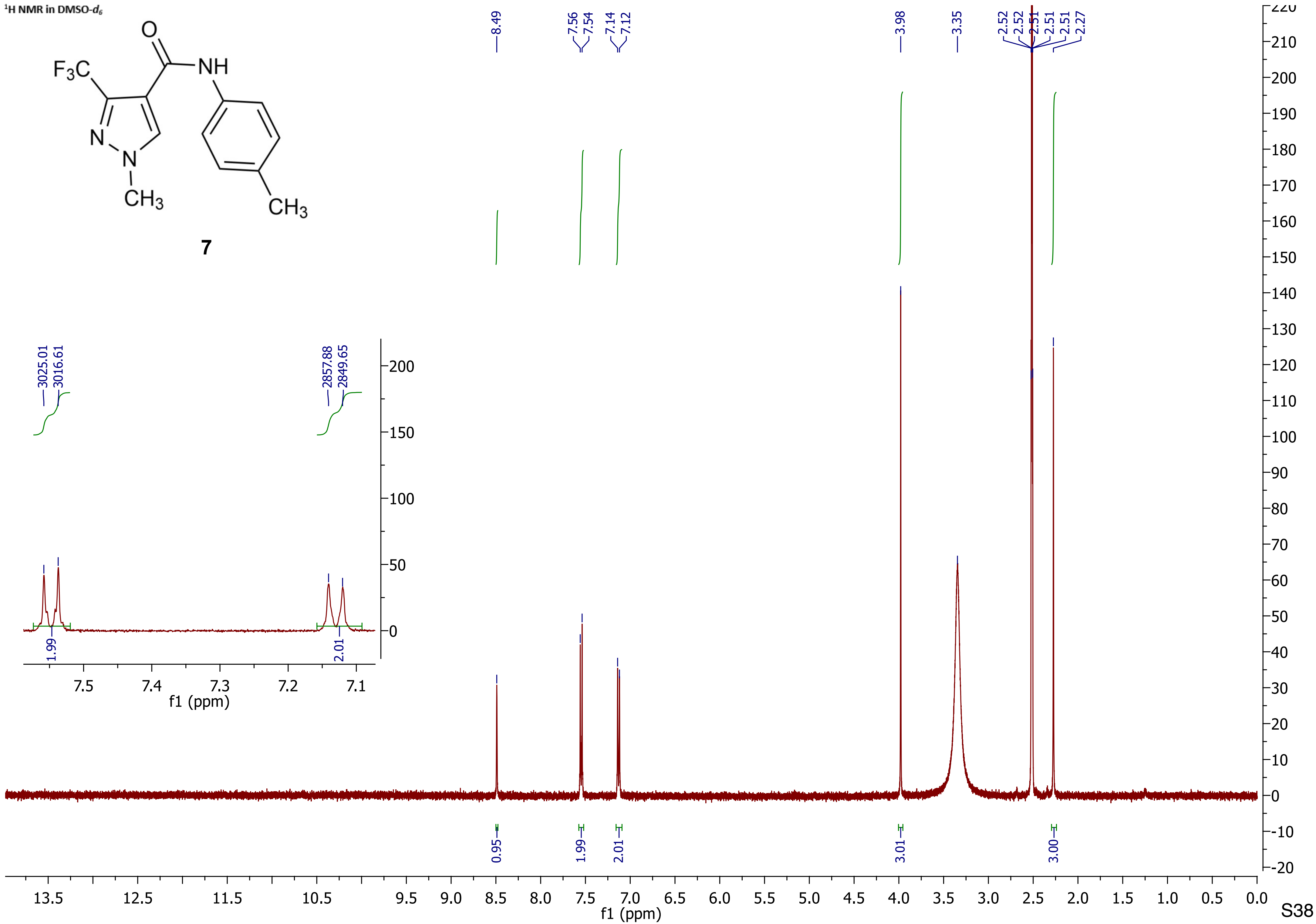

1H NMR in DMSO-d6
7
8.49
7.56
7.54
7.14
7.12
3.98
3.35
2.52
2.52
2.51
2.51
2.51
2.27
0.95
1.99
2.01
3.01
3.00
3025.01
3016.61
2857.88
2849.65
f1 (ppm)

$^{13}C\{^1H\}$ NMR in DMSO-d_6

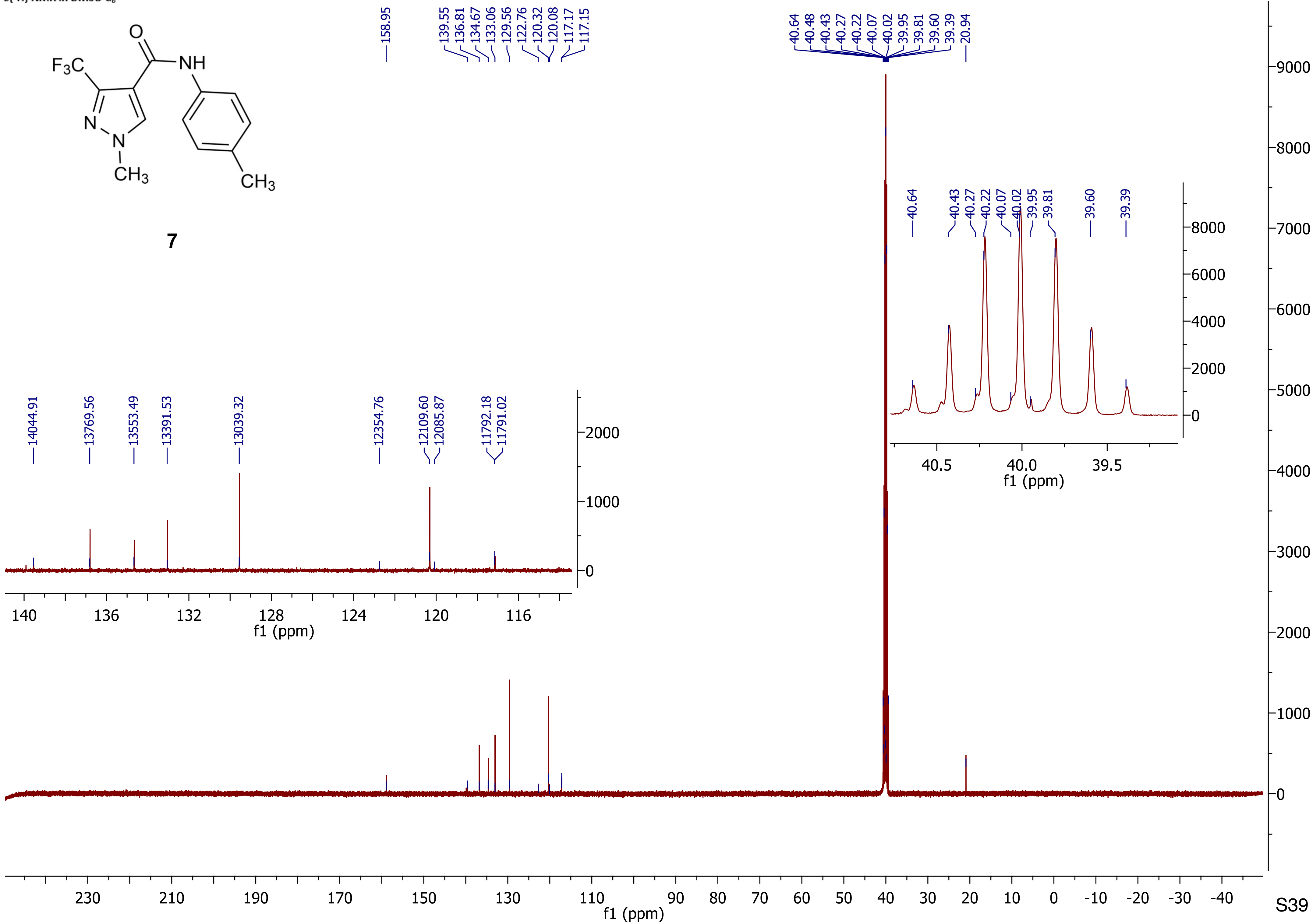

7

$^{19}F\{^{1}H\}$ NMR in DMSO-d_6

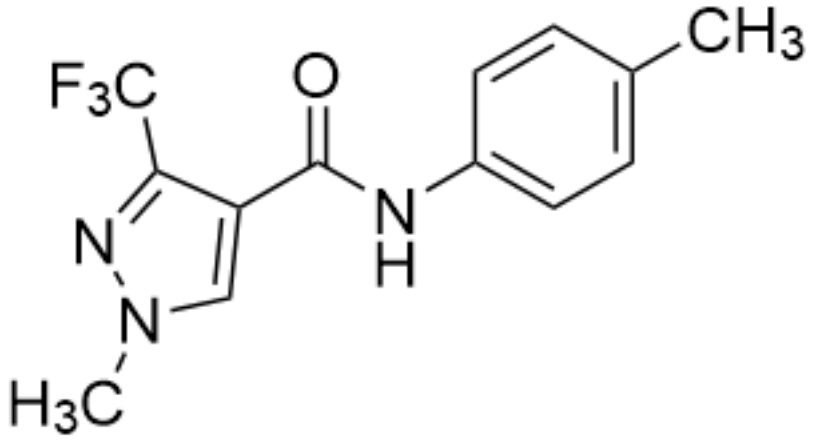

7

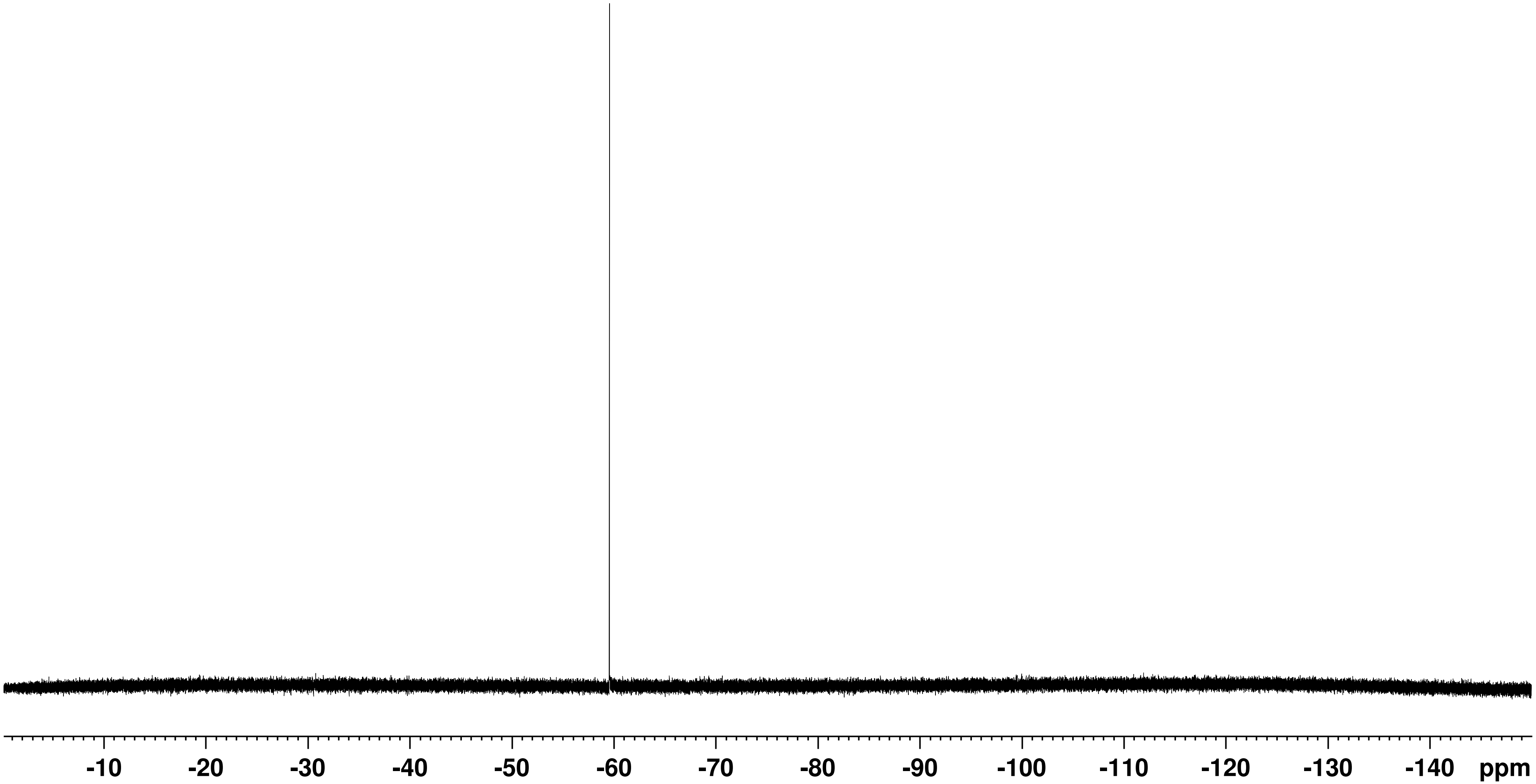

¹H NMR in DMSO-d_6

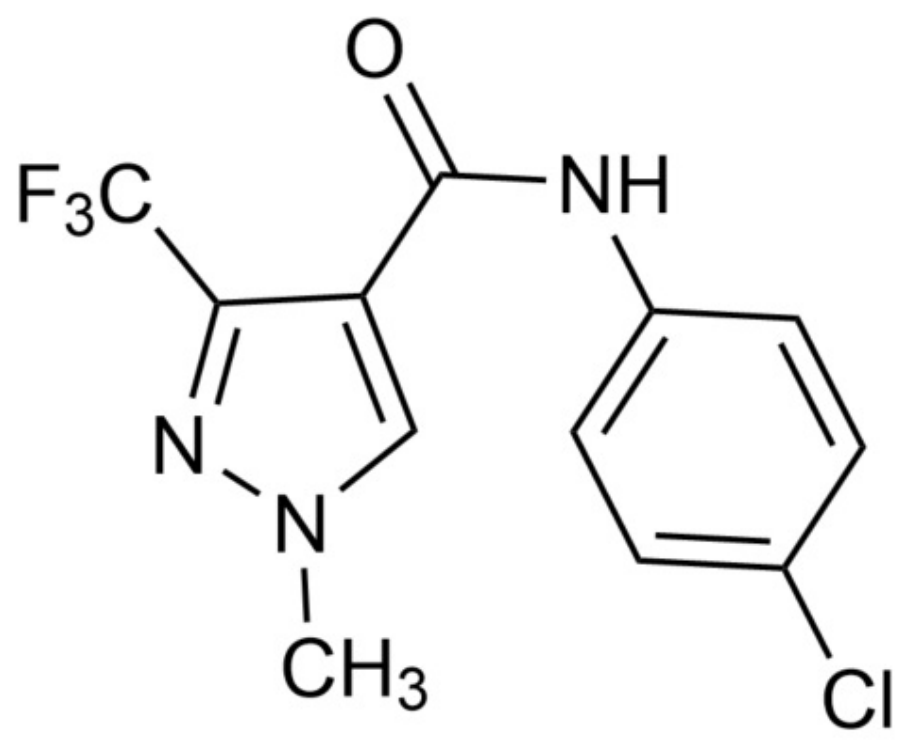

8

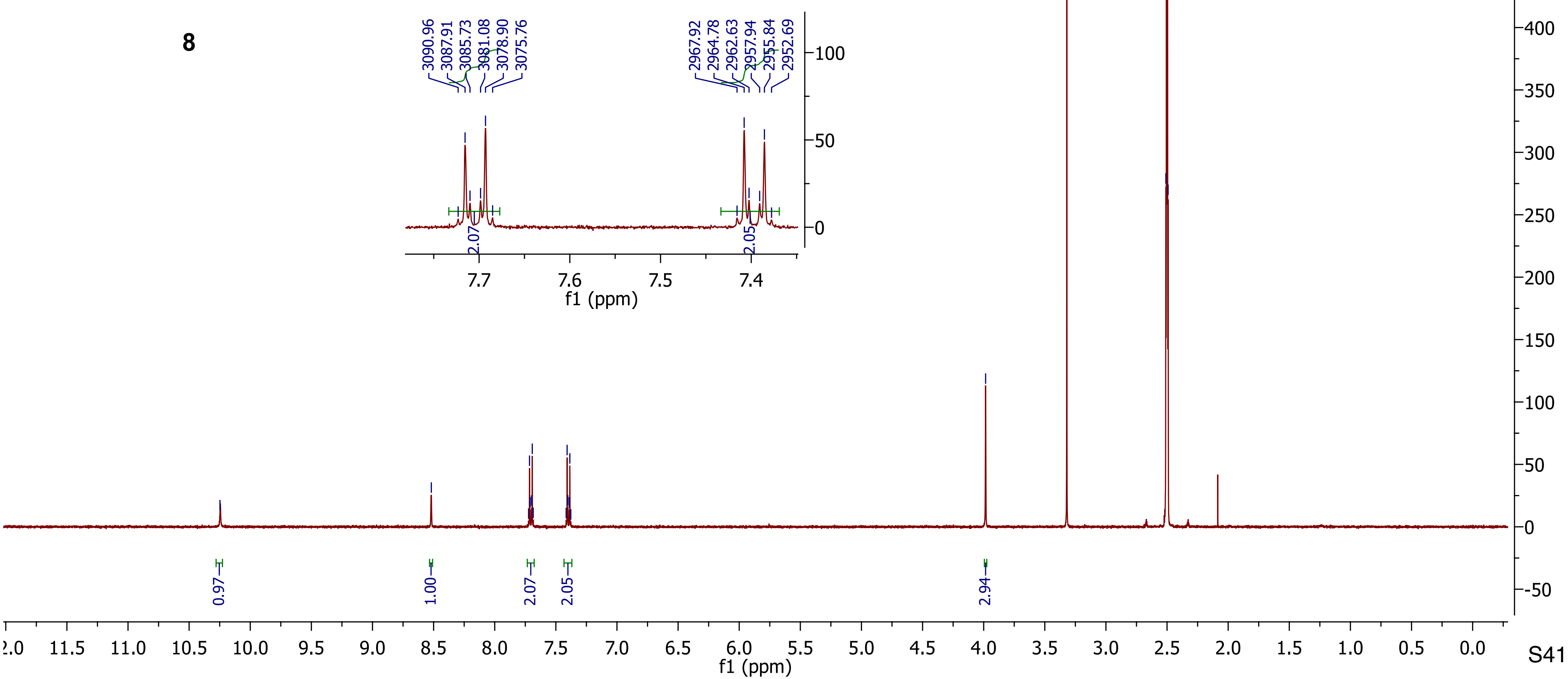
10.25
8.52
7.72
7.72
7.71
7.70
7.69
7.69
7.42
7.41
7.40
7.39
7.39
7.38
3.98
3.32
2.51
2.51
2.50
2.50
2.49
3090.96
3087.91
3085.73
3081.08
3078.90
3075.76
2967.92
2964.78
2962.63
2957.94
2955.84
2952.69
2.07
2.05
f1 (ppm)
0.97
1.00
2.07
2.05
2.94
f1 (ppm)

$^{13}C\{^{1}H\}$ NMR in DMSO-d_6

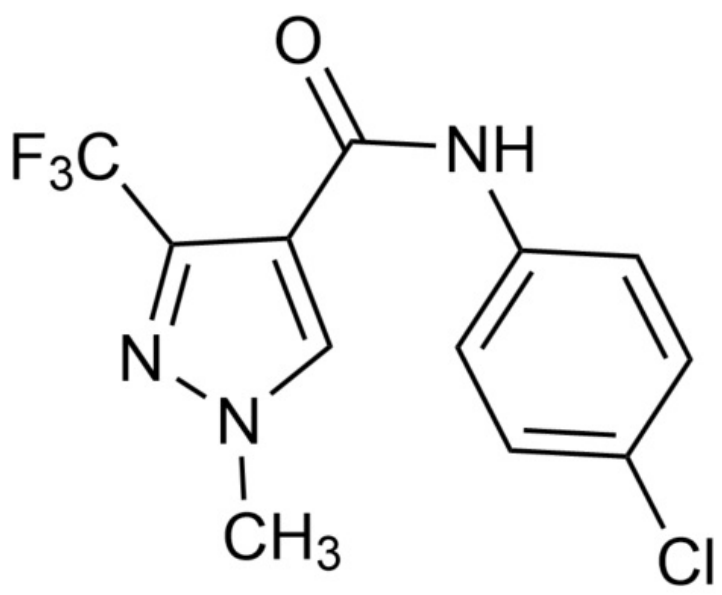

8

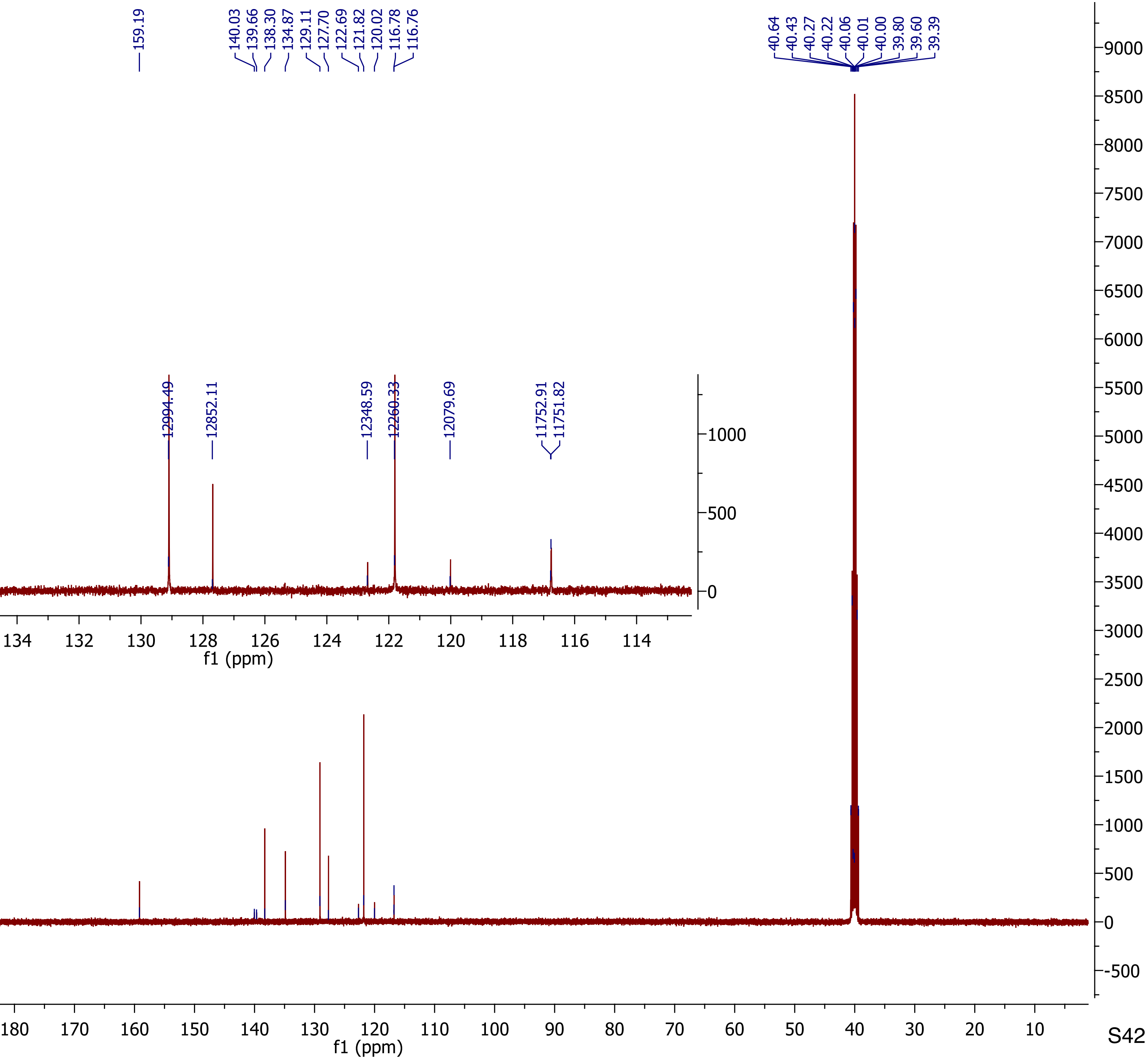

$^{19}F\{^{1}H\}$ NMR in DMSO-d_6

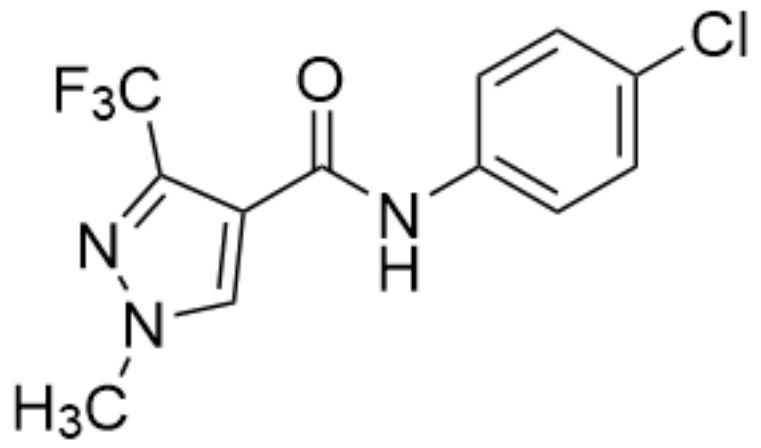

8

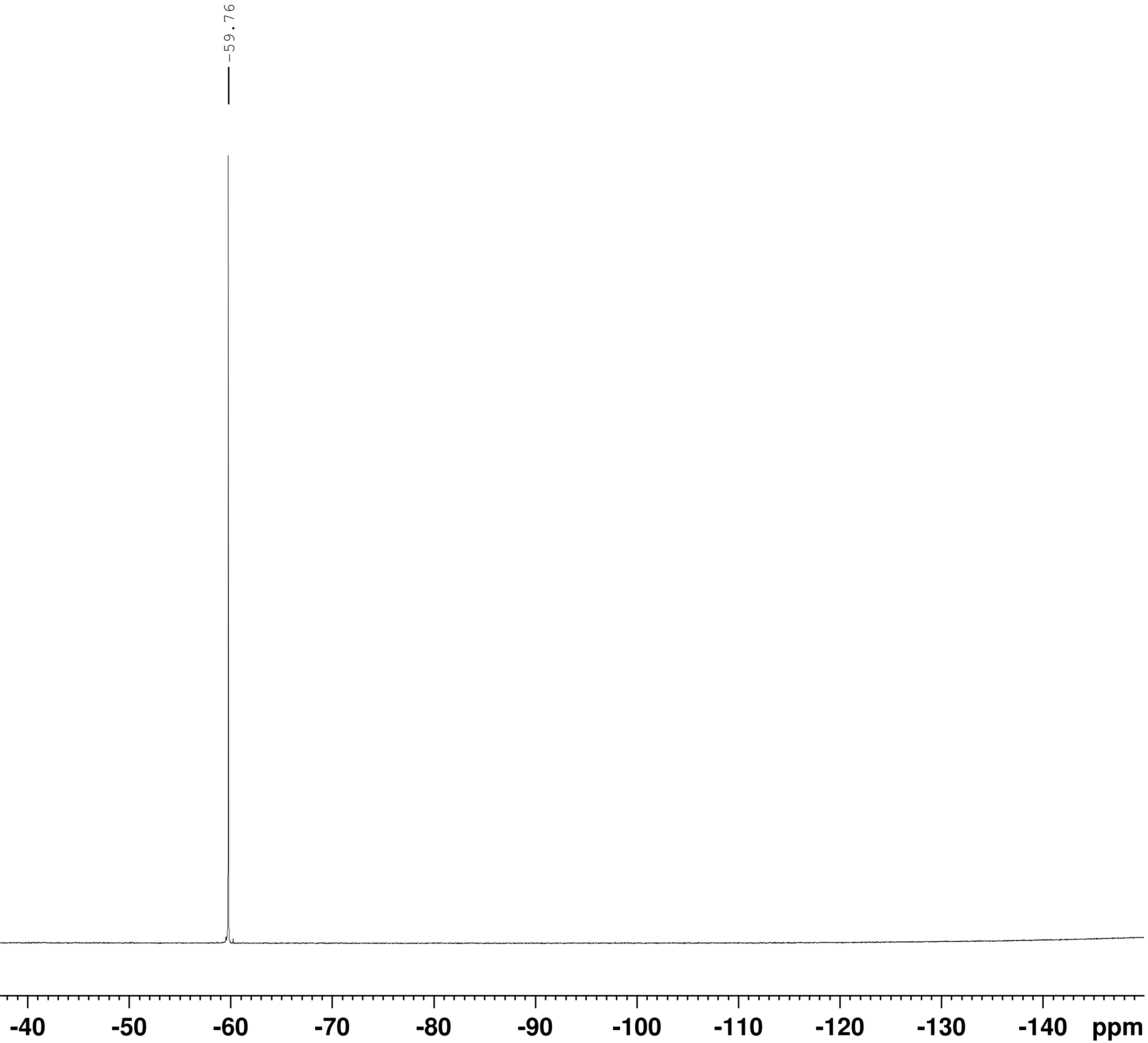

^1H NMR in $CDCl_3$

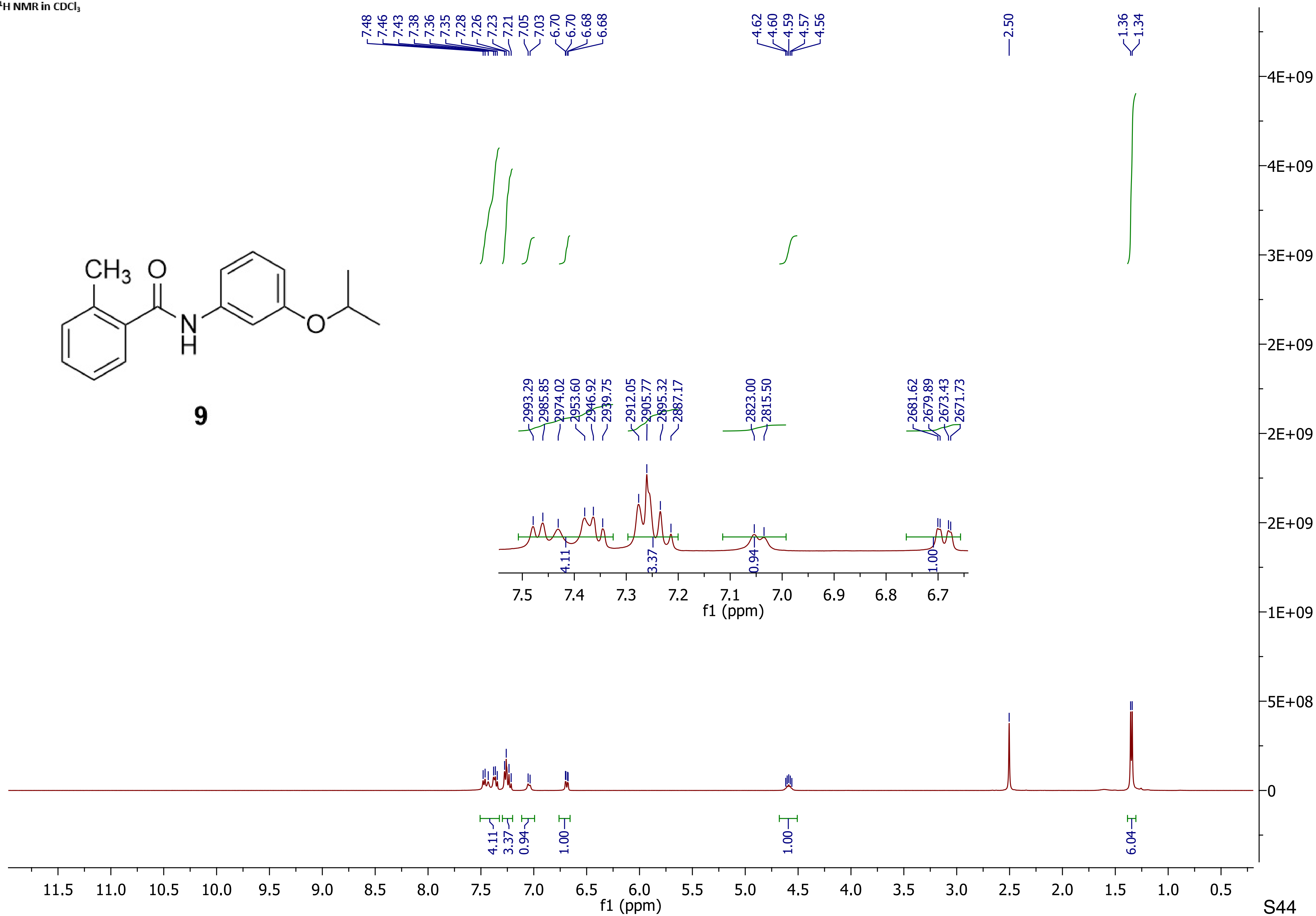

$^{13}C\{^1H\}$ NMR in $CDCl_3$

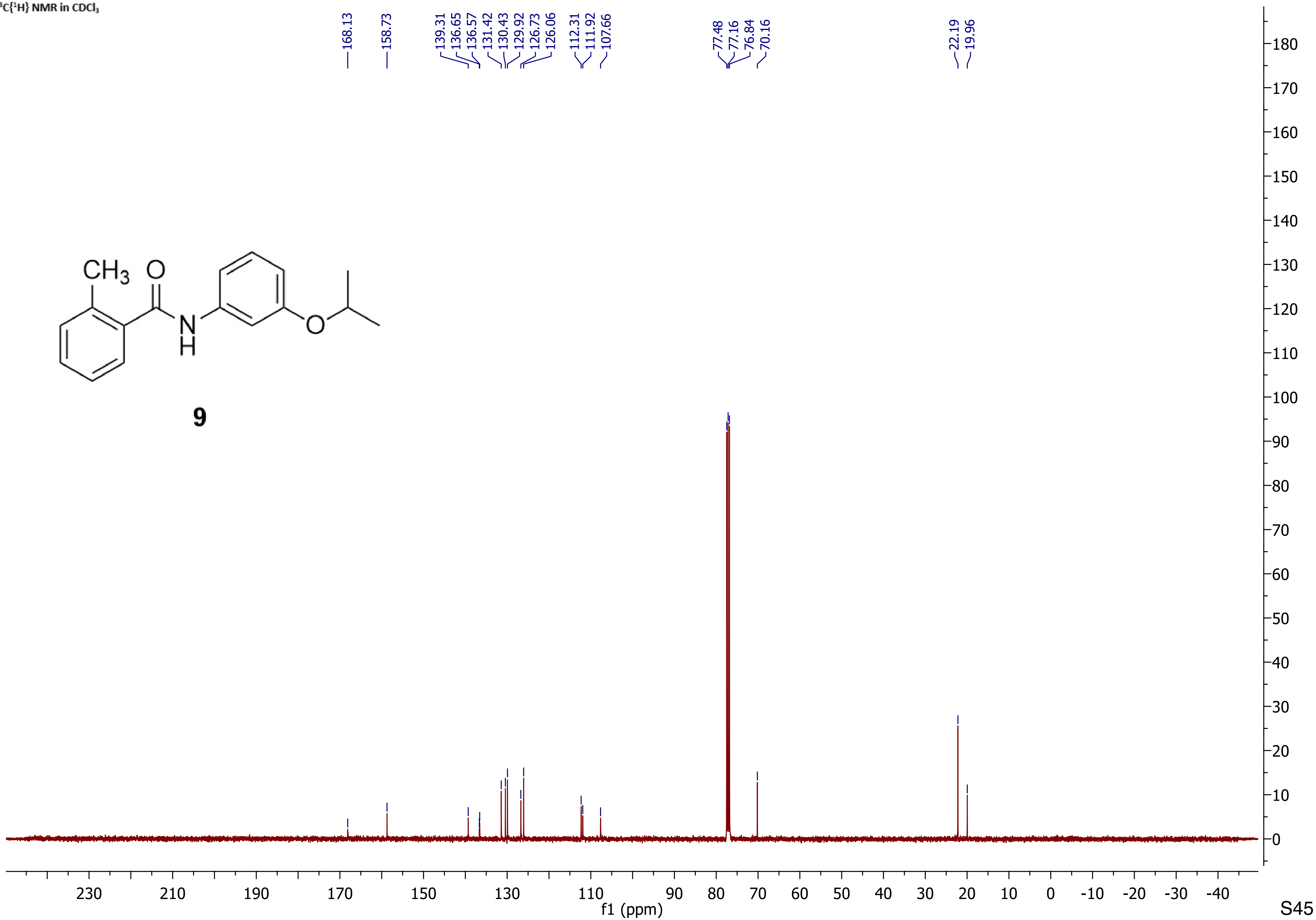

^{1}H NMR in DMSO-d_6

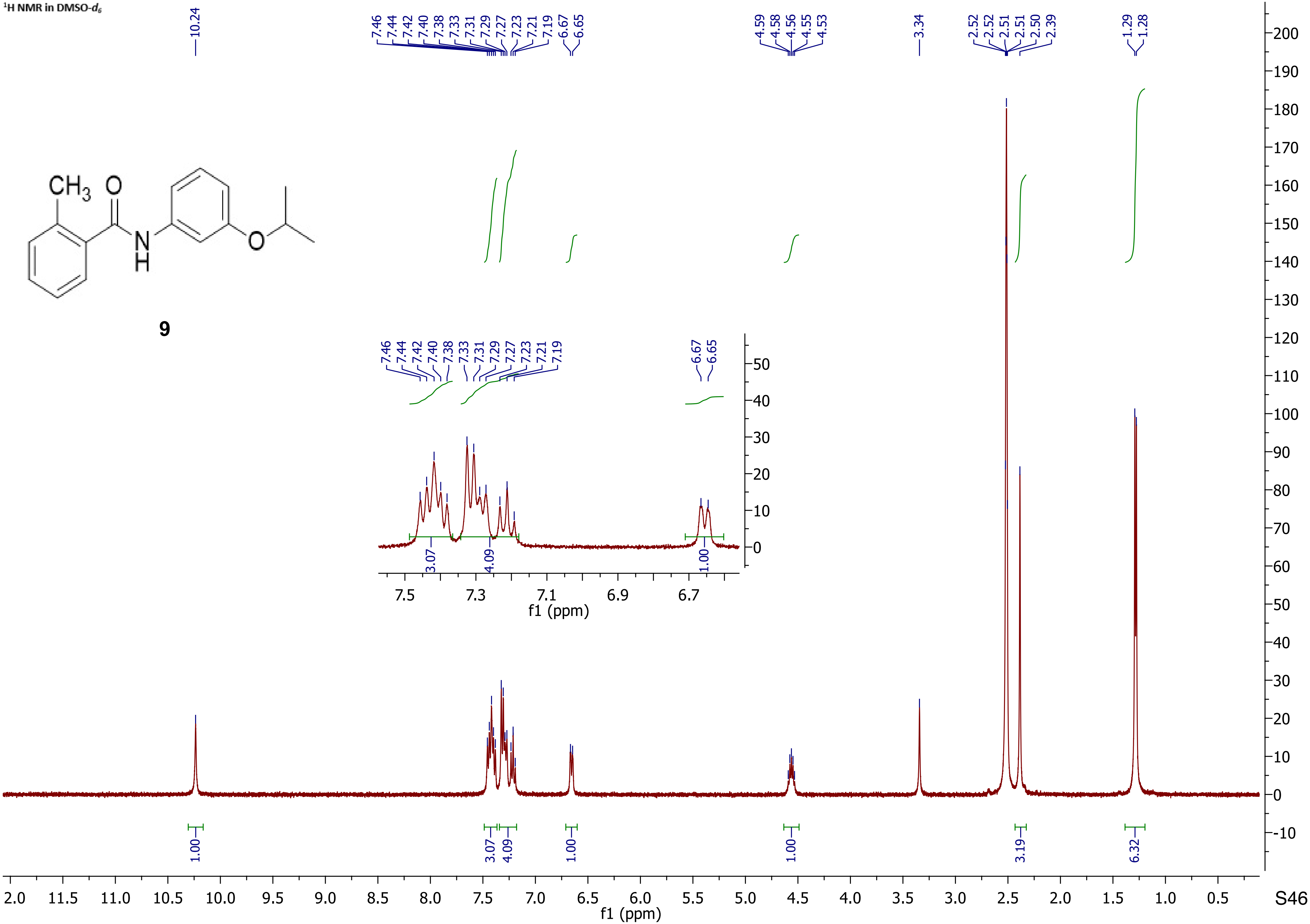

$^{13}C\{^1H\}$ NMR in DMSO-d_6

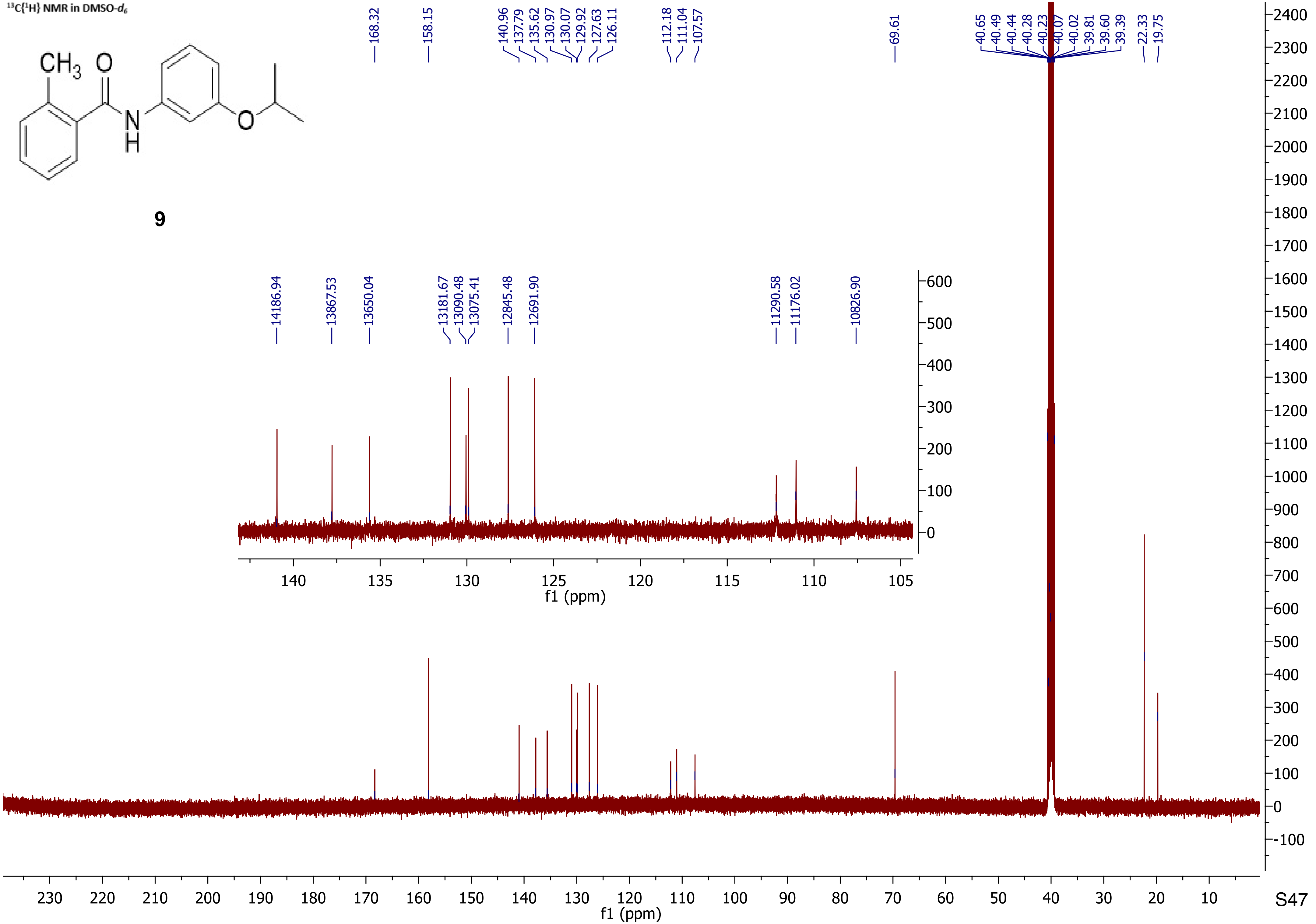

9

^1H NMR in $CDCl_3$

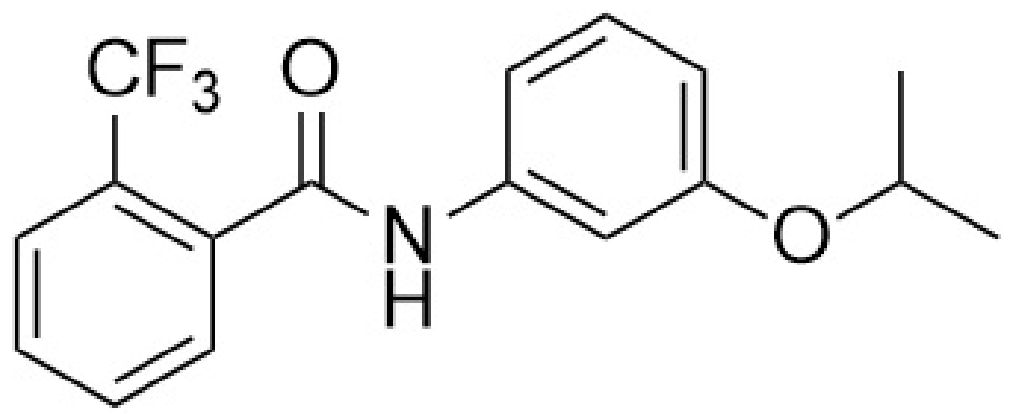

10

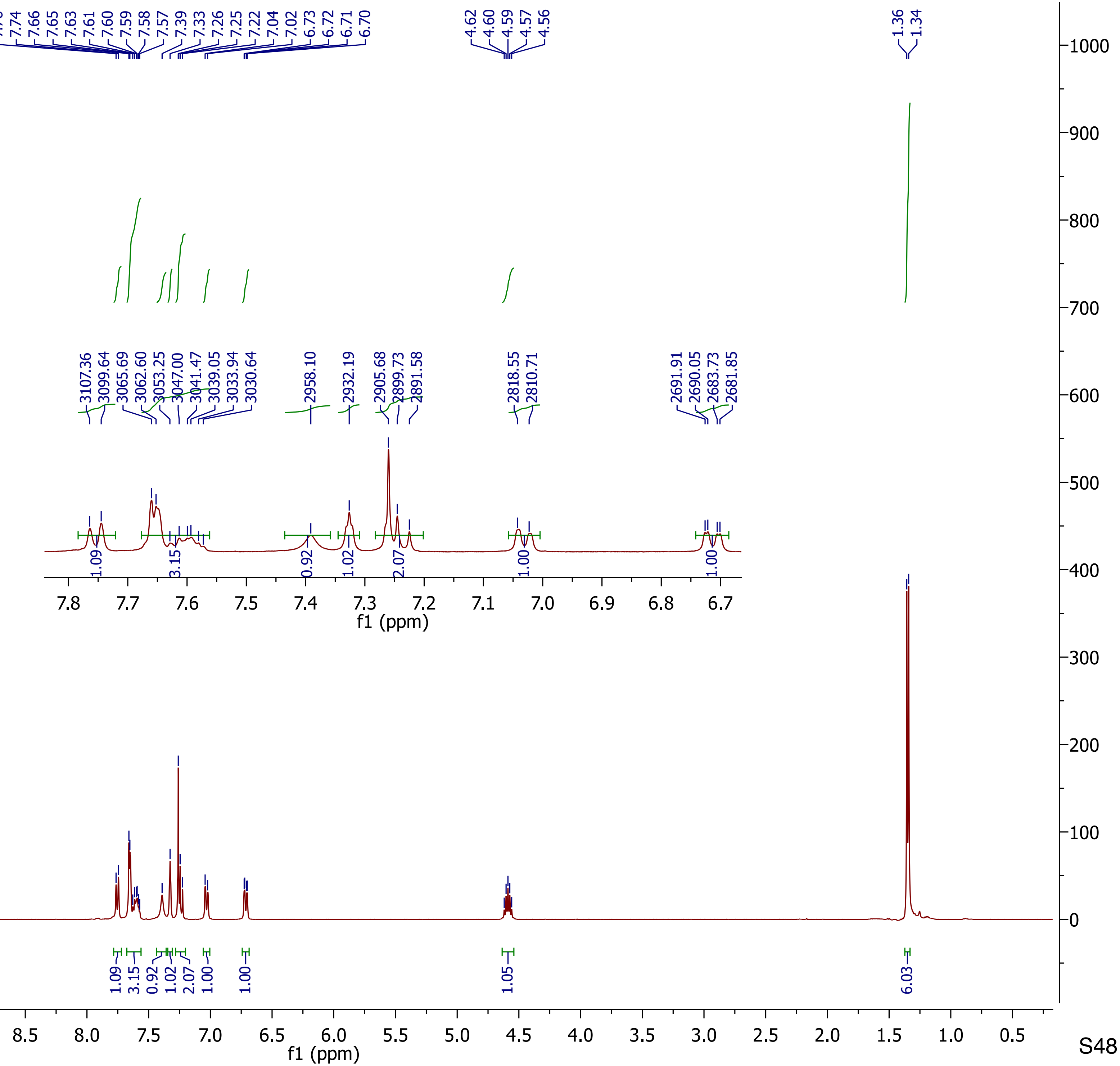

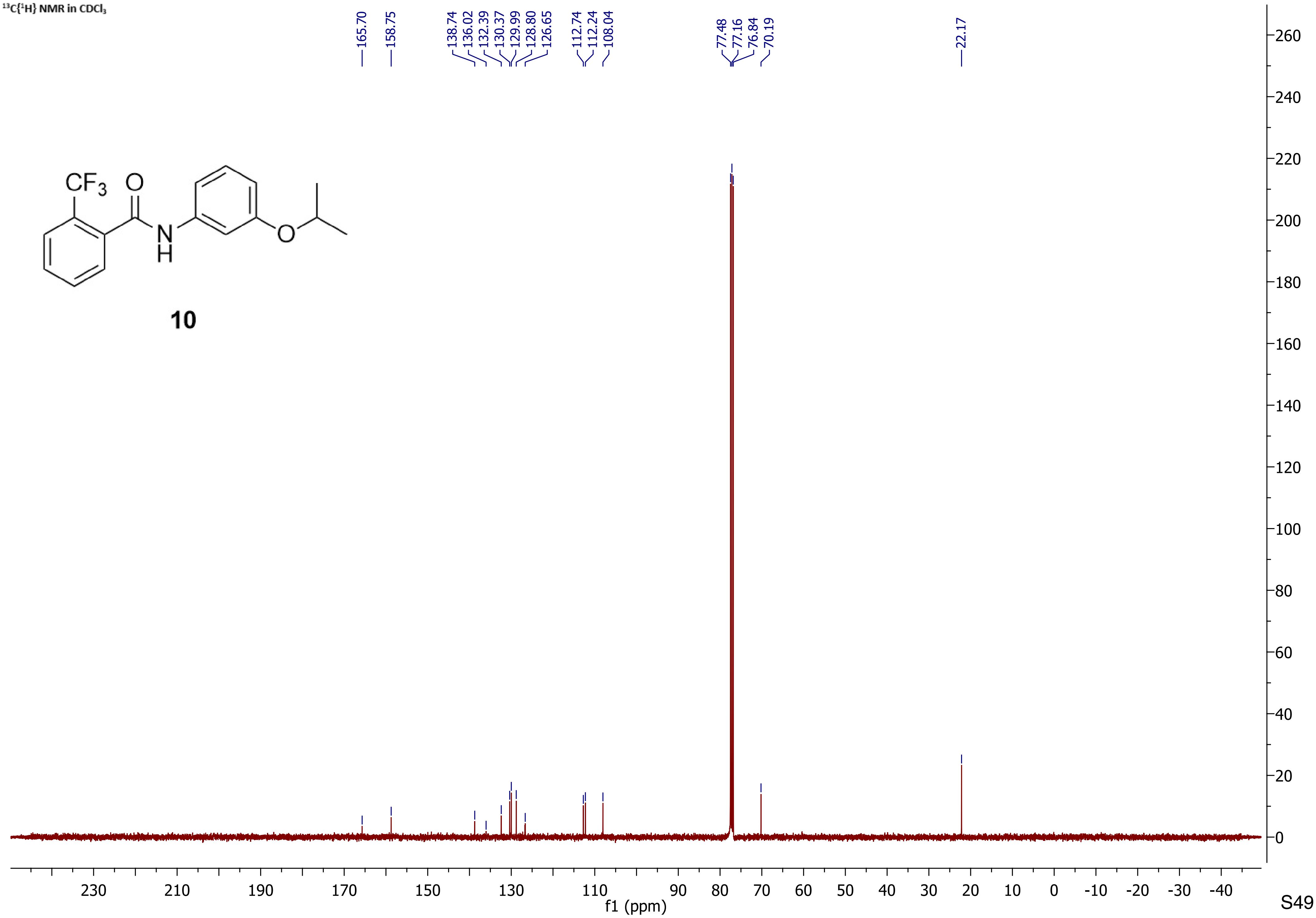

13C{1H} NMR in CDCl3
165.70
158.75
138.74
136.02
132.39
130.37
129.99
128.80
126.65
112.74
112.24
108.04
77.48
77.16
76.84
70.19
22.17
CF3
O
N
H
O
10
f1 (ppm)

^{1}H NMR in DMSO-d_6

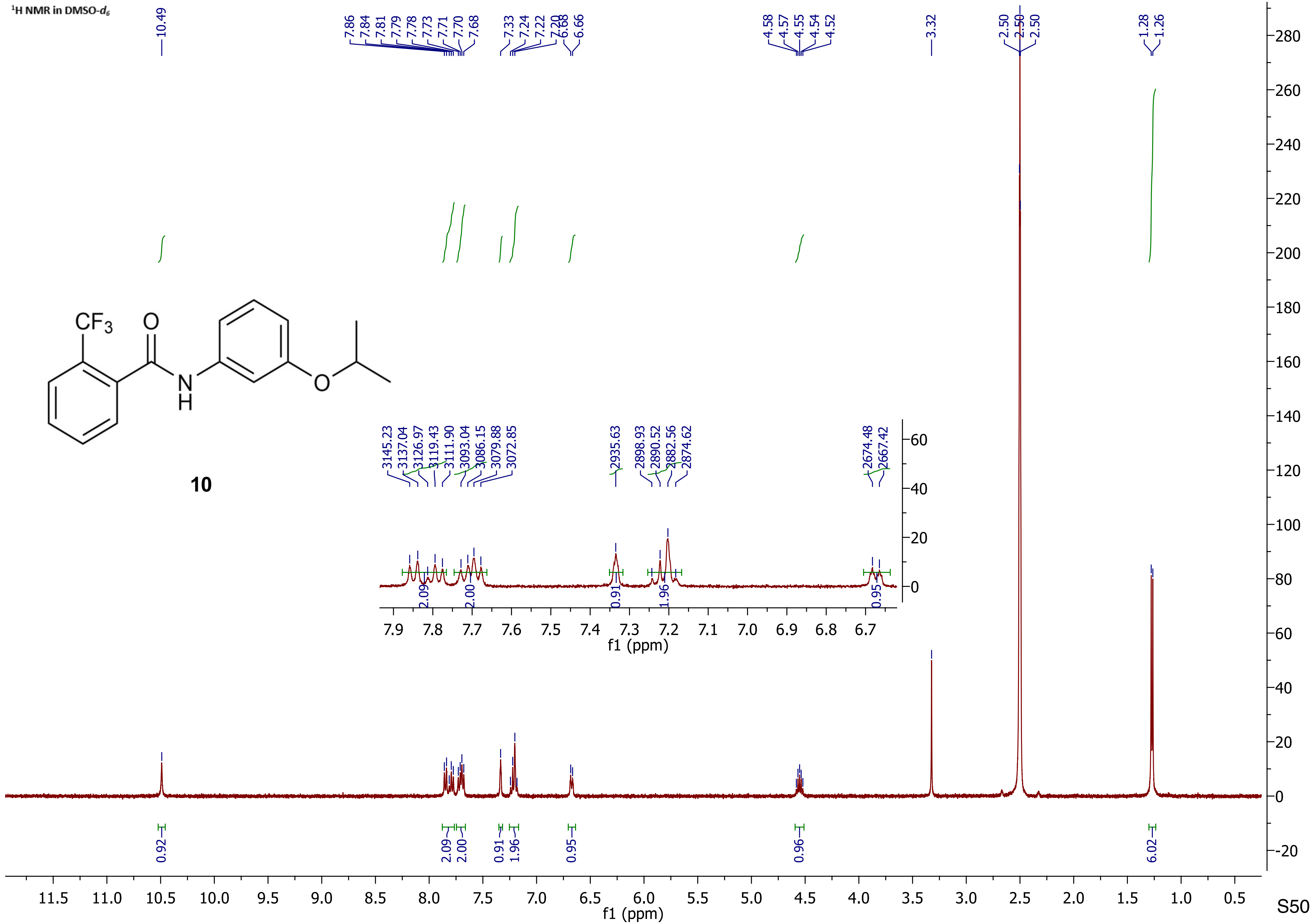

10

$^{13}C\{^1H\}$ NMR in DMSO-d_6

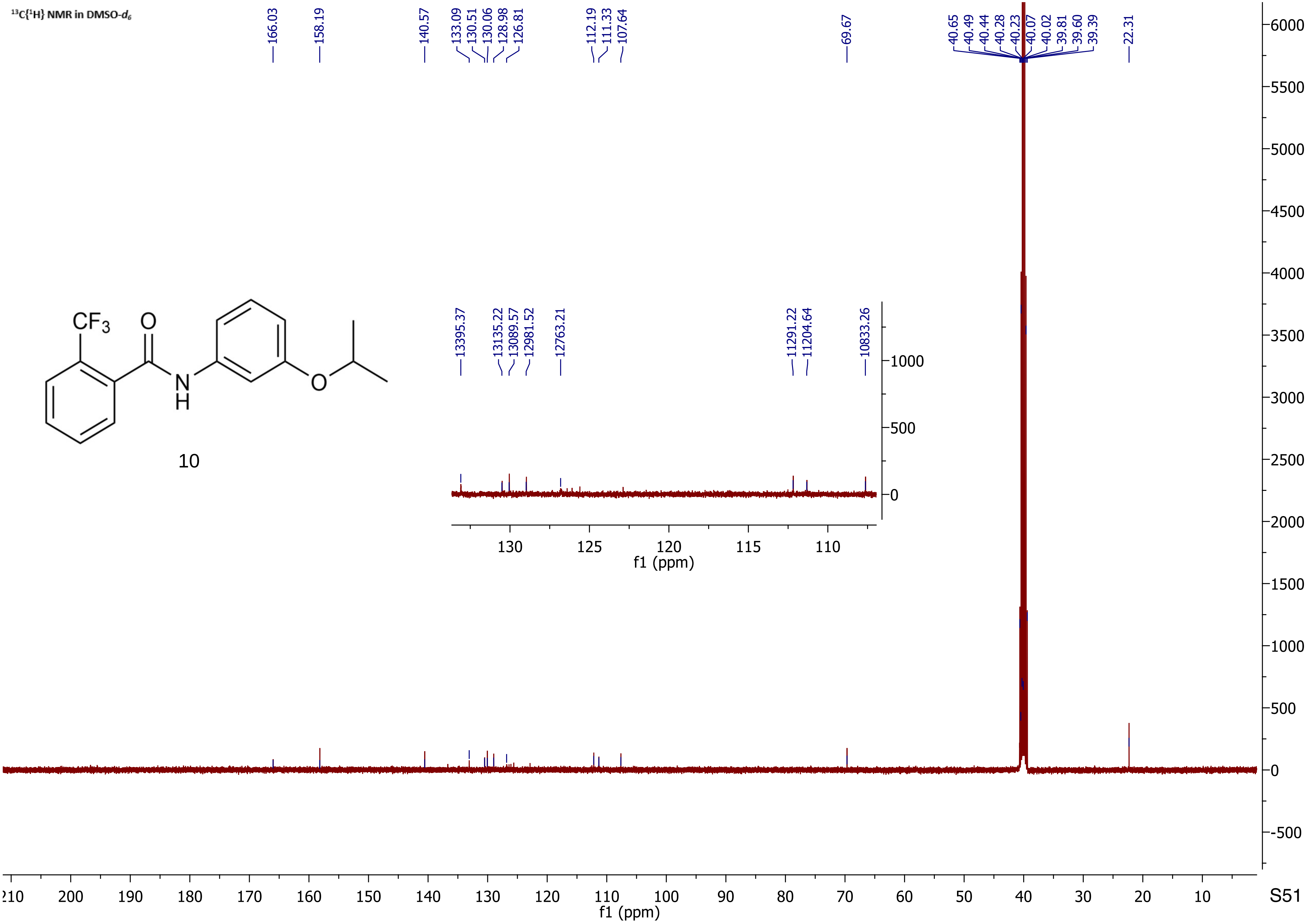

10

$^{19}F\{^{1}H\}$ NMR in DMSO-d_6

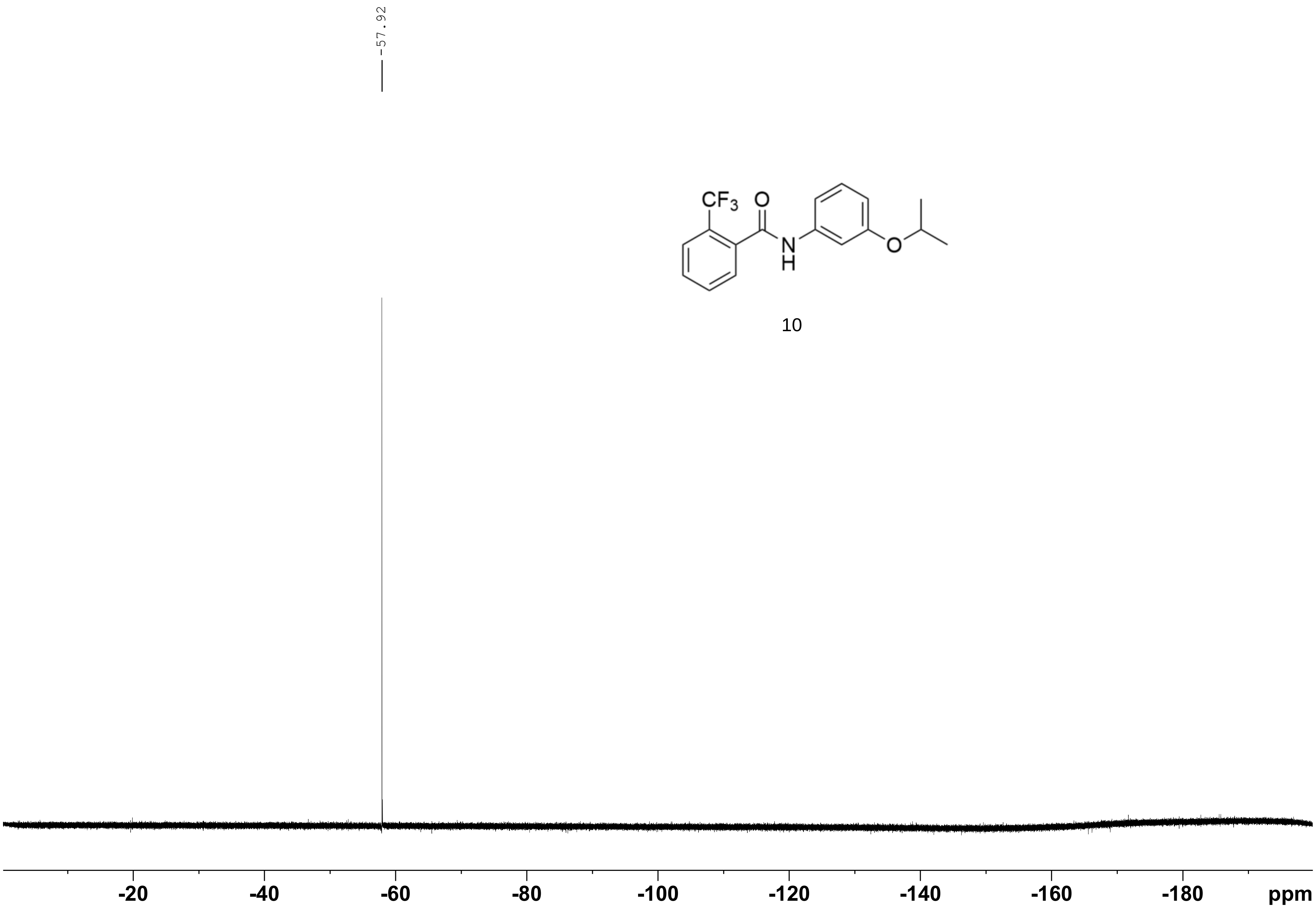

^1H NMR in $CDCl_3$

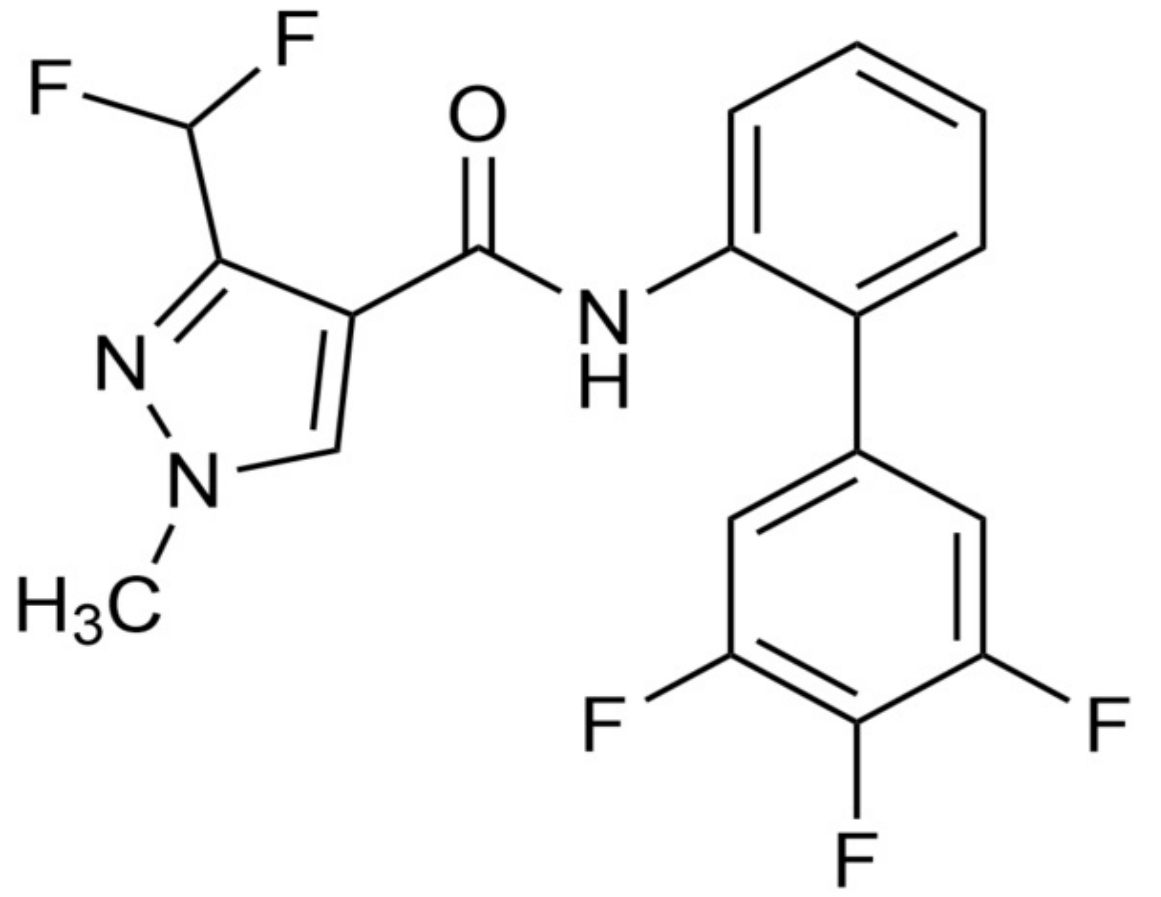

11

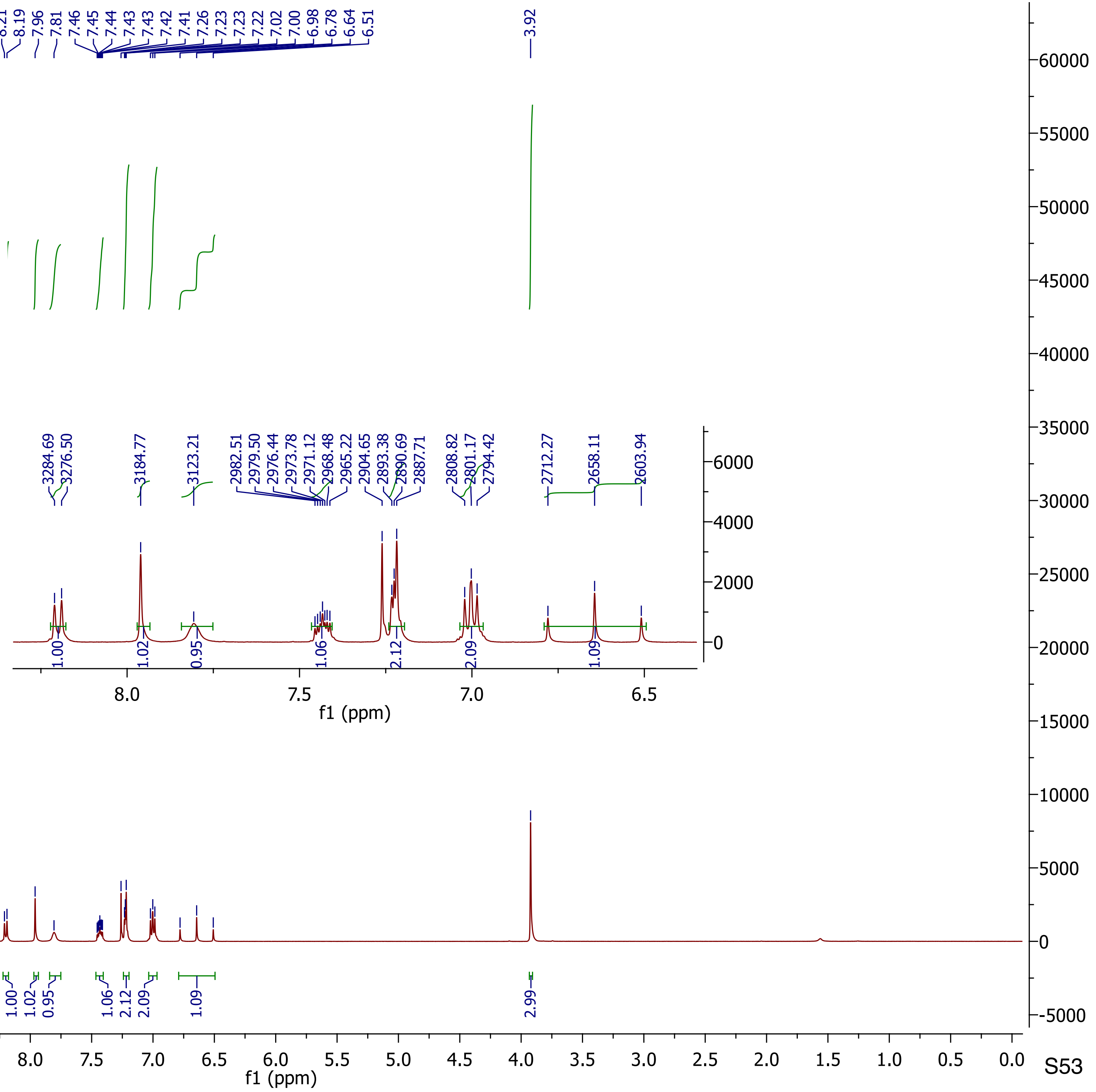

$^{13}C\{^{1}H\}$ NMR in $CDCl_3$

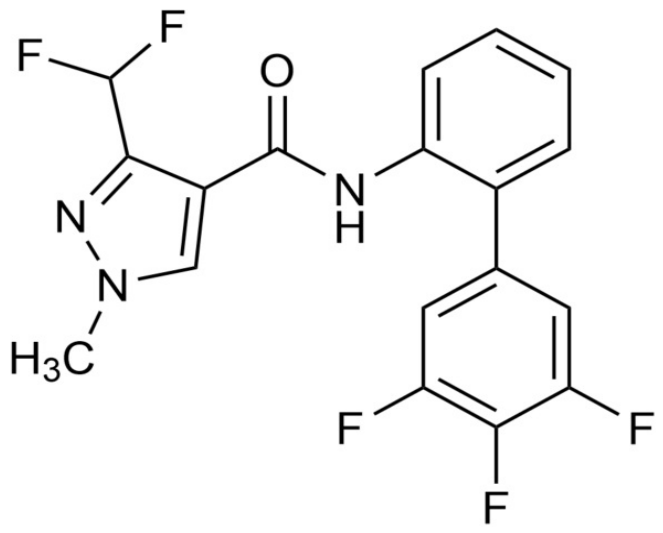

11

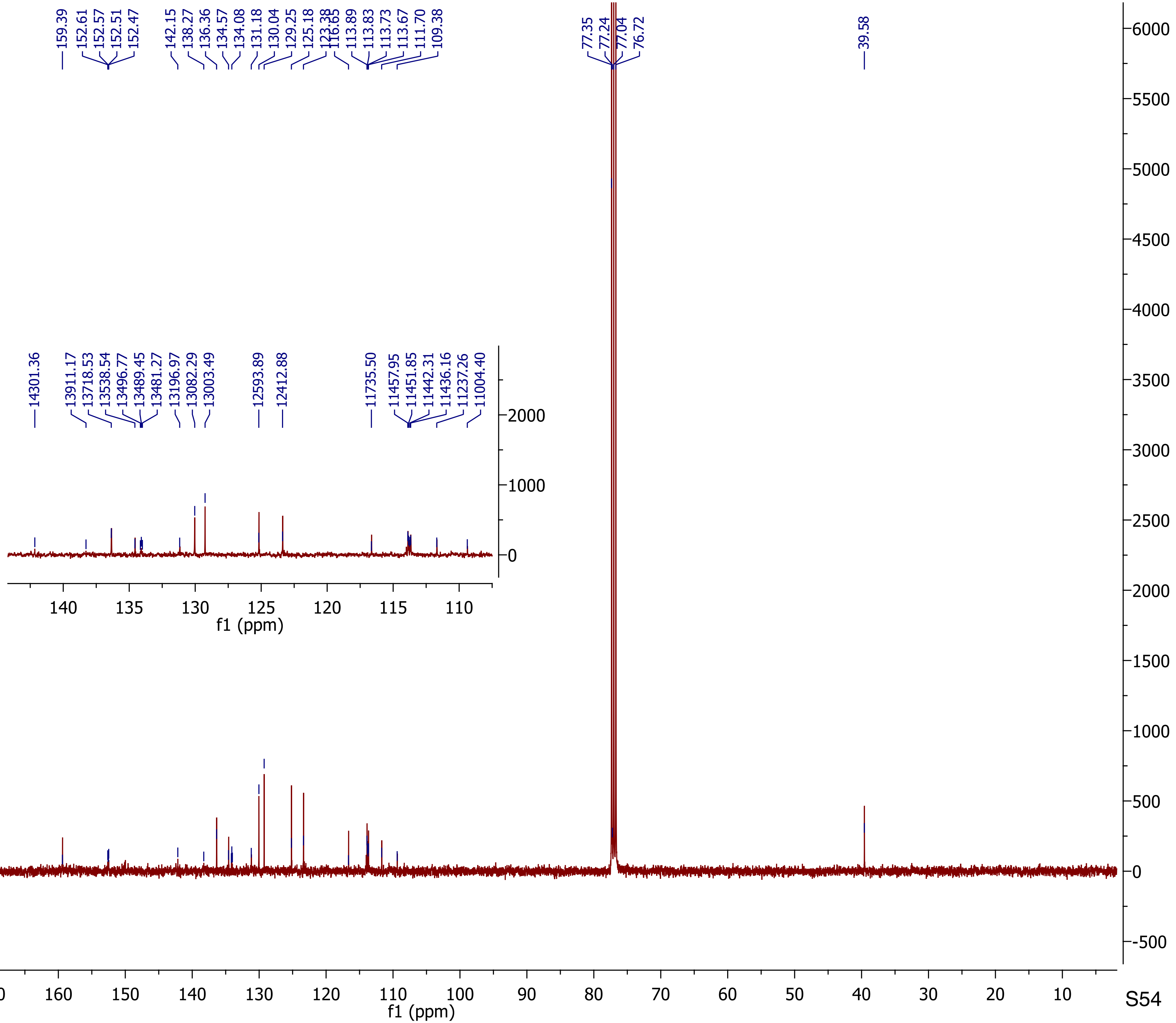

$^{19}F\{^1H\}$ NMR in $CDCl_3$

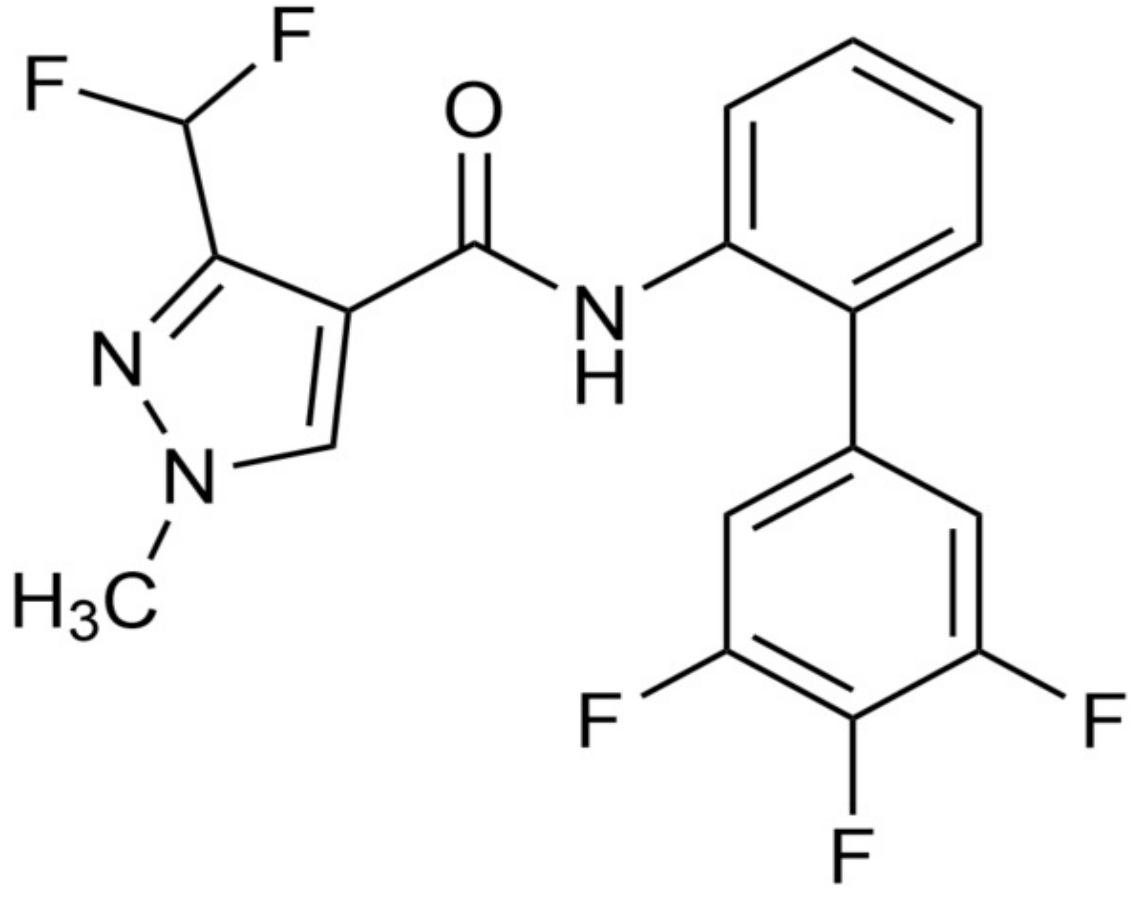

11

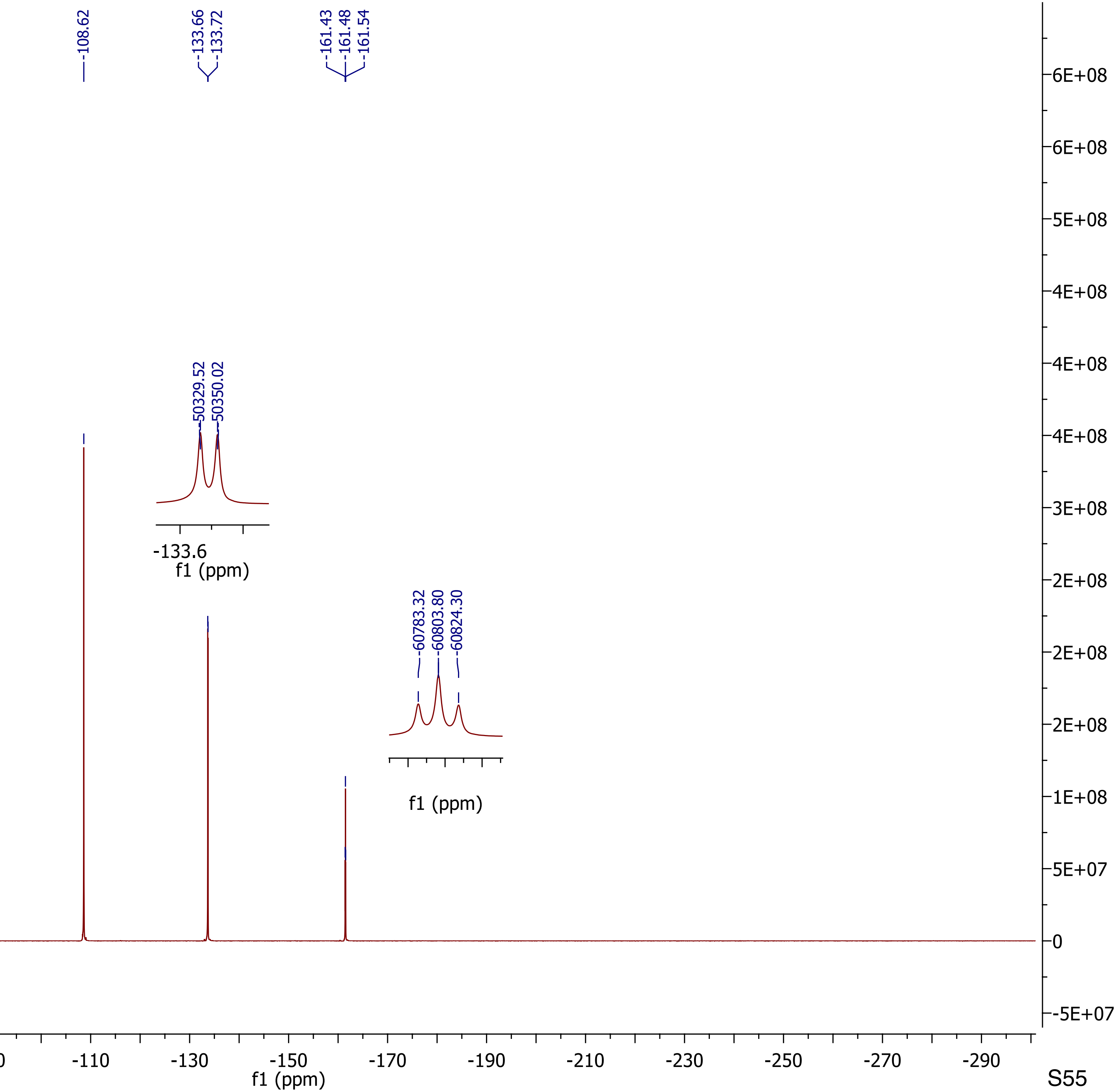

Supplement: Supplementary file 1 [file CHEM-32-e71190-s002.pdf]
